# Large scale physiological readjustment during growth enables rapid, comprehensive and inexpensive systems analysis

**DOI:** 10.1186/1752-0509-4-64

**Published:** 2010-05-14

**Authors:** Marc T Facciotti, Wyming L Pang, Fang-yin Lo, Kenia Whitehead, Tie Koide, Ken-ichi Masumura, Min Pan, Amardeep Kaur, David J Larsen, David J Reiss, Linh Hoang, Ewa Kalisiak, Trent Northen, Sunia A Trauger, Gary Siuzdak, Nitin S Baliga

**Affiliations:** 1 Institute for Systems Biology, 1441 North 34th Street, Seattle, WA 98103 USA; 2 Department of Biomedical Engineering and UC Davis Genome Center, University of California, Davis, One Shields Ave, Davis, CA 95616 USA; 3 Division of Genetics and Mutagenesis, National Institute of Health Sciences, Tokyo 158-8501, Japan; 4 Scripps Research Institute, 10550 North Torrey Pines Road, La Jolla, Ca 92037 USA

## Abstract

**Background:**

Rapidly characterizing the operational interrelationships among all genes in a given organism is a critical bottleneck to significantly advancing our understanding of thousands of newly sequenced microbial and eukaryotic species. While evolving technologies for global profiling of transcripts, proteins, and metabolites are making it possible to comprehensively survey cellular physiology in newly sequenced organisms, these experimental techniques have not kept pace with sequencing efforts. Compounding these technological challenges is the fact that individual experiments typically only stimulate relatively small-scale cellular responses, thus requiring numerous expensive experiments to survey the operational relationships among nearly all genetic elements. Therefore, a relatively quick and inexpensive strategy for observing changes in large fractions of the genetic elements is highly desirable.

**Results:**

We have discovered in the model organism *Halobacterium salinarum *NRC-1 that batch culturing in complex medium stimulates meaningful changes in the expression of approximately two thirds of all genes. While the majority of these changes occur during transition from rapid exponential growth to the stationary phase, several transient physiological states were detected beyond what has been previously observed. In sum, integrated analysis of transcript and metabolite changes has helped uncover growth phase-associated physiologies, operational interrelationships among two thirds of all genes, specialized functions for gene family members, waves of transcription factor activities, and growth phase associated cell morphology control.

**Conclusions:**

Simple laboratory culturing in complex medium can be enormously informative regarding the activities of and interrelationships among a large fraction of all genes in an organism. This also yields important baseline physiological context for designing specific perturbation experiments at different phases of growth. The integration of such growth and perturbation studies with measurements of associated environmental factor changes is a practical and economical route for the elucidation of comprehensive systems-level models of biological systems.

## Background

One of the main goals of current molecular systems biology is to generate quantitative models for the structure and behavior of cellular, metabolic, signal transducing and gene regulatory networks in a complete organism. Such knowledge may enable us to ultimately predict global changes in gene expression of an organism in response to cellular stimuli and enable the predictable reengineering of cells for therapeutic or industrial purposes [1]. The relative simplicity of microbial genomes and the shrinking cost of measuring their complete transcriptomes has made deciphering structures of gene regulatory influence networks and detecting functional relationships within nearly genomically-complete data sets feasible [2-5]. One of the main challenges to using these techniques broadly, however, is that many observations of the cellular components to be modeled are required. This can be both time and resource intensive. Therefore, a general approach that allows the observation of changes in many cellular components in few experiments is highly desired.

In this light, it is worthwhile to recall that for nearly a century the microbial growth curve has been instrumental in shaping our investigation and understanding of cell function and evolution [6-8]. The power of studying simple batch growth results at least partially from the fact that batch growth is a fundamentally complex process. Cells growing in batch culture experience fluctuations in numerous environmental parameters (i.e. nutrients, oxygen, toxins etc.), changes that they themselves induce through metabolism. In response to these self-mediated changes in their local environment cells undergo numerous changes in physiology, re-regulate cell division and reorganize cellular infrastructure (e.g. by changing cytoskeletal structure and membrane composition etc.). As a consequence, studies of growth have contributed enormous insight into the growth-related role(s) of specific genes, processes and metabolic pathways in model eukaryotes, bacteria and archaea.

Among the archaea, *H. salinarum *NRC-1 is one of few that is easily cultured and manipulated in the laboratory. For this reason it has become an important model system for characterizing archaea with respect to their global cellular responses to diverse stresses [9-15], architecture of gene regulatory networks [16], their proteomes [17], transcriptome structure [18] and aspects of post transcriptional regulation [19], archaeal DNA replication [20], archaeal DNA repair [21-23], archaeal rhodopsins [24-26], archaeal cell cycle [27] and numerous other topics. However, and somewhat surprisingly, despite the relative abundance of studies, including global analyses, the growth associated physiologies during batch culture of *H. salinarum *NRC-1 have only received basic characterization on a global scale. The work by Lange et al. [19], comes closest to doing so but only samples cells at two points during growth (exponential and stationary phases) and thus provides only a limited view into dynamic growth-related physiological changes.

This manuscript reports a systems-level interrogation of physiological changes that occur in *H. salinarum *NRC-1 during growth in batch cultures. Our analysis demonstrates the scale at which growth in simple, reproducible batch cultures can induce major physiological changes encoded by 1,518, or 63% of the approximately 2400 putatively identified non-redundant genes in *H. salinarum *NRC-1. Transcriptional changes are interpreted in the context of biological functions. In addition this manuscripts demonstrates how the analysis of transcriptome growth data in the context of other data types, including metabolomic data and gene perturbation can lead to novel biological insight.

## Results and Discussion

### Growth of *H. salinarum *NRC-1

**Figure 1:**
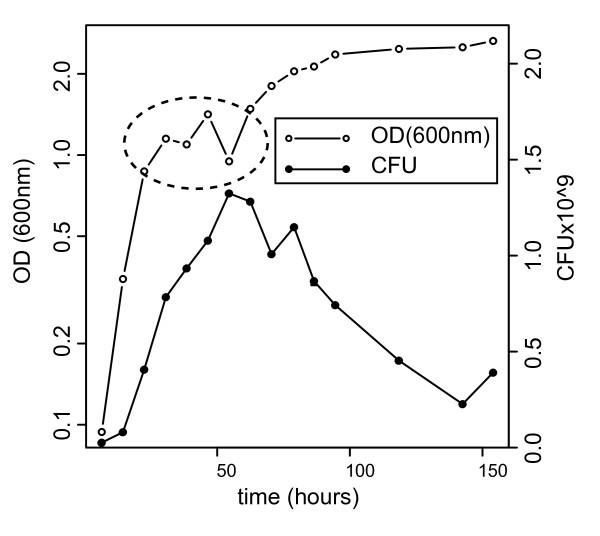
**Growth and survival of *H. salinarum *NRC-1 in rich media**. The growth and survival of *H. salinarum *NRC-1 in Complete Medium (CM) (see materials and methods for composition and growth conditions) was monitored by changes in optical density at 600 nm (OD_600_) and by replica plating. The left y-axis shows growth versus time as measured by OD_600 _on a log scale. The corresponding OD_600 _data is plotted as open circles. The right y-axis indicates the number of colony forming units (CFU) measured from samples versus time. The corresponding CFU data is plotted as closed circles. The dotted oval highlights an apparent growth phase transition.

**Figure 2:**
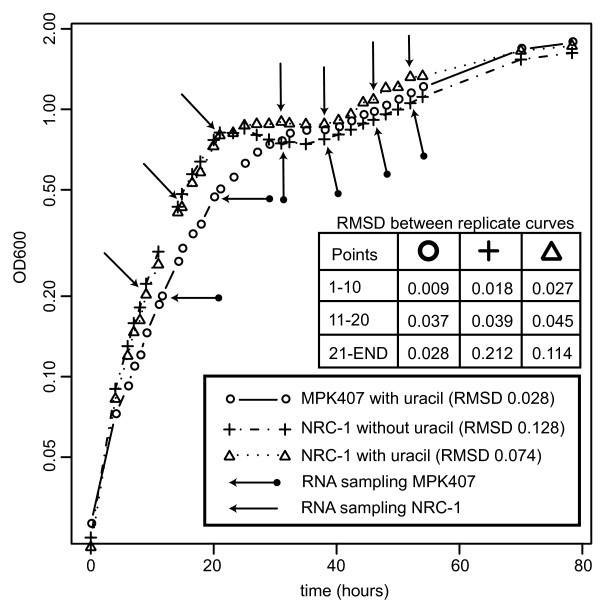
**Growth of *H. salinarum *NRC-1 and MPK407 strains for microarray analysis**. *H. salinarum *NRC- 1 and MPK407 strains were grown according to materials and methods as duplicate cultures. Average optical density at 600 nm (OD_600_) values for the duplicate cultures are plotted for simplicity. The root mean square distance between replicate measurements over the entire growth curve is listed in the figure legend. A table lists root mean square distances (RMSD) between replicates growth curves for sampling points 1-10, 11-20 and 21- the last point. *H. salinarum *NRC-1 was grown both in the presence and without extra exogenous uracil. The MPK407 strain was only grown in the presence of extra exogenous uracil. Arrows indicate sampling points for totalRNA extraction.

We first characterized growth of *H. salinarum *NRC-1 in rich complete medium (CM) (see Materials and Methods for details). A typical growth curve is shown in Figure 1 and Additional file 1, Figure S1A. Consistent with the observation previously made by Shand and Betlach [28] the curve appears to have biphasic characteristics (for an additional example see also Figure 2). Several other possibilities exist to explain this growth behavior, including the the release of gas vesicles from dying cells (for a detailed explanation see Additional file 2) and cellular clumping. Relatively little clumping is observed by visible light microscopy and the appearance of free floating gas vesicles in the media coincides with the noted increase in optical density at 600 nm.

### Microarray analysis of gene expression changes during growth

We monitored mRNA changes (relative to a reference RNA sample from a mid exponential growth culture of the wild type strain) at 7 different points along the growth curve for two cultures, the wild type strain in standard growth medium with or without uracil supplementation and 6 different points for strain MPK407, a *pyrF *uracil auxotroph mutant of *H. salinarum *NRC-1, supplemented with uracil (Figure 2). We note that changes in transcript abundance alone do not necessarily reflect changes at the protein level or the potential functional effects of translational and post translational regulatory mechanisms. Nevertheless, in general, changes in transcriptional abundance are good indicators of the relevance of an encoded function and good approximation that the associated functions may be occurring of at the time of measurement [13,15]. The following analysis is approached from this perspective.

### Aggregate analysis of data

**Figure 3:**
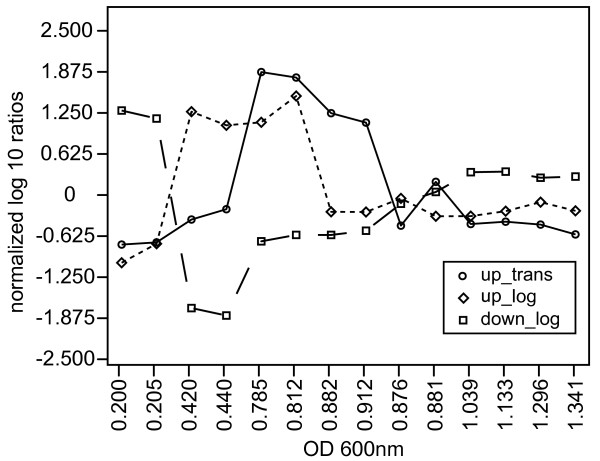
**Average expression profiles for gene groups showing differential patterns of gene expression**. The average expression profiles for the three small sets of distinctive expression profiles (a) genes whose transcripts are transiently increased during exponential growth (b) genes whose transcripts are transiently decreased during exponential growth and (c) genes whose transcripts are transiently increased during the transition between exponential and stationary phases are plotted for one of the three datasets. This shows that sequential regulation of physiological processes occurs during growth and in the transition to stationary phase.

The aggregate gene expression data set was filtered by lambda value, a measure of statistical significance of change [29]. Changes in expression were considered to be significant if lambda was greater than 15 for at least four consecutive data points across the growth curve, including duplicate samples. By this conservative criterion, at least 1518, 63% of the approximately 2400 putatively identified non-redundant genes in *H. salinarum *NRC-1 showed significant changes in gene expression over the growth curve. We applied K-means clustering to this filtered data set to identify dominant patterns of gene expression changes. The two patterns with the largest gene memberships were those typified by (a) elevated gene expression during exponential growth followed by severe decreased transcript abundance in stationary phase (451 genes - Additional file 3: Figure S2A and Additional file 4: Table S1) and (b) depressed gene expression during exponential growth followed by dramatically increased transcript abundance in stationary phase (772 genes -See Additional file 3: Figure S2B and Additional file 5: Table S2). These two groups accounted for 81% of the genes showing significant changes in transcript levels. Three other interesting patterns were noted: (a) genes whose transcripts transiently increase in abundance during exponential growth (b) genes whose transcripts transiently decrease in abundance during exponential growth and (c) genes whose transcripts transiently increase in abundance during the transition between exponential and stationary phases. Figure 3 depicts the average expression profiles for each of these three smaller expression groups. This demonstrates that despite the complex physiological transitions induced by concurrent changes in numerous environmental factors, it is possible to detect unique and temporally separated components of the cellular response. Each of these expression pattern categories is described in more detail below.

### Expression patterns unique to the MPK407 strain

Differences in transcriptional profiles of strains MPK407 and wild-type *H. salinarum *NRC-1 were investigated. MPK407 is a routinely used genetic background for constructing in frame gene replacements [30]. Since MPK407 is used as the host for knock-out mutations and associated environmental perturbation experiments it is interesting to know, in general, how it differs from wild-type *H. salinarum *NRC-1. While this strain was initially derived from *H. salinarum *NRC-1 it was constructed through genetic manipulation and has been maintained in the laboratory. Therefore the genotype may differ from *H. salinarum *NRC-1 by more than the *pyrF *deletion and any differences detected by this analysis may not be solely due to the *pyrF *deletion. We conducted a between group t-test within the TM4 microarray analysis suite [31] to identify genes whose expression levels could be significantly different between the MPK407 and wild-type strains grown in the presence of additional uracil. Individual P-values were computed by permutation, with an overall critical alpha = 0.01 and false discovery control was applied by limiting the proportion of false significant genes to not exceed 0.01. This analysis was conducted on a filtered set of genes (described in Materials and Methods) that were deemed to be significantly differentially regulated across this complete data set.

**Figure 4:**
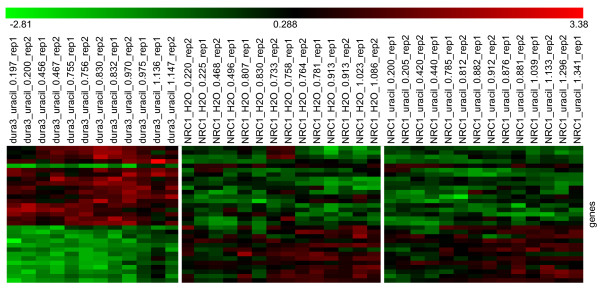
**Heatmap of genes selected by t-test analysis which differentiate between MPK407 and wild-type expression profiles**. A heatmap of the 31 genes whose expression profiles differentiate between MPK407 knockout strain and wild-type by t-test analysis. Analysis was performed using tools available in the TM4 suite of microarray analysis tools [31]. Genes (rows) are listed, from top to bottom, in each of the two panels in the same order that they appear in table 1. Experimental samples (columns) are listed in order of increasing optical density moving from left to right. The optical density for each sample is indicated in the sample name.

Using these criteria, we found 31 genes (Figure 4 and Table 1) whose expression changes differed in the MPK407 strain from the wild-type cultured with uracil supplementation. The most abundant identifiable functional category encoded by this group of genes appears to involve chemotaxis (*vng0942G*, *vng0974G*, *vng0976G *and *vng1607G*). Moreover, numerous genes encoded on the replicon pNRC200 appear to be differentially regulated in the MPK407 strain. Given that many of the genes encode products of unknown function, we cannot offer a functional rationale for these observations other than to suggest caution when interpreting expression changes for these genes in responses of cells with the Δ*pyrF *genotype. A similar analysis did not reveal any alterations in gene expression changes due to the addition of uracil to the media.

**Table 1:** Genes identified as distinguishing the MPK407 knockout strain from wild-type by T-Test analysis

ORF Name	Replicon	Gene Symbol	Fold Change	Function (listed if known)
VNG0001H	Chromosome	*VNG0001H*	0.01977	

VNG0002G	Chromosome	*yvrO*	0.02768	ABC transporter, ATP-binding protein

VNG0003C	Chromosome	*VNG0003C*	0.02390	

VNG0035C	Chromosome	*VNG0035C*	0.0156	

VNG0058H	Chromosome	*VNG0058H*	87.10	

VNG0146H	Chromosome	*VNG0146H*	0.02169	

VNG0539C	Chromosome	*VNG0539C*	0.01066	

VNG0617H	Chromosome	*VNG0617H*	0.0326	

VNG0620G	Chromosome	*edp*	0.01955	Proteinase IV homolog

VNG0810H	Chromosome	*VNG0810H*	0.02972	

VNG0849C	Chromosome	*VNG0849C*	0.02645	

VNG0928G	Chromosome	*mak*	0.02170	MAPK-activated protein kinase

VNG0934H	Chromosome	*VNG0934H*	0.03188	

VNG0942G	Chromosome	*cheW2*	0.01911	Chemotaxis protein

VNG0943C	Chromosome	*VNG0943C*	0.01461	

VNG0958G	Chromosome	*htr15*	0.01418	Htr15 transducer

VNG0974G	Chromosome	*cheY*	0.03160	Chemotaxis protein

VNG0976G	Chromosome	*cheW1*	0.01584	Chemotaxis protein

VNG1276C	Chromosome	*VNG1276C*	34.86	

VNG1607G	Chromosome	*cheC2*	28.79	Chemotaxis protein

VNG2442H	Chromosome	*VNG2442H*	41.40	

VNG6155H	pNRC200	*VNG6155H*	90.14	

VNG6159H	pNRC200	*VNG6159H*	61.89	

VNG6332H	pNRC200	*VNG6332H*	66.12	

VNG6361G	pNRC200	*npa*	20.8	Neutral proteinase

VNG6368H	pNRC200	*VNG6368H*	36.26	

VNG6379C	pNRC200	*VNG6379C*	106.4	

VNG6385H	pNRC200	*VNG6385H*	42.94	

VNG6413H	pNRC200	*VNG6413H*	40.25	

VNG6420H	pNRC200	*VNG6420H*	24.48	

VNG6441H	pNRC200	*VNG6441H*	67.71	putative DNA-binding protein

### Physiology associated with exponential growth phase

In total 451 genes show decreased transcript abundance during stationary phase. Only 15, or 3% of all genes in this set are encoded on either of the two plasmids - pNRC100 and pNRC200, which together harbor 20% of all open reading frames in *H. salinarum *NRC-1. This suggests that plasmid encoded genes are disproportionately inactive during rapid growth.

Results of a search of the 451 genes against KEGG's pathway database are summarized in Additional file 6: Additional tables S3 and S4. Functionally over-represented processes that are mostly active during exponential phase include, not surprisingly, transcription, translation, and numerous genes associated with oxidative phosphorylation including the TCA cycle. The completeness of many of these functional sets in this data set is high. This is also illustrated by genes encoding various subunits of several ABC transporters, most notably iron transport. In addition, the transition to stationary phase was associated with the decreased transcript abundance of genes that encode biogenesis of cofactors, such as oxygen dependent cobalamin biosynthesis (the pathway leading from uroporphyrinogen III to cobyrinic acid).

### Physiology associated with stationary growth phase

As the culture enters stationary phase, transcripts for 772 genes are significantly increased in abundance. It is intriguing that while only 3% (15 genes) of all plasmid-encoded genes increased in abundance during logarithmic phase, 28% (151 genes) increased in abundance at stationary phase. These observations show that functions of genes encoded on the plasmids are disproportionally associated with stationary phase physiology (p = 0.004).

We reconstructed the stationary phase physiology of *H. salinarum *NRC-1 by searching the 772 genes against KEGG's pathway database (Additional file 6; Additional tables S5 and S6). The largest functional category among genes showing increased transcript abundance in stationary phase are those encoding ABC transporter family of proteins. This includes genes encoding complexes involved in phosphate transport, oligo and dipeptide transport, glycerol-3-phosphate transport, manganese transport and branched chain amino acid transport. This is not entirely surprising as nutrient limitation is expected during stationary phase and this would likely induce the expression of nutrient scavenging systems. Together with exhausted nutrients, the drop in oxygen availability in stationary phase [13]explains the decreased transcript abundance of the TCA cycle and increased transcript abundance of genes encoding alternate oxygen-independent energy transduction processes including phototrophy, DMSO/TMAO respiration and arginine fermentation. Other genes worth noting are the increased transcript abundance of those encoding both gas vesicle gene clusters, numerous heat shock proteins, the high affinity cytochrome d oxidase chains, an NADH dehydrogenase/oxidoreductase-like protein, an anaerobic glycerol-3-phophate dehydrogenase, sensory rhodopsin I and its corresponding signal transducer *Htr1*.

Finally, the simultaneous increase in transcript abundance during stationary phase of genes encoding tryptophan breakdown (by tryptophanase - *vng2373G*) and biosynthesis (from precursors chorismate and PRPP) is worth noting. Since serine is required as one of the final building blocks of tryptophan biosynthesis, one possibility is that this pathway could be operated in conjunction with a serine deaminase (*vng2100G*) to maximize the breakdown of serine into pyruvate.

### Transient physiologies

It is clear from the analysis thus far that two distinct physiologies characterize the exponential and stationary phases of growth. However, we also saw evidence that the transition to stationary phase is not entirely biphasic and that independently regulated processes occur in a temporal manner throughout growth.

#### Genes with transiently decreased transcript levels during exponential growth

Transcripts for arginine import and fermentation genes, *vng6313G *(*arcD*), *vng6315G *(*arcB*), *vng6316G *(*arcC*) and *vng6317G *(*arcA*) decrease in abundance transiently during exponential phase, relative to levels in early growth and stationary phases. These genes are involved in the fermentation of arginine as an alternate form of energy production under anaerobic environmental conditions. Therefore, while the increased transcript abundance of these genes during stationary phase was to be expected, their relatively high transcript levels in early exponential growth was surprising. However, experiments tracking amino acid consumption in defined media during growth of *Halobacterium salinarum sp. *R1 have shown cells apparently consume available arginine very quickly during early phases of growth [32]. An interpretation that is consistent with these two independent observations might be that arginine is being used early in growth to synthesize carbamoyl phosphate to fuel anabolic pathways that include pyrimidine biosynthesis. Finally, the simultaneous increase in transcript levels of arginine fermentation and synthesis genes *vng2436G *(*argH*) and *vng2437G *(*argG*) in the stationary phase might feed additional fumarate into the reductive carboxylate cycle.

#### Genes with transcripts that are transiently elevated during exponential growth

Transcripts for ten genes are transiently elevated during exponential growth relative to stationary phase and early exponential (Table 2). Among these, several (*vng0414G, vng0864G, vng0876G, vng1395G*) encode enzymes for the conversion of 5-phosphoribosylamine to 2-methyl-4-amino-5-hydroxymethylpyrimidine diphosphate, a direct precursor to thiamin phosphate, and as such, link purine metabolism with thiamin metabolism. Notwithstanding the caveat that some enzymes of thiamin biosynthesis in *H. salinarum *NRC-1 are yet to be discovered, this may provide thiamine based cofactors for enzymes.

**Table 2:** Genes whose transcript abundance are transiently increased during exponential growth

ORF Name	Replicon	Gene Symbol	Function (listed if known)
VN G0 4 1 4 G	Chromosome	*purH*	Phosphoribosylaminoimidazole - succinocarboxamide formyltransferase

VNG0715G	Chromosome	*thiC*	Thiamine biosynthesis protein

VNG0864G	Chromosome	*purL*	Phosphoribosylformylglycinamidine synthase II

VNG0876G	Chromosome	*purM*	Phosphoribosylformylglycinamidine cycloligase

VNG1305G	Chromosome	*purD*	Phosphoribosylglycinamide synthetase

VNG1814G	Chromosome	*c a rB*	Carbamoyl phosphate synthase large chain

VNG1940H	Chromosome	*VNG1940H*	

VNG1944C	Chromosome	*VNG1944C*	Phosphoribosylformylglycinamidine (FGAM) synthase

VNG2604Gm	Chromosome	*thi1*	Putative thiazole biosynthetic enzyme

VNG2606G	Chromosome	*thiD*	Hydroxymethylpyrimidine phosphate kinase

#### Genes with transcripts that are transiently increased in the transition to stationary phase

The transcript for eighteen genes show transient increased in abundance during the transition from exponential growth into the initial stages of stationary phase (Table 3). The functional theme within this group of genes is redox chemistry. There are two putative halocyanin precursors (*vng2196G, vng1188G*), one putative quinole oxidase (*vng2413H*), a putative Cu containing reductase (*vng1187G*) and two alternative cytochrome C oxidase subunits (*vng2193Gm, vng2195G*). This suggests that the organism may be retooling some of its capacity for redox chemistry at the end of exponential growth. It is known that other organisms will exchange cytochrome C oxidase subunits to remain more metabolically versatile in response to changing redox state [33]. The other 12 genes include 6 putative genes which lack annotation (*vng0602C, vng1182H, vng1486H, vng2197H, vng2199H and vng2412H*), a putative putative haloacid dehalogenase-like hydrolase (*vng0719G*), a putative iron-sulfur cluster containing protein (*vng1184Gm*), a putative universal stress protein (*vng1898C*), a putative phosphoribosyl transferase (*vng1912G*), a heavy-metal transporting CPx-type ATPase (*vng2201G*) and an putative aminopeptidase (*vng2546G*).

**Table 3:** Genes whose transcript abundance are transiently decreased during the transition from exponential growth into the initial stages of stationary phase

ORF Name	Replicon	Gene Symbol	Function (listed if known)
VNG0602C	Chromosome	*VNG0602C*	

VNG0719G	Chromosome	*araL*	putative haloacid dehalogenase-like hydrolase

VNG1182H	Chromosome	*VNG1182H*	

VNG1184Gm	Chromosome	*nirJ*	predicted Fe-S cluster protein involved in cofactor biosynthesis.

VNG1187G	Chromosome	*nirK*	putative Cu-containing nitrite reductase.

VNG1188G	Chromosome	*hcpD*	Halocyanin precursor-like

VNG1486H	Chromosome	*VNG1486H*	

VNG1898C	Chromosome	*VNG1898 C*	Universal stress protein UspA and related nucleotide-binding proteins PF00582.

VNG1912G	Chromosome	*trpD2*	Phosphoribosyl transferase

VNG2193Gm	Chromosome	*coxA1*	Cytochrome c oxidase subunit I

VNG2195G	Chromosome	*coxB2*	Cytochrome c oxidase subunit II

VNG2196G	Chromosome	*hcpB*	Halocyanin precursor-like

VNG2197H	Chromosome	*VNG2197H*	

VNG2199H	Chromosome	*VNG2199H*	

VNG2201G	Chromosome	*cpx*	Heavy-metal transporting CPx-type ATPase

VNG2412H	Chromosome	*VNG2412H*	

VNG2413H	Chromosome	*VNG2413H*	putative quinole terminal oxidase

VNG2546G	Chromosome	*pepB3*	Aminopeptidase homolog

#### Expression profiles of transcription factors during growth of H. salinarum *NRC-1*

The scale of the physiological shift during transition of actively dividing cells to stationary phase suggests that this global phenomenon must to some degree be mediated by the activity of general transcription factors (GTFs), which are known to transcriptionally segregate large groups of functionally-related genes in *H. salinarum *NRC-1 [16]. This view is supported by significant changes in levels for 9 of the 13 GTF transcripts in this experiment. Surprisingly, transcript levels of only two, *TFBf *and *TBPe*, were significantly higher during exponential growth. Together with analysis of genome-wide distribution of their transcription factor binding sites (TFBSs) generated in a previous study [16], their statistically inferred regulatory function within an integrated model of global gene regulation [4,34], and failed attempts to knockout these GTFs, this contributes additional evidence for a primary role for *TFBf *and *TBPe *during active growth. The primary role of *TBPe *in exponential growth was also recently supported by qRT-PCR experiments that quantified the expression of *TBPs A, B, D *and *E *in exponential phase under both anaerobic and aerobic conditions [35]. These experiments showed that the *TBPe *transcript is nearly 8-fold more abundant in exponentially growing cells than any other detectable *TBP *transcript.

Transcripts for seven of the 13 GTFs (*TFBa, TFBb, TFBd, TFBe, TFBg, TBPf*) showed increased abundance during stationary phase of growth. This suggested that the GTF regulatory network of stationary phase might be more complex than the network operating during exponential growth. It is tempting to speculate that gene regulation is of greater importance to optimize energy utilization when resources are limited and that this might consequently require a more complex regulatory network to regulate appropriately. If this is indeed the case, though, one might also suspect that deletion or over-expression of these GTFs would also result in significant perturbations to growth behavior. However, results from a previous study [16] showed that the genetic perturbation in most of these GTFs did not have large consequences on growth or response characteristics [16]. This may be an outcome of a high degree of redundancy in the GTF regulatory network that might buffer inadvertent loss of their functions to spontaneous mutations. Inadequate sampling of environmental condition space may contribute to the lack of obvious phenotypes in these mutants.

In addition, 8 known or putative bacterial-like transcriptional regulators show decreased abundance in stationary phase while 27 show increased abundance in stationary phase. Given our estimate of ~100 transcriptional regulators (not including GTFs) in *H. salinarum *NRC-1 it is noteworthy that more than one third of these additional regulators might also mediate growth-related physiological changes.

### Evidence that simple growth studies can inform on numerous aspects of cellular physiology

Using three observations we illustrate how the study of growth can provide evidence and generate testable hypotheses for a variety of research areas in cellular physiology. Specifically we highlight (a) the integration of metabolomic data with gene expression data to generate hypotheses regarding growth-associated nutrient fluxes (b) use of growth-associated transcriptional patterns for distinguishing among paralogous functions and (c) analysis of perturbed culture characteristics in a GTF mutant to explain transcriptional control of cellular morphology during growth.

#### Metabolism: Coordinated analysis of transcript and metabolite data

Metabolite profiling in halophilic archaea is complicated by the incompatibility of residual salt in the extraction buffer that interferes with most MS-based profiling technologies. We attempted some preliminary intracellular metabolite extraction and analysis over different growth phases to see whether or not growth studies could justify the effort and resources required for streamlining and integrating metabolomics-based approaches with gene expression profiling for reconstructing cellular responses to environmental changes.

Cells were processed and metabolites were extracted and analyzed as described in Methods. Among the final set of identifiable metabolites whose abundance changed significantly during growth, we noticed patterns of changes in metabolite abundance that mirrored the patterns of gene expression we described earlier (Additional file 7). Following this analysis, the 10 most significantly differentially regulated metabolites were targeted for further characterization using MS/MS and by comparison to chemical standards. Of these 10, 4 compounds were positively identified based on accurate mass, retention time and MS/MS pattern (citrulline, phenylalanine, riboflavin and 5'-deoxyadenosine). 5'-deoxyadenosine and riboflavin each showed increases in abundance over growth while phenylalanine showed a decrease in abundance over the growth curve (Additional file 8).

**Figure 5:**
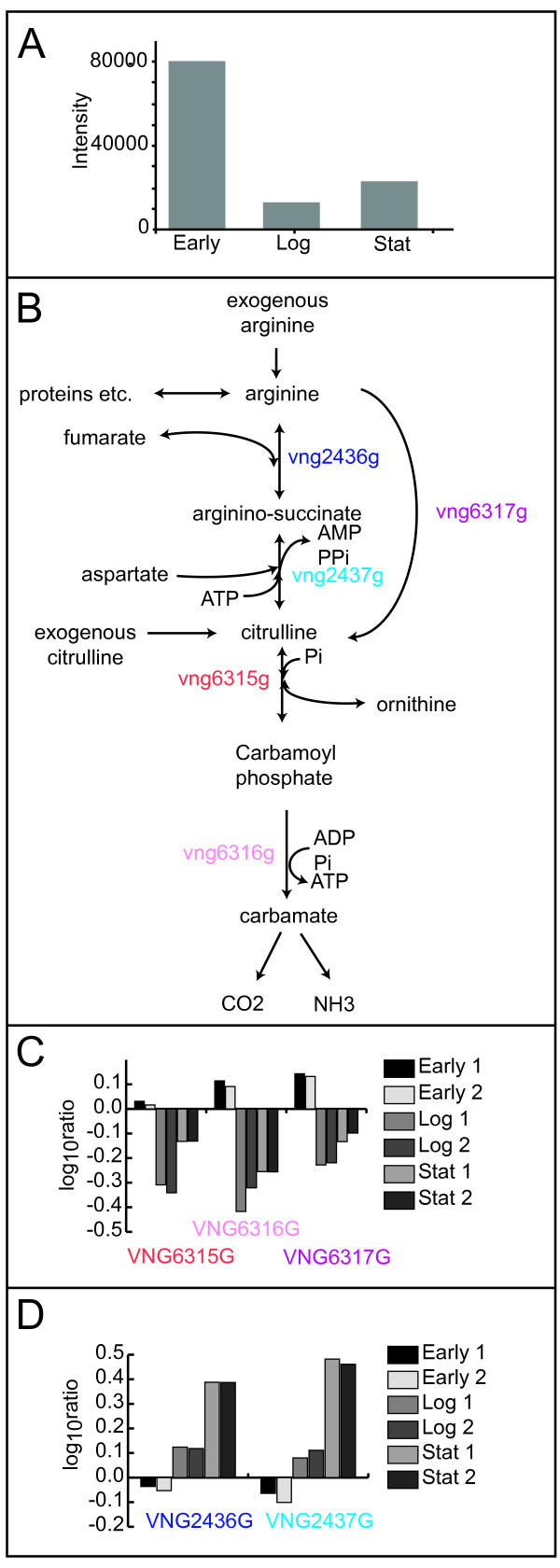
**The behavior of citrulline during growth**. Changes in metabolite concentration were measured throughout the growth curve of *H. salinarum *NRC-1. Some metabolites showed coordinated behavior with the gene expression of enzymes that catalyze their synthesis or degradation. (A) The metabolic pathways involved in citrulline degradation and biosynthesis in *H. salinarum *NRC-1. Gene names encoding enzymes catalyzing the illustrated metabolic reactions are shown near the arrows. (B) Abundance profile for citrulline across the three growth phases, early growth, exponential growth and stationary phase. (C) Gene expression profiles for genes involved in arginine fermentation, *vng6315G, vng6316G *and *vng6317G*. Note the similar patterns in the changes of abundance of these three genes and the abundance of citrulline. (D) Gene expression profiles for *vng2436G *and *vng2437G*. The enzymes encoded by these genes catalyze the innerconversion of cirtulline to arginine. Note the differential regulation of these genes with those highlighted in panel C. While both pathways are active during stationary phase, the abundance of citrulline is nevertheless best predicted by the activity of *vng6315G*, *vng6316G *and *vng6317G*.

However, the most conspicuous pattern of change is in the abundance of citrulline - high in early growth, low in exponential growth and increasing in stationary phase (Figure 5A). We found this intriguing because citrulline is an intermediate in the fermentation of arginine (Figure 5B) and the gene expression of the enzymes that catalyze this pathway (*vng6315G - arcB, vng6316G - arcC, vng6317G - arcA*) show a similar pattern of relative abundance in across growth (Figure 5C). Meanwhile transcripts encoding enzymes catalyzing an alternate pathway between citrulline and arginine (*vng2436G - argH, vng2437G - argG*) show steady increase in abundance over growth (Figure 5D). This data suggests that the flux of metabolites through the arginine fermentation pathway, the former path, may be regulated by the transcription of the genes responsible for the catalysis rather than through an enzyme-level, substrate-level or translational-level process.

Although preliminary, this example nevertheless highlights the power of coordinated analysis of transcript and metabolite data over growth conditions.

#### Annotation: Functional segregation of orthologous genes

**Figure 6:**
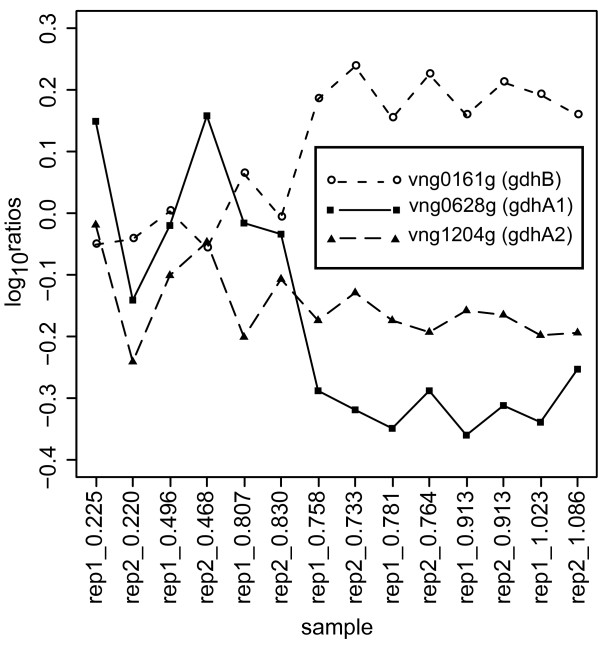
**Expression profiles for glutamate dehydrogenase homologs**. The gene expression profiles for the three glutamate dehydrogenase homologs are plotted for one of the experimental data sets. Two of the three homologs show highly differential expression patterns in the transition to stationary phase while the third homolog shows little change in expression throughout the experiment.

An interesting example of how this simple study has led to a potential hypothesis for functional annotation involves the analysis of three genes annotated as glutamate dehydrogenases (GDH). Expression profiles for each of the GDH homologs, *vng0161G *(*gdhB*), *vng0628G *(*gdhA1*) and *vng1204G *(*gdhA2*) are shown in Figure 6. Notably, *gdhB *and *gdhA1 *have anti-correlated expression patterns upon transition to stationary phase. Whereas transcript levels for *gdhB *increase upon entering stationary phase, the transcript level for *gdhA1 *decreases. The transcript levels for *gdhA2 *do not show significant change in this experiment

*GdhA1 *has been experimentally identified as an NADPH dependent enzyme while the cofactor preference of the other two enzymes remains unknown [36]. A complicating factor impeding the functional annotation of the remaining GDHases is the fact that no known sequence relationship can distinguish between NAD^+ ^and NADPH-associated GDH enzymes [37]. However, in yeast, the NAD^+ ^and NADPH-associated GDH enzymes are reciprocally regulated as a function of changes in various factors including ammonium ion levels [38]. If the genome of *H. salinarum *NRC-1 encodes, like yeast, multiple genes that are true GDHases, a direct comparison to yeast would suggest that, the expression anti-correlated *gdhB *and *gdhA1 *genes could, respectively, encode an NAD^+ ^dependent GDH that would drive the oxidation of glutamate to oxoglutarate and an NADPH dependent enzyme catalyzing the reverse reaction. Meanwhile, the role of the third annotated GDH gene, *gdhA2*, remains less clear. This hypothesis presumes that each gene annotated as a GDH actually function as GDHs *in vivo*. Further biochemical characterisation will be necessary to confirm or refute this hypothesis. Our interpretation of the data nevertheless represents a reasonable and testable hypothesis that was generated simply by inspection of transcriptional growth data.

#### Physiology and phenotype: Analysis of a morphological phenotype in a GTF knock-out strain

**Figure 7:**
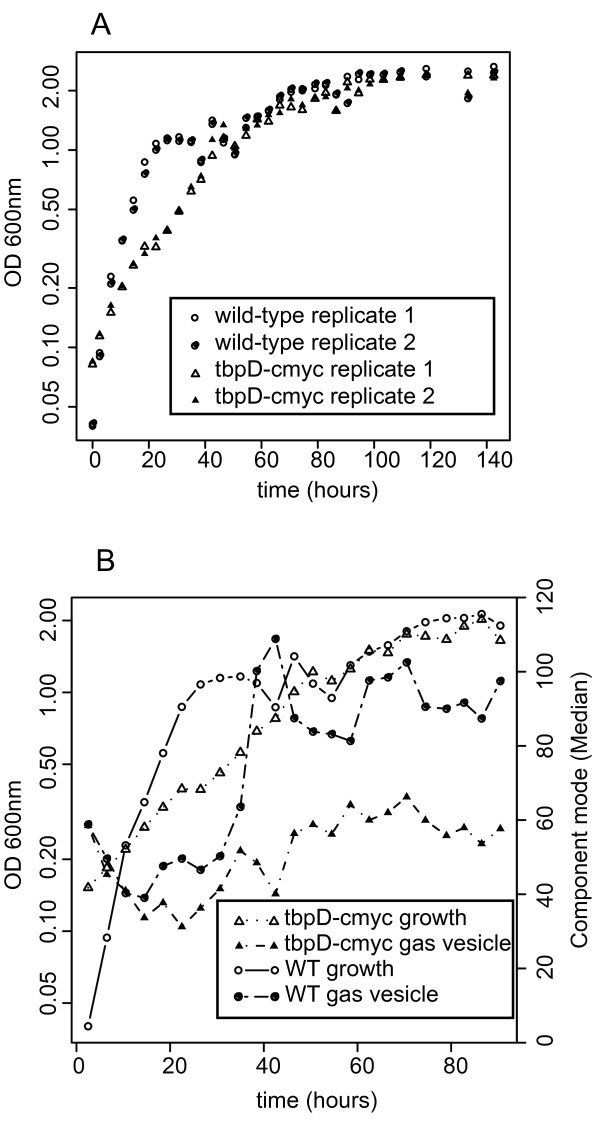
**Growth and gas vesicle production of *H. salinarum *NRC-1 and the *tbpD *non-native expression mutant strains**. (A) A comparison of growth curves for wild-type H. salinarum NRC-1 and the nonnative *tbpD *expression strain. Optical density measured at 600 nm (OD_600_) is reported for two replicate flask growth cultures for each strain. Severe growth retardation is evident in the nonnative *tbpD *expression strain. (B) Growth versus gas vesicle production in wild-type cells and the nonnative *tbpD *expression mutant is shown as measured by OD_600 _and median pixel intensity for the cellular population, respectively. An increase in gas vesicle production is noted in wild-type cells that is absent in the *tbpD *mutant.

Microscopic inspection of all GTF perturbed strains identified a marked phenotype associated with non-native plasmid-borne expression of *tbpD*. Along with a slow doubling time, this strain was defective in gas vesicle production (Figure 7) and had perturbed morphology. Growth retardation and perturbed gas vesicle production are also consistent with growth retardation and reduced expression of the gas vesicle operon in *tbpD *knockout strains [39] suggesting that *tbpD *is indeed involved in determining both of these phenotypes. Due to the multiplicity of aberrant phenotypes this strain was subsequently selected for further phenotypic analysis to evaluate whether changes in gene expression could be related to the altered phenotype.

**Figure 8:**
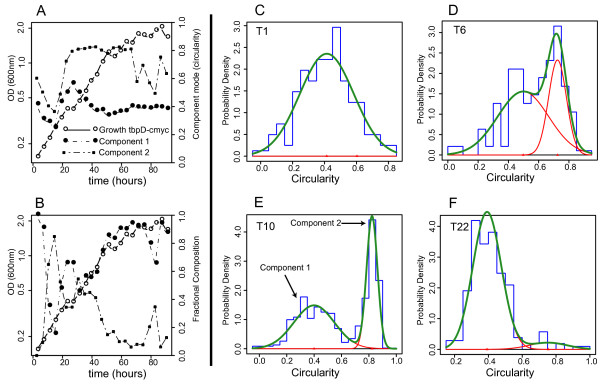
**Analysis of the growth morphologic transition that occurs during the growth of nonnative tbpD expression**. Mixture model analysis of the morphology of the cellular population during growth. (A) Growth of the nonnative *tbpD *expression mutant as measured by optical density measured at 600 nm (OD_600_) is co-plotted with the mean circularity of cells for the two components. Component 1 is associated with rod cells, component 2 is associated with spherical cells. At time point 6 there is an overall increase in circularity. (B) The fractional composition of each mixture component. The symbols are the same as those used in panel A. A reduction in circularity for component 2 during exponential growth, coupled with a concomitant decrease in component 1 circularity indicates an increase in rod-like cells in exponential growth. During the "flattening" phase, the fractional composition of the circular component 2 is near 40% of all cells but the mean circularity of this component increases dramatically indicating that a large portion of the cellular population is circular during this phase. (C) A plot of mixture model components 1 and 2 at time point 1 in the growth curve. Red lines indicate the fit curves for each component in the mixture. Green curves indicate the additive fit to the frequency distribution shown as a histogram. (D) Mixture model for time point 6, the beginning of the flattening phase. (E) Mixture model for time point 10, just before the sharp decrease in abundance of component 2. (F) Mixture model for time point 22, showing a return to rod shaped cells with a minor circular component.

Here we highlight a *tbpD*-associated phenotype related to cell morphology that occurs during an apparent "flattening" or transition in the growth of the non-native expressing *tbpD *cells. Interestingly this "flattening" coincides with the transition to stationary phase for wild-type cells. We observed that a large fraction of the cells with non-native *tbpD *expression undergo a shift from rods to cocci during the "flattening" phase of growth (Figure 8). Following the flattening phase, the population generally returns to rod shaped cells. This brief, but dynamic transition in cellular morphology has to our knowledge not been noted in *H. salinarum *NRC-1 physiology.

To interrogate this morphological phenotype we analyzed gene expression of patterns through growth in the *tbpD *non-native expression strain. Few distinctive changes in expression patterns are evident in this data with the clear exception of the decreased transcript abundance of gas vesicle genes and the citric acid cycle catalyst aconitase (*vng2574G*). The gas vesicle minus phenotype observed during imaging is thus corroborated by the perturbed expression of gas vesicle genes, specifically gas vesicle cluster 1. The perturbed expression of aconitase may partially explain both the noted slow growth and the circular morphology. While it is reasonable to assume that reducing the expression of a TCA cycle intermediate could slow metabolic activity sufficiently to reduce growth rate, citrate accumulation is also thought to be a principle culprit for growth retardation in aconitase deficient mutants [40-43]. Meanwhile the aberrant cell morphology is similar to morphological perturbations noted for aconitase inactivation mutants in *Streptomyces coelicolor *[44], *Streptomyces viridochromogenes *[45], *Bacillus subtilis *[46] and *Staphylococcus aureus *[47] suggesting a common, if not yet understood, role for aconitase in the maintenance of cellular morphology in bacteria and archaea.

#### Comparison with other studies

One of the most surprising element of this study is the degree of changes in transcript abundance detected across the growth curve (at least 63% of all possible transcripts). The closest comparable study in *H. salinarum *NRC-1 is that of Lange et al. [19] who reported 17% differentially abundant transcripts. Despite the large difference in number of differentially abundant transcripts, the functional themes that distinguish exponential and stationary phases in Lange et al and this study are similar. For instance genes associated with, transcription, translation, oxidative phosphorylation and the TCA cycle are both increased in abundance during exponential growth. Meanwhile, similar sets of ABC transporters, alkaline phosphatase and numerous genes of unknown function commonly define the stationary phase in both experiments. Interestingly, while we report increased abundance of bacteriorhodopsin, halorhodopsin and genes involved in arginine metabolism/fermentation in stationary phase, these are not seen as differentially abundant in Lange et al. These processes have conventionally been associated with stationary phase-like physiology [13,28]. We note, however, that Lange et al. used a more stringent cutoff for differential abundance than that used in this study and this, in addition to lower sampling frequency, may partially explain the difference between the two studies.

A critical component of Lange et al's. study is that in addition to changes in transcript abundance they also measured translational efficiencies between exponential and stationary growth. This provides the opportunity for some interesting comparisons. For instance, one notable difference between Lange et al. and this study is the near absence of differentially abundant transcription factors, particularly general transcription factors which contrasts with this study. Interestingly, however, Lange et al. do note significant translational regulation of numerous general transcription factors in exponential growth. This suggests that some of the transcription factors, like *tfbE *(and other genes alike), while transcriptionally increased in stationary phase may nevertheless have significant function during exponential growth.

It is, finally, worth comparing growth or growth-related studies in other organisms to the currently presented data. One early study in *Escherichia. coli *reported 27% of all transcripts showing differential abundance between stationary and exponential growth phases [48]. In methanogenic archaea, gene expression studies experiments of nutrient limitation, which may approximate in some respects the nutrient depletion present in stationary phase, also show significantly fewer transcriptional changes that those noted herein [49-51].

#### Potential implications

The set of experiments presented herein, and the demonstrated extent to which transcriptional changes across a complete genome can be observed, serve to highlight the often forgotten potential for information inherent in relatively simple growth studies. A general approach to the systems-level study of any newly or poorly characterized organism could begin by characterizing changes in gene expression over a relatively simple growth experiment. For example, we have already applied this principle to capture the structure of the transcriptome (transcription start sites (TSSs), transcription termination sites (TTSs), non-coding RNAs (ncRNAs) etc.). By sampling at 13 distinct points covering the various phases of growth we were able to reveal fine mechanistic features of transcriptional control (alternate transcription start sites, imprecise termination events, conditional promoters inside genes and operons, potential regulation by ncRNAs etc.) associated with most genes of *H. salinarum *NRC-1 [18]. In yet another study, knowledge of physiological states associated with growth has helped us to suggest possible reasons why cells at the cusp of transition from exponential to stationary phase are most responsive to entrainment to diurnal light-dark cycles [52]. We rationalize that the semi-starved state in the transition between growth and stationary may indeed be more closely related to the natural environmental conditions to which the sensory and regulatory systems in *H. salinarum *have evolved. Therefore, it is reasonable to consider the proposition that other experiments conducted in this growth regime might be most informative regarding the physiology of the organism.

In the context of systems-level reconstructions of physiology and gene regulation, a simple experiment that can stimulate the changes in expression for over 60-80% of all genes without any externally administered perturbation(s) (Tables 4 and 5) can be potentially very useful. Current best efforts in system-level reconstructions of gene regulatory networks have thus far required the integration of over 200 microarray experiments interrogating cellular responses to individually administered perturbations [4], to achieve broad coverage of genes. This study suggests that including growth studies early during data collection may prove useful in limiting the overall number of experiments that may be necessary to reconstruct models of gene regulatory networks.

**Table 4:** Number of genes meeting lambda threshold cutoffs over 4 consecutive conditions

Filtering threshold (λ) over 4 consecutive conditions	Genes meeting filter	Fraction of all genes
15	1518	0.63

12	1678	0.7

10	1799	0.75

7	1994	0.83

**Table 5:** Number of genes meeting lambda threshold >15 cutoff over 4 or fewer consecutive conditions

Filtering threshold (λ > 15) over X conditions	Genes meeting filter	Fraction of all genes
X = 4	1518	0.63

X = 3	1700	0.71

X = 2	2014	0.84

X = 1	2109	0.88

Additionally, one of the overarching goals in systems biology is not only to reconstruct a detailed understanding of cellular physiology/networks but to also place this understanding into an ecological context [7]. Cells have, after all, evolved their regulatory network structures in response to environmental pressures. Indeed, the reason so many genes change in the simple batch experiment presented in this manuscript is that growth exposes the cells to an ever fluctuating set of environmental cues. The consumption of oxygen and nutrients during growth forces the cell to retool its metabolism from an oxic mode to an anoxic mode, to successively use alternate nutrient sources and to fight stress arising from accumulation of toxic byproducts. These combined stresses are likely among the key triggers for the massive shift in physiology we've reported above.

Therefore, in the context of the systems-level study, it would also be highly appropriate to (a) provide mechanisms to control environmental factors in the experiment (e.g. by using bioreactors rather than flasks for culturing) and (b) make quantitative measurements on the levels of as many environmental factors as possible during the experiment. The latter could then be queried for their ability to predictably inform on the noted physiological changes. The critical, and yet undetermined, element in this scenario would be to use the bioreactor (or similar device) in a manner that would only control environmental factors sufficiently to allow for reproducibility in the aforementioned measurements while still allowing numerous environmental factors to change, thereby inducing differential gene expression.

This last point is admittedly controversial because it challenges what have been very informative protocols established during years of single-perturbation studies. These single perturbation experiments typically compare the results from two experiments, control and perturbation, in which all environmental factors, except the perturbation, are held constant. What we are proposing above is effectively a multi-parameter perturbation experiment in which numerous environmental factors are simultaneously perturbed. Given both our ever-improving ability to make low cost measurements of numerous metabolites, likely the primary environmental factors changing during growth, and our improving ability to learn functional relationships from multi-parameter biological data sets, our proposed approach seems like it might be a reasonable approach. The potential for information rich data sets illustrated in this manuscript do suggest that it is worthwhile to pursue.

## Conclusions

Our analysis of global transcriptional changes that occur in *H. salinarum *NRC-1 during growth in simple batch culture, show the physiological changes encoded by 1,518, or 63% of the approximately 2400 putatively identified non-redundant genes. The exponential phase is characterized by genes associated with transcription, translation, and numerous genes associated with oxidative phosphorylation including the TCA cycle. Meanwhile, stationary phase seems associated with increased abundance of transcripts for genes encoding protein complexes involved in phosphate transport, oligo and dipeptide transport, glycerol-3-phosphate transport, manganese transport, branched chain amino acid transport and those encoding alternate oxygen-independent energy transduction processes including phototrophy, DMSO/TMAO respiration and arginine fermentation. The changes in transcript abundance noted in the stationary phase are also disproportionately associated with genes encoded on the two plasmids, pNRC100 and pNRC200, for whom functions are not known. In addition, while the majority of changes in transcript abundance are associated with classic exponential and stationary phases, the sampling frequency of this experiment has demonstrated that transient changes in transcript abundance occur during exponential growth and in the transitions between exponential and stationary phases which include thiamine biosynthesis and redox enzymes.

This study also demonstrates how the analysis of transcriptome growth data in the context of other data types, including metabolomic data and gene perturbation can lead to novel biological insight. Finally, we suggest that one of the potential significant implications of this study is that the integration of growth as a parameter in traditional perturbation studies may prove to be useful in minimizing the number of experiments necessary to observe a near comprehensive set of functional association patterns between different genomically-encoded elements. This could help to drive further development of systems-level analysis in numerous non-model organisms whose genomes are being sequenced at an ever increasing rate.

## Methods

### Strains and culturing microarray data collection and analysis

*H. salinarum sp. *NRC-1 is the wild-type strain [53]. The *TBPd*-cmyc non-native expression strain was constructed as previously described [16] by transforming the wild-type strain with a pMTFcmyc vector into which the coding sequence for *TBPd *has been cloned. The construction of the MPK407 strain (the *pyrf *mutant of *H. salinarum *NRC-1) was described previously [30]. Culturing of all strains was done at 37°C in liquid nutrient-rich Complete Medium (CM). The composition of CM is as follows: 250 g/L NaCl, 20 g/L MgSO_4_•7H_2_O, 3 g/L Sodium Citrate, 2 g/L KCl and 10 g/L Peptone (Oxoid, United Kingdom) made with distilled water. Cultures were inoculated to a starting optical density at 600 nm of 0.02 with starter culture of optical density at 600 nm of 0.5 which was derived from a single colony. Cultures were grown in unbaffled flasks in which 40% of the flask volume is occupied by the culture. Cultures were shaken at 220 RPM illuminated at ~20 μmol/m2/sec (standard illumination in Innova9400 incubators (New Brunswick)). Duplicate cultures were grown and samples were harvested for RNA extraction at points indicated in Figure 2. For strains harboring pMTFcmyc plasmids, 20 μg/ml mevinolin (final concentration) was added to the culture medium. When indicated, 50 μg/ml uracil has been added to the media.

### Microarray analysis

Unique 70-mer oligonucleotides spanning 2400 of the putatively identified non-redundant genes in *H. salinarum *NRC-1 genome were bought from QIAGEN (Qiagen, Valencia, CA). Microarray fabrication, RNA preparation, labeling with Alexa 594, and Alexa 660 dyes (Invitrogen, Carlsbad, CA), hybridization, and washing have been described previously [54]. Each comparison was performed twice with total RNA from two independently processed cultures. A reversal in the labeling dyes (dye-flip) was included to rule out bias in dye incorporation. Raw data was processed, log10ratios were estimated, and the significance of change was determined by the maximum likelihood method [29].

Microarray data was analyzed using Gaggle [55], TM4 [31], and the R statistical package (version 2.7.0; R Foundation for Statistical Computing http://www.R-project.org). Data were row normalized and filtered according to the following criteria: genes must have lambda >15 for 4 consecutive conditions to be considered significant. A total of 1518 genes met this criteria. Fold change was calculated by taking the ratio between the average non-logged ratio for the last four samples (replicates included) taken in the growth curve to the average of the first four samples taken in the growth curve. A t-test, computed on logged data, was also used on the same selected sets to (first and last four data points for each strain) to ask whether the changes in expression were statistically significant given an overall p-value threshold = 0.05 and enforcing a false discovery rate of 0.05 or less.

### Cell morphology and gas vesicle quantitation

Wild type and *tbpD*cmyc-expressing cells were grown as indicated above. Cell aliquots were harvested at each data point in Figure 2. Cells were diluted to an optical density at 600 nm = 0.1 for all samples and then were subsequently fixed by the addition of 0.25% formaldehyde (final concentration). Cells were then imaged by phase contrast microscopy and digital photos were taken for numerous fields for each sample. Images were then processed with ImageJ (version 1.39u; National Institutes of Health, USA http://rsb.info.nih.gov/ij/) to extract individual cells and to quantify cellular length, width, circularity and average pixel intensity. Average values from a minimum of 50 individual cells were used to quantify each datapoint. Matlab (Mathworks, Natick, MA) scripts were used to process single frame ImageJ output into single files. R scripts were written to generate mixture models for cellular morphology using the mixdist R package (version 0.5-1; McMaster University http://www.math.mcmaster.ca/peter/mix/mix.html). Plots were generated with R.

### Chromatography and Mass Spectrometry

Approximately 10^8 ^cells were harvested by centrifugation during early growth, exponential growth and stationary phase and washed with 2 mL basal salts to remove contamination from the media. Metabolites were extracted from cell pellets with 100 μl of 100% cold methanol. Samples were centrifuged at top speed in a microfuge to remove unsolubilized material. The supernatant was dried by SpeedVac at cold temperature. 5 μL of sample reconstituted in 0.1% formic acid was injected for each run using the microwell autosampler of an Agilent 1200 HPLC system (Agilent, Santa Clara, CA). Reverse phase chromatography was performed using a Waters Symmetry C18 (3.5 μm, 2.1 × 100 mm) column (Waters Corporation, Milford, MA) at a flow rate of 250 μL/min. Buffer A water with 0.1% formic acid (v/v) and buffer B was acetonitrile with 0.1% formic acid. The column was equilibrated at 100% A, then ramped to 5% B over 5 minutes. Then the gradient elution was used over the next 30 minutes to 95% B and maintained for 5 minutes. A m/z scan range of 85-2000 was used on an Agilent ESI-q-TOF mass spectrometer in positive ion mode. The capillary was kept at 3500 V and the fragmentor was set to 175 V for the analysis. Collision energy for each metabolite was optimized for maximum fragmentation efficiency, and identical collision energies were used for each chemical standard and the endogenous metabolite for identification.

The LC-MS runs performed on the Agilent ESI-TOF mass spectrometer were used for a comparative analysis using XCMS [56] to identify the most significant features after performing a non-linear alignment of the chromatograms and using a Student's t-test. Ten metabolites with significantly changed abundance were targeted for identification using MS/MS and comparison with chemical standards. The workflow for mass spectrometry based identification consisted of first, using the accurate mass (<5 ppm) and isotopic pattern of the small molecule metabolites to narrow the molecular formula possibilities. Then, a search is conducted against the available metabolite databases: KEGG http://www.genome.jp/kegg/, METLIN http://metlin.scripps.edu/, Lipidmaps http://www.lipidmaps.org/ and HMP http://www.hmdb.ca/ to look for known metabolites which correspond to the ions of interest. High purity chemical standards of hypothesized structures of ions of interest were bought (Sigma-Aldrich, St. Louis, MO) and identification was performed by a precise match of MS/MS pattern and chromatographic retention time under identical experimental conditions on an Agilent ESI-q-TOF mass spectrometer.

Individual metabolites were identified with high statistical significance (p-value < 0.001) and were further filtered based on a minimum 3-fold change between early and stationary phase abundance. Finally, manual inspection to eliminate noisy profiles reduced the final set of metabolites to nearly 150 that were searched against the METLIN database of characterized metabolites http://metlin.scripps.edu/.

### Data Deposition

Microarray data for this study can be accessed from the Gene Expression Omnibus http://www.ncbi.nlm.nih.gov/projects/geo with accession numbers: GSE14832, GSE14835 and GSE14836. Metabolomic data can be accessed via http://baliga.systemsbiology.net/.

## Authors' contributions

MTF conceived of the study, participated in the design, collected data and drafted the manuscript. WLP collected growth curve data, wrote image analysis scripts and helped draft the manuscript. FYL conducted growth studies on GTF perturbation strains. KW, TK and KM conducted growth experiments and helped draft the manuscript. MP and AK conducted gene expression analysis. DL conducted experiments characterizing the morphology of TBPd mutants. DJR wrote scripts to analyze functional overrepresentation in gene sets. LH, EK, TN, SAT and GS designed and conducted the metabolite analysis. NSB participated in the design of the study and in its coordination and helped draft the manuscript. All authors have read and approve of the final manuscript.

## Supplementary Material

Additional file 1 **Additional figure S1. Interrogation of the apparent second "growth" phase of *H. salinarum *NRC-1 in rich media**. (A) Growth of *H. salinarum *NRC-1 was tracked by optical density measurements at both 600 nm and 700 nm to determine the contribution of bacteriorhodopsin (a protein whose broad absorption peak centered at 568 nm can contribute to absorption at 600 nm but not at 700 nm) accumulation. The growth curve generated at 700 nm displays similar behavior to the curve generated from measurements taken at 600 nm. In particular, the apparent doubling that occurs during what appears to be stationary phase is apparent in both curves suggesting that this is not due to bacteriorhodopsin accumulation. This increase in optical density is most likely due to an increase in light scattering gas vesicles that are visibly released during this late phase of the growth experiment. (B) Phase-contrast visible light microscopy image of *H. salinarum *NRC-1 near the end of data collection for the data presented in Figure 1 main text. Three arrows point to examples of small bright bodies (presumably gas vesicles) that populate the field of view. This abundance of gas vesicles only becomes apparent after a significant decrease in CFU following stationary phase. Click here for file

Additional file 2 Potential causes for the apparent second growth phase in *H. salinarum NRC-1*. Click here for file

Additional file 3 **Additional figure S2. Heat maps of genes with significant up or down regulation during growth**. Samples from each individual experiments (e.g. MPK407, *H. salinarum NRC-1 and H. salinarum NRC-1 *+ uracil) are organized by increasing optical density moving from left to right. The black volume bar indicates increasing optical density. Genes have been hierarchically clustered to show potential subpatterns of expression. **(A) **A heatmap showing the changes in expression of 451 genes whose transcript abundance is decreased upon entry into stationary phase. **(B) **A heatmap showing the changes in expression of 772 genes whose transcript abundance is increased upon entry into stationary phase. Click here for file

Additional file 4 **Additional table S1 - Genes whose transcript abundance is decreased during the transition to stationary phase**. This table lists ORF name, gene symbol, an estimate fold change in expression between pre-stationary and stationary phase expression, an indicator of significance of change between pre-stationary and stationary phase expression values and the putative gene function (if known). Fold change was calculated by taking the ratio between the average non-logged ratio for the last four samples (replicates included) taken in the growth curve to the average of the first four samples taken in the growth curve. A t-test, computed on logged data, was also used on the same selected sets to (first and last four data points for each strain) to ask whether the changes in expression were statistically significant given an overall p-value threshold = 0.05 and enforcing a false discovery rate of 0.05 or less. 418 genes of the 451 in this clustering derived set were deemed to have significantly different expression levels using this criteria while the remaining 33 genes did not. Genes meeting this criteria are marked with a number one while those not meeting the criteria are marked with a zero. Manual inspection of expression profiles of genes not meeting the above criteria suggest that the t-test in this instance may be too conservative as many gene expression profiles deemed not significant show what seems to be clear decrease in signal between pre-stationary and stationary phases. Click here for file

Additional file 5 **Additional table S2 - Genes whose transcript abundance is increased during the transition to stationary phase**. This table lists ORF name, gene symbol, an estimate fold change in expression between pre-stationary and stationary phase expression, an indicator of significance of change between pre-stationary and stationary phase expression values and the putative gene function (if known). Fold change was calculated by taking the ratio between the average non-logged ratio for the last four samples (replicates included) taken in the growth curve to the average of the first four samples taken in the growth curve. A t-test, computed on logged data, was also used on the same selected sets to (first and last four data points for each strain) to ask whether the changes in expression were statistically significant given an overall p-value threshold = 0.05 and enforcing a false discovery rate of 0.05 or less. 713 genes of the 772 in this clustering derived set were deemed to have significantly different expression levels using this criteria while the remaining 59 genes did not. Genes meeting this criteria are marked with a number one while those not meeting the criteria are marked with a zero. Manual inspection of expression profiles of genes not meeting the above criteria suggest that the t-test in this instance may be too conservative as many gene expression profiles deemed not significant show what seems to be clear increase in signal between pre-stationary and stationary phases. Click here for file

Additional file 6 Functional KEGG pathway and ontology annotations for those genes reported in Additional tables S1 and S2. Functional assignments are as reported by KEGG, unmodified. Click here for file

Additional file 7 **Additional figure S3. Metabolites showing significant changes in abundance during growth**. Each panel show a bar chart illustrating the changes in abundance for 51 detected metabolites whose levels change significantly during growth. The numbering scheme at the top of each panel does not have any practical significance and simply serves to identify each MS peak in the experiment. Histogram heights correspond to average counts for each metabolite in the triplicates. Measured levels are not indicative of absolute cellular concentrations. However, relative changes in concentrations between samples derived from different growth states may be inferred the histograms. Click here for file

Additional file 8 **Additional figure S4. Spectra of citrulline, phenylalanine, riboflavin and 5-deoxyadenosine**. Paired presentation of ion spectra measured from both *H. salinarum NRC-1 *and from purified metabolite standard for citrulline, phenylalanine, riboflavin and 5-deoxyadenosine. Relative abundance levels are also shown as a histogram. Click here for file
