# Supplementary figures and images for: Large scale physiological readjustment during growth enables rapid, comprehensive and inexpensive systems analysis
Source: BMC Syst Biol. 2010 May 14;4:64. doi: 10.1186/1752-0509-4-64 (PMC2880973; doi:10.1186/1752-0509-4-64)

A

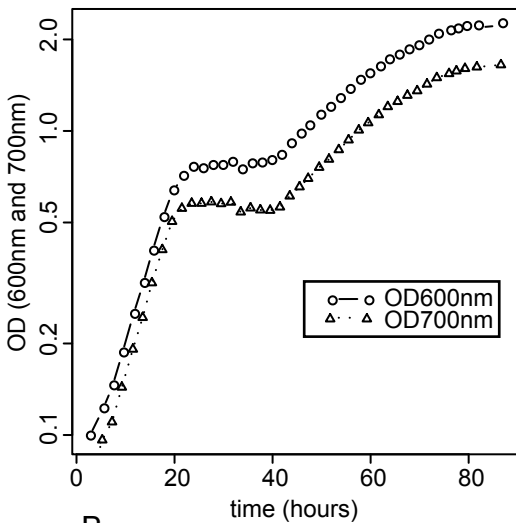

B

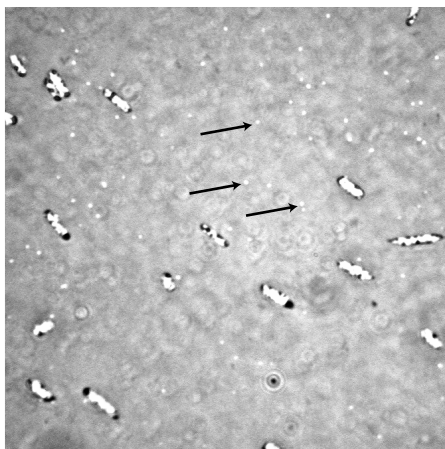

Supplement: Additional file 1 — Additional figure S1. Interrogation of the apparent second "growth" phase of H. salinarum NRC-1 in rich media. (A) Growth of H. salinarum NRC-1 was tracked by optical density measurements at both 600 nm and 700 nm to determine the contribution of bacteriorhodopsin (a protein whose broad absorption peak centered at 568 nm can contribute to absorption at 600 nm but not at 700 nm) accumulation. The growth curve generated at 700 nm displays similar behavior to the curve generated from measurements taken at 600 nm. In particular, the apparent doubling that occurs during what appears to be stationary phase is apparent in both curves suggesting that this is not due to bacteriorhodopsin accumulation. This increase in optical density is most likely due to an increase in light scattering gas vesicles that are visibly released during this late phase of the growth experiment. (B) Phase-contrast visible light microscopy image of H. salinarum NRC-1 near the end of data collection for the data presented in Figure 1 main text. Three arrows point to examples of small bright bodies (presumably gas vesicles) that populate the field of view. This abundance of gas vesicles only becomes apparent after a significant decrease in CFU following stationary phase. [file 1752-0509-4-64-S1.PDF]

A

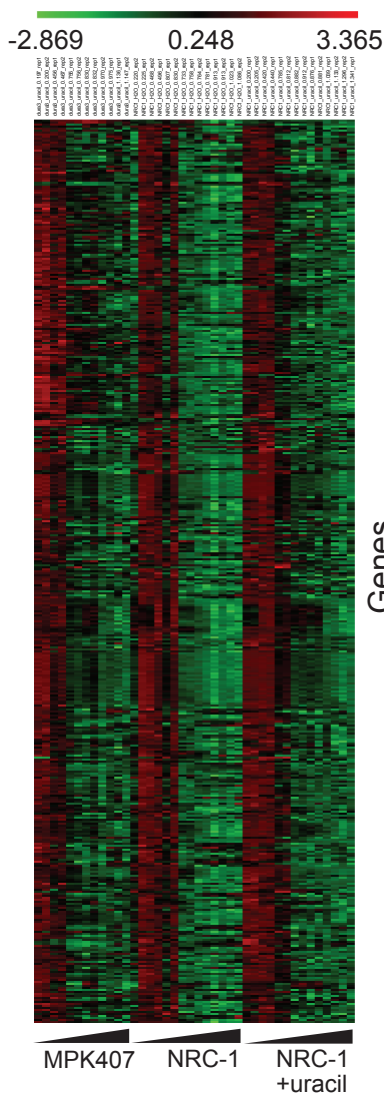

B

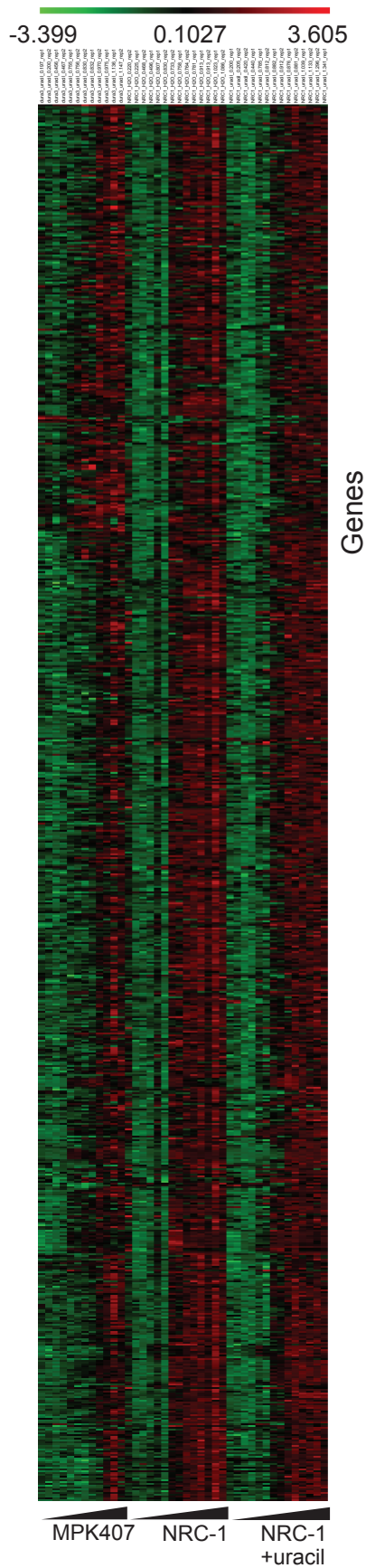

Supplement: Additional file 3 — Additional figure S2. Heat maps of genes with significant up or down regulation during growth. Samples from each individual experiments (e.g. MPK407, H. salinarum NRC-1 and H. salinarum NRC-1 + uracil) are organized by increasing optical density moving from left to right. The black volume bar indicates increasing optical density. Genes have been hierarchically clustered to show potential subpatterns of expression. (A) A heatmap showing the changes in expression of 451 genes whose transcript abundance is decreased upon entry into stationary phase. (B) A heatmap showing the changes in expression of 772 genes whose transcript abundance is increased upon entry into stationary phase. [file 1752-0509-4-64-S3.PDF]
