# Supplementary material for: Large scale physiological readjustment during growth enables rapid, comprehensive and inexpensive systems analysis
Source: BMC Syst Biol. 2010 May 14;4:64. doi: 10.1186/1752-0509-4-64 (PMC2880973; doi:10.1186/1752-0509-4-64)
Supplement: Additional file 2 — Potential causes for the apparent second growth phase in H. salinarum NRC-1. [file 1752-0509-4-64-S2.PDF]

Supplementary Text:

Analysis of Biphasic Growth Curve

The observation of a biphasic growth curve suggests that a second growth phase, amounting to one extra division, might occur in the batch growth of *H. salinarum* NRC-1. However, this interpretation is disputed by the observation of a sharp decrease in colony forming units (CFU) in the same samples (Figure 1 - main text). Several possible explanations can account for an increase in OD_{600} without increase in viable count. The first, is the late-exponential to stationary phase accumulation of the photo-driven ion pump bacteriorhodopsin (bR) [28]. This protein has a broad absorption peak centered at 568nm that could potentially contribute a modest increase in OD_{600}. We eliminated this possibility upon observing increase in optical density at 700nm– significantly outside the maximal absorption range of bR (Supplementary Figure 1A). We therefore suspect that the second possible explanation, increased light scattering by gas vesicles released from lysed cells, likely accounts for the increase in OD_{600} after the cells have entered stationary phase. The noted concomitant decrease in CFUs and extensive gas vesicles release in cultures supports the interpretation of a link between cell death and an increase in OD_{600} (Supplementary Figure 1B). A third explanation could be that a large fraction of the stationary phase cells might adopt a quiescent physiological state that is not easily revived on CM agar plates. Given the large numbers of gas vesicles released during stationary phase, however, this seems like the least plausible explanation to account for the observed concomitant increase in OD_{600} and decrease in viable count. Nevertheless, we cannot discount this possibility. Finally, it is possible that clumping of cells may be responsible. This may be partially responsible for the

dip seen before the second increase in OD_{600} but does not likely account for the following increase in scattering.
