# Supplementary material for: Large scale physiological readjustment during growth enables rapid, comprehensive and inexpensive systems analysis
Source: BMC Syst Biol. 2010 May 14;4:64. doi: 10.1186/1752-0509-4-64 (PMC2880973; doi:10.1186/1752-0509-4-64)
Supplement: Additional file 4 — Additional table S1 - Genes whose transcript abundance is decreased during the transition to stationary phase. This table lists ORF name, gene symbol, an estimate fold change in expression between pre-stationary and stationary phase expression, an indicator of significance of change between pre-stationary and stationary phase expression values and the putative gene function (if known). Fold change was calculated by taking the ratio between the average non-logged ratio for the last four samples (replicates included) taken in the growth curve to the average of the first four samples taken in the growth curve. A t-test, computed on logged data, was also used on the same selected sets to (first and last four data points for each strain) to ask whether the changes in expression were statistically significant given an overall p-value threshold = 0.05 and enforcing a false discovery rate of 0.05 or less. 418 genes of the 451 in this clustering derived set were deemed to have significantly different expression levels using this criteria while the remaining 33 genes did not. Genes meeting this criteria are marked with a number one while those not meeting the criteria are marked with a zero. Manual inspection of expression profiles of genes not meeting the above criteria suggest that the t-test in this instance may be too conservative as many gene expression profiles deemed not significant show what seems to be clear decrease in signal between pre-stationary and stationary phases. [file 1752-0509-4-64-S4.PDF]

Additional table S1

ORF Name	Gene Symbol	Estimate Fold Change	Significance by T-Test	Function
VNG0001H	VNG0001H	0.70	0	
VNG0002G	yvrO	0.48	0	ABC transporter, ATP-binding protein
VNG0005H	VNG0005H	0.23	0	
VNG0013C	VNG0013C	0.31	1	
VNG0042G	ntp	0.01	1	putative transposase
VNG0043H	VNG0043H	0.04	1	
VNG0047G	graD6	0.05	1	Glucose-1-phosphate thymidylyltransferase
VNG0049H	VNG0049H	0.08	1	Protein-L-Isoaspartate O-Methyltransferase (catalyzes protein repair)
VNG0051G	rfbU1	0.14	1	LPS biosynthesis
VNG0060G	lpg	0.22	0	LPS glycosyltransferase
VNG0063G	galE2	0.06	1	UDP-glucose 4-epimerase
VNG0077H	VNG0077H	0.06	1	
VNG0099G	rpl10e	0.02	1	50S ribosomal protein L10e
VNG0101G	cspD1	0.01	1	Cold shock protein (putative regulator)
VNG0106G	rmeM	0.15	0	Type I restriction modification enzyme, M subunit
VNG0127C	VNG0127C	0.42	0	
VNG0133G	rpa	0.03	1	Replication A related protein
VNG0134G	hpyA	0.05	1	Archaeal histone A1
VNG0150H	VNG0150H	0.19	1	cytochrome C biogenesis protein
VNG0152G	prrC	0.03	1	Regulatory protein
VNG0163G	mutS1	0.05	1	Mismatch repair protein
VNG0166G	psmA2	0.04	1	Proteasome alpha subunit
VNG0177G	rpl15e	0.01	1	50S ribosomal protein L15e
VNG0179C	VNG0179C	0.58	0	RIO1 family eukaryotic protein kinase
VNG0183G	xthA	0.06	1	Endonuclease IV
VNG0184H	VNG0184H	0.94	0	
VNG0197C	VNG0197C	0.07	1	
VNG0233H	VNG0233H	0.01	1	
VNG0234C	VNG0234C	0.02	1	Prefoldin beta subunit (GimC beta subunit)
VNG0237H	rpc10	0.03	1	DNA-directed RNA polymerase, 7 kDa subunit
VNG0239C	VNG0239C	0.03	1	50S ribosomal protein L37Ae
VNG0248C	VNG0248C	0.36	0	
VNG0249G	fbr	0.06	1	Copper binding proteins/plastocyanin/azurin
VNG0259G	ipp	0.04	1	Inorganic pyrophosphatase
VNG0285C	VNG0285C	0.01	1	transposase

ORF Name	Gene Symbol	Estimate Fold Change	Significance by T-Test	Function
VNG0286C	VNG0286C	0.01	1	probable transposase
VNG0287H	VNG0287H	0.01	1	
VNG0294G	pnm	0.01	1	N-methyltransferase homolog
VNG0311H	VNG0311H	0.05	1	
VNG0315G	tfbF	0.07	1	Transcription initiation factor IIB 6 (TFIIB 6)
VNG0330G	ppsA	0.01	1	Phosphoenolpyruvate synthase
VNG0373H	VNG0373H	0.05	1	
VNG0374G	nusG	0.03	1	Transcription termination-antitermination factor
VNG0375G	secE	0.02	1	Preprotein translocase secE subunit
VNG0380G	caa	0.06	1	Cation antiporter
VNG0390G	graD1	0.02	1	Glucose-1-phosphate thymidylyltransferase
VNG0399H	VNG0399H	0.20	1	
VNG0403G	proS	0.03	1	Proline-tRNA synthetase
VNG0406C	VNG0406C	0.10	1	Glycerol dehdrogenase
VNG0410G	rfbU2	0.01	1	LPS biosynthesis
VNG0412G	folP	0.17	1	Dihydropteroate synthase
VNG0421C	VNG0421C	0.05	1	
VNG0424C	VNG0424C	0.05	1	NAC-related regulator
VNG0426G	rpoM	0.02	1	DNA-directed RNA-polymerase subunit M
VNG0431G	apa	0.03	1	Diadenosine tetraphosphate pyrophosphohydrolase
VNG0433C	VNG0433C	0.29	0	
VNG0439C	VNG0439C	0.01	1	putative flavoprotein
VNG0468C	VNG0468C	0.09	1	putative flavoprotein
VNG0473G	porB	0.04	1	pyruvate ferredoxin oxidoreductase, subunit beta
VNG0474G	porA	0.04	1	pyruvate ferredoxin oxidoreductase, subunit alpha
VNG0485H	VNG0485H	0.06	1	
VNG0487H	VNG0487H	0.01	1	
VNG0489G	dnaJ	0.08	1	Chaperone protein dnaJ
VNG0491G	dnaK	0.01	1	Chaperone protein dnaK
VNG0494G	grpE	0.02	1	GrpE protein (HSP-70 cofactor)
VNG0496H	VNG0496H	0.04	1	
VNG0509H	VNG0509H	0.03	1	
VNG0537C	VNG0537C	0.03	1	
VNG0540G	imp	0.02	1	Immunogenic protein
VNG0546C	VNG0546C	0.02	1	archaeal proteins of unknown function, PF01908

ORF Name	Gene Symbol	Estimate Fold Change	Significance by T-Test	Function
VNG0548C	VNG0548C	0.01	1	Nucleolar RNA-binding protein
VNG0549G	eif2a	0.01	1	Probable translation initiation factor 2 alpha subunit (eIF-2-alpha)
VNG0550G	rps27e	0.01	1	30S ribosomal protein S27e
VNG0551G	rpl44e	0.01	1	50S ribosomal protein L44E
VNG0559G	apt	0.02	1	Adenine phosphoribosyltransferase
VNG0562C	VNG0562C	0.05	1	
VNG0568C	VNG0568C	0.02	1	putative subunit of a Na+/H+ antiporter complex
VNG0570H	VNG0570Hm	0.02	1	Multisubunit Na+/H+ antiporter, MnhG subunit
VNG0586C	VNG0586C	0.04	1	PetE plastocyanin.
VNG0597H	VNG0597H	0.04	1	
VNG0598C	VNG0598C	0.09	1	predicted glycosyltransferase. Possibly involved in cell wall biosynthesis
VNG0600C	VNG0600C	0.06	1	
VNG0601H	VNG0601H	0.02	1	Predicted RNA-binding protein containing KH domain
VNG0617H	VNG0617H	0.40	0	
VNG0620G	edp	0.55	0	Proteinase IV homolog
VNG0628G	gdhA1	0.01	1	Glutamate dehydrogenase
VNG0635G	nolB	0.01	1	NADH dehydrogenase/oxidoreductase-like protein
VNG0636G	ndhG1	0.01	1	NADH dehydrogenase/oxidoreductase
VNG0637G	ndhG5	0.01	1	NADH dehydrogenase/oxidoreductase
VNG0640G	nolD	0.01	1	NADH dehydrogenase/oxidoreductase-like protein
VNG0641C	VNG0641C	0.01	1	NADH-ubiquinone/plastoquinone oxidoreductase chain 6
VNG0642C	VNG0642C	0.01	1	
VNG0643G	nolC	0.01	1	NADH dehydrogenase/oxidoreductase-like protein
VNG0646G	nuoL	0.01	1	F420H2:quinone oxidoreductase chain L
VNG0647G	nuoM	0.01	1	F420H2:quinone oxidoreductase chain M
VNG0648G	ndhG3	0.01	1	NADH dehydrogenase/oxidoreductase
VNG0650C	VNG0650C	0.75	0	
VNG0659H	VNG0659H	0.01	1	
VNG0662G	coxC	0.01	1	Cytochrome c oxidase subunit III
VNG0665G	coxB1	0.01	1	Cytochrome c oxidase subunit II
VNG0666G	ctaB	0.02	1	Heme synthase
VNG0667G	trp4	0.02	1	ABC transporter, ATP-binding protein homolog
VNG0669H	VNG0669H	0.30	1	
VNG0678G	acaB1	0.02	1	3-ketoacyl-CoA thiolase
VNG0679G	acd4	0.18	1	Acyl-CoA dehydrogenase

ORF Name	Gene Symbol	Estimate Fold Change	Significance by T-Test	Function
VNG0680G	fdfT	0.02	1	Farnesyl-diphosphate farnesyltransferase
VNG0681G	hbd1	0.02	1	3-hydroxyacyl-CoA dehydrogenase
VNG0683C	VNG0683C	0.01	1	Fructose-bisphosphate aldolase
VNG0684G	fbp	0.02	1	Fructose-bisphosphatase
VNG0702H	VNG0702H	0.01	1	putative heavy metal transport protein
VNG0703H	VNG0703H	0.13	0	putative transcription regulator
VNG0724H	VNG0724H	0.12	0	
VNG0741H	VNG0741H	0.04	1	
VNG0754C	VNG0754C	0.03	1	
VNG0762H	VNG0762H	0.05	1	
VNG0767H	VNG0767H	0.02	1	
VNG0769H	VNG0769H	0.14	0	
VNG0771G	aldY2	0.05	1	Aldehyde dehydrogenase (Retinol)
VNG0777G	taqD	0.40	1	Glycerol-3-phosphate cytidyltransferase
VNG0778C	VNG0778C	0.03	1	
VNG0782H	VNG0782H	0.01	1	HEAT repeat-containing protein.
VNG0784G	pssA	0.02	1	CDP-diacylglycerol-serine O-phosphatidyltransferase
VNG0787G	rps3e	0.01	1	30S ribosomal protein S3Ae
VNG0788H	VNG0788H	0.02	1	
VNG0789C	VNG0789C	0.01	1	
VNG0790G	rps15p	0.01	1	30S ribosomal protein S15P
VNG0795G	hcpC	0.03	1	Halocyanin precursor-like
VNG0815G	yfmJ	0.95	0	Quinone oxidoreductase
VNG0857C	VNG0857Cm	0.17	0	Predicted RNA methylase
VNG0860G	rpoL	0.02	1	DNA-directed RNA polymerase subunit L
VNG0862G	hisF	0.03	1	Imidazole glycerol phosphate synthase subunit hisF
VNG0863H	VNG0863H	0.01	1	
VNG0865C	VNG0865C	0.05	1	
VNG0867G	asnA	0.02	1	Asparagine synthetase
VNG0872G	gatA	0.01	1	Glu-tRNA amidotransferase
VNG0874G	traB	0.17	1	Possible signaling protein/possible conjugation(mating) protein
VNG0889G	gyrA	0.06	1	DNA gyrase subunit A
VNG0891G	yjlD	0.02	1	NADH dehydrogenase
VNG0892H	VNG0892H	0.03	1	
VNG0893G	udp2	0.03	1	Uridine phosphorylase

ORF Name	Gene Symbol	Estimate Fold Change	Significance by T-Test	Function
VNG0896G	cda	0.04	1	Cytidine aminohydrolase
VNG0897G	rbsC1	0.02	1	ABC transporter permease
VNG0898G	rbsC2	0.06	1	ABC transporter permease
VNG0901G	rbsA	0.01	1	ABC transporter ATP-binding
VNG0903C	VNG0903C	0.02	1	
VNG0905G	pmu2	0.02	1	Phosphomannomutase
VNG0923G	sfuB	0.02	1	Putative Iron transporter-like protein
VNG0924G	ibp	0.05	1	ABC transporter periplasmic substrate binding component
VNG0925C	VNG0925C	0.08	1	
VNG0940G	acs3	0.19	0	Acetyl-CoA synthetase (ADP forming)
VNG0943C	VNG0943C	0.28	0	
VNG0982C	VNG0982C	0.03	1	
VNG0983C	VNG0983C	0.04	1	
VNG0987H	VNG0987H	0.07	1	
VNG1001G	guaB	0.08	1	Inosine monophosphate dehydrogenase
VNG1027G	tpiA	0.10	1	Triosephosphate isomerase
VNG1034H	VNG1034H	0.02	1	
VNG1053G	gtl	0.08	1	Glycosyl transferase-like
VNG1056C	VNG1056C	0.22	0	
VNG1058H	VNG1058H	0.01	1	
VNG1062G	rfbQ	0.25	0	Rhamnosyl transferase
VNG1067G	exoM	0.02	1	Succinoglycan biosynthesis protein
VNG1068G	tot	0.01	1	Transmembrane oligosaccharyl transferase
VNG1069C	VNG1069C	0.83	0	
VNG1089G	purA	0.06	1	Adenylosuccinate synthetase
VNG1090H	VNG1090H	0.05	1	
VNG1097G	cysS	0.03	1	Cysteinyl-tRNA synthetase
VNG1103G	rpl12p	0.01	1	50S ribosomal protein L12P ('A' type) (HL20)
VNG1104G	rpl10p	0.01	1	Acidic ribosomal protein P0 homolog (L10E)
VNG1105G	rpl1p	0.01	1	50S ribosomal protein L1P (HL8)
VNG1108G	rpl11p	0.01	1	50S ribosomal protein L11P
VNG1110C	VNG1110C	0.01	1	
VNG1111G	drg	0.01	1	Hypothetical protein Vng1111g
VNG1114G	glo1	0.02	1	Glyoxalase
VNG1117C	VNG1117C	0.09	1	

ORF Name	Gene Symbol	Estimate Fold Change	Significance by T-Test	Function
VNG1125G	korB	0.03	1	Putative 2-ketoglutarate ferredoxin oxidoreductase (Beta)
VNG1128G	korA	0.02	1	Putative 2-ketoglutarate ferredoxin oxidoreductase (Alpha)
VNG1132G	rps13p	0.01	1	30S ribosomal protein S13P/S18E (HS13)
VNG1133G	rps4p	0.01	1	30S ribosomal protein S4P
VNG1134G	rps11p	0.01	1	30S ribosomal protein S11P
VNG1136G	rpb3	0.01	1	DNA-directed RNA polymerase subunit D
VNG1137G	rpl18e	0.01	1	50S ribosomal protein L18e (HeL18)
VNG1138G	rpl13p	0.01	1	50S ribosomal protein L13P
VNG1139G	rps9p	0.01	1	30S ribosomal protein S9P
VNG1140G	rpoN	0.01	1	DNA-directed RNA polymerase subunit N
VNG1141G	rpoK	0.01	1	DNA-directed RNA polymerase subunit K
VNG1142G	eno	0.01	1	Enolase
VNG1143G	rps2p	0.01	1	30S ribosomal protein S2P
VNG1145G	mvk	0.08	1	Mevalonate kinase
VNG1149C	VNG1149Cm	0.01	1	Predicted hydrolase (metallo-beta-lactamase superfamily)
VNG1150G	idsA	0.01	1	Geranylgeranyl diphosphate synthase
VNG1157G	rphs6	0.01	1	50S ribosomal protein L7Ae
VNG1158G	rps28e	0.01	1	30S ribosomal protein S28E
VNG1159G	rpl24e	0.02	1	50S ribosomal protein L24E (LSU ribosomal protein L24E)
VNG1160G	ndk	0.02	1	Nucleoside diphosphate kinase
VNG1169C	VNG1169C	0.01	1	RNA polymerase Rpb4
VNG1170G	rpl21e	0.01	1	50S ribosomal protein L21e
VNG1173G	eef1b	0.01	1	Elongation factor 1-beta (EF-1-beta) (aEF-1beta)
VNG1190G	sod1	0.02	1	Superoxide dismutase [Mn] 1
VNG1196H	VNG1196H	0.08	1	
VNG1204G	gdhA2	0.03	1	Glutamate dehydrogenase
VNG1215G	pai1	0.04	1	acetyl transferase (histone)
VNG1219G	urk	0.75	0	Uridine kinase
VNG1220H	VNG1220H	0.01	1	
VNG1227H	VNG1227H	0.02	1	
VNG1241G	surE	0.03	1	Acid phosphatase surE
VNG1246H	VNG1246H	0.28	0	
VNG1257H	VNG1257H	0.02	1	putative cytochrome oxidase
VNG1263C	VNG1263C	0.07	1	Phosphotransferase system IIC components
VNG1289H	VNG1289H	0.04	1	

ORF Name	Gene Symbol	Estimate Fold Change	Significance by T-Test	Function
VNG1294G	slyD	0.07	1	FK506 binding protein (Peptidyl-prolyl cis-trans isomerase
VNG1297C	VNG1297C	0.12	1	S-adenosylmethionine synthetase
VNG1308G	sdhB	0.01	1	Succinate dehydrogenase subunit B
VNG1309G	sdhD	0.01	1	Membrane anchor
VNG1310G	sdhC	0.01	1	Succinate dehydrogenase hydrophobic membrane anchor protein
VNG1325C	VNG1325C	0.02	1	Thymidylate synthase complementing protein
VNG1329H	VNG1329H	0.09	1	
VNG1344G	dchpS	0.10	1	Dihydropteroate synthase
VNG1356G	fumC	0.02	1	Fumarate hydratase
VNG1357C	VNG1357C	0.04	1	
VNG1376H	VNG1376H	0.07	1	
VNG1408G	ush	0.04	1	5'-nucleotidase/2',3'-cyclic phosphodiesterase and related esterases;
VNG1416G	folD	0.09	1	Methylenetetrahydrofolate dehydrogenase
VNG1422H	VNG1422H	0.41	1	
VNG1432G	dhs	0.02	1	Probable deoxyhypusine synthase
VNG1433G	rps17e	0.02	1	30S ribosomal protein S17e
VNG1438H	VNG1438H	0.11	1	
VNG1452G	eif2bd	0.10	1	Translation initiation factor eIF-2B subunit delta
VNG1466H	VNG1466H	0.03	1	
VNG1473H	VNG1473H	0.01	1	
VNG1483C	VNG1483C	0.04	1	putative transcription regulator
VNG1487H	VNG1487H	0.02	1	
VNG1488G	boa2	0.02	1	Bacterio-opsin activator-like protein
VNG1494G	rpl37e	0.03	1	50S ribosomal protein L37e
VNG1496G	snp	0.04	1	snRNP homolog
VNG1506G	pelA	0.15	1	Putative peptide chain release factor pelota
VNG1511C	VNG1511C	0.03	1	putative recJ-like exonuclease
VNG1530H	VNG1530H	0.11	1	
VNG1538H	VNG1538H	0.05	1	
VNG1541G	sucC	0.01	1	Succinyl-CoA synthetase beta chain
VNG1542G	sucD	0.01	1	Succinyl-CoA synthetase alpha chain
VNG1543G	zim	0.01	1	CTAG modification methylase
VNG1550G	cbiT	0.01	1	Putative precorrin 8-w decarboxylase (AdoMet methyltransferase).
VNG1551G	cbiL	0.01	1	Precorrin-2 C20 methyltransferase
VNG1553G	cbiF	0.02	1	Precorrin 4-methyltransferase

ORF Name	Gene Symbol	Estimate Fold Change	Significance by T-Test	Function
VNG1554G	cbiG	0.02	1	Cobalamin biosynthesis
VNG1555G	cbiH1	0.01	1	precorrin-3B C17-methyltransferase
VNG1557G	cbiH2	0.01	1	precorrin-3 C-17 methyltransferase
VNG1558H	VNG1558H	0.01	1	putative ferredoxin
VNG1559H	VNG1559H	0.01	1	
VNG1561C	CbiX, putative	0.01	1	Putative cobaltochelatase with Ferredoxin domain
VNG1562H	VNG1562H	0.01	1	
VNG1564H	VNG1564H	0.01	1	
VNG1566G	cobN	0.01	1	Cobalamin biosynthesis protein
VNG1567G	cbiC	0.01	1	Precorrin isomerase
VNG1568G	cbiJ	0.01	1	Precorrin-3 methylase
VNG1585C	VNG1585Cm	0.01	1	Uncharacterized conserved protein
VNG1591H	VNG1591H	0.02	1	
VNG1606G	gcvT1	0.01	1	Aminomethyltransferase
VNG1608C	VNG1608C	0.02	1	
VNG1611C	VNG1611C	0.01	1	
VNG1616C	VNG1616C	0.04	1	putative transcriptional regulator
VNG1624G	mdh	0.01	1	malic enzyme (type III)
VNG1632G	cbiQ	0.02	1	Cobalt transport protein
VNG1653H	VNG1653H	0.01	1	transposase
VNG1668G	rps8e	0.02	1	30S ribosomal protein S8e
VNG1688C	VNG1688C	0.01	1	
VNG1689G	rpl3p	0.01	1	50S ribosomal protein L13P
VNG1690G	rpl4e	0.01	1	50S ribosomal protein L4E
VNG1691G	rpl23p	0.01	1	50S ribosomal protein L23P
VNG1692G	rpl2p	0.01	1	50S ribosomal protein L2P
VNG1693G	rps19p	0.01	1	30S ribosomal protein S19P (HHAS19)
VNG1695G	rpl22p	0.01	1	50S ribosomal protein L22P (HHAL22)
VNG1697G	rps3p	0.01	1	30S ribosomal protein S3P (HS4) (HHAS3)
VNG1698G	rpl29p	0.01	1	50S ribosomal protein L29P (HHAL29)
VNG1699C	VNG1699C	0.01	1	putative RNAse P residing within ribosomal operon
VNG1700G	rps17p	0.01	1	30S ribosomal protein S17 (HHAS17)
VNG1701G	rpl14p	0.01	1	50S ribosomal protein L14P (HHAL14)
VNG1702G	rpl24p	0.01	1	50S ribosomal protein L24P
VNG1703G	rps4e	0.01	1	30S ribosomal protein S4e

ORF Name	Gene Symbol	Estimate Fold Change	Significance by T-Test	Function
VNG1705G	rpl5p	0.01	1	50S ribosomal protein L5P (HSal5)
VNG1706G	rps14p	0.01	1	30S ribosomal protein S14P
VNG1707G	rps8p	0.01	1	30S ribosomal protein S8P
VNG1709G	rpl6p	0.01	1	50S ribosomal protein L6P
VNG1711G	rpl32e	0.01	1	50S ribosomal protein L32E
VNG1713G	rpl19e	0.01	1	Ribosomal protein L19
VNG1714G	rpl18p	0.01	1	50S ribosomal protein L18P (HSal18)
VNG1715G	rps5p	0.01	1	30S ribosomal protein S5P
VNG1716G	rpl30p	0.01	1	50S ribosomal protein L30P
VNG1718G	rpl15p	0.01	1	50S ribosomal protein L15P
VNG1719G	secY	0.01	1	Protein translocase
VNG1724G	adk	0.01	1	Adenylate kinase
VNG1726G	htlB	0.02	1	Htr-like protein
VNG1727G	cmk	0.02	1	Cytidylate kinase
VNG1729G	sus	0.12	1	Probable tRNA pseudouridine synthase B
VNG1749G	gbp1	0.48	0	GTP-binding protein homolog
VNG1752C	VNG1752C	0.12	1	
VNG1767G	speB	0.01	1	Agmatinase
VNG1768G	eif5a	0.01	1	Translation initiation factor 5A (eIF-5A) (Hypusine-containing protein)
VNG1774G	hemA	0.01	1	Glutamyl-tRNA reductase
VNG1775C	VNG1775C	0.03	1	Siroheme Biosynthesis Protein
VNG1777H	VNG1777H	0.12	1	
VNG1779C	VNG1779C	0.81	0	
VNG1782C	VNG1782C	0.04	1	
VNG1804G	noxA	0.01	1	NADH oxidase
VNG1830G	pyrG	0.01	1	CTP synthase/GATase (glutamyl amidotransferase)
VNG1836G	cspD2	0.01	1	Cold shock protein
VNG1844G	ansA	0.02	1	L-asparaginase
VNG1850G	udp1	0.01	1	Uridine phosphorylase
VNG1873G	icd	0.02	1	Isocitrate dehydrogenase, NADP
VNG1877C	VNG1877C	0.05	1	
VNG1889H	VNG1889H	0.04	1	
VNG1907H	VNG1907H	0.08	1	
VNG1916H	VNG1916H	0.01	1	
VNG1926G	pdhA1	0.01	1	Pyruvate dehydrogenase alpha subunit

ORF Name	Gene Symbol	Estimate Fold Change	Significance by T-Test	Function
VNG1933G	ftsZ3	0.01	1	Cell division protein
VNG1934H	VNG1934H	0.01	1	
VNG1935C	VNG1935C	0.10	1	
VNG1939G	purC	0.80	0	Putative phosphoribosylaminoimidazole-succinocarboxamide synthase
VNG1948H	VNG1948H	0.03	1	
VNG1995C	VNG1995C	0.02	1	
VNG1997G	infB	0.07	1	Probable translation initiation factor IF-2
VNG2011G	thiL	0.03	1	Thiamine monophosphate kinase
VNG2015C	VNG2015C	0.02	1	Amino acid kinase family protein
VNG2017G	lysS	0.01	1	Lysyl-tRNA synthetase
VNG2020C	VNG2020C	0.01	1	predicted transcriptional regulator (marR/padR family)
VNG2021C	VNG2021C	0.02	1	monooxygenase likely involved in antibiotic synthesis
VNG2039H	VNG2039H	0.03	1	
VNG2043G	ham1	0.01	1	HAM1 protein homolog
VNG2045G	gcp	0.02	1	O-sialoglycoprotein endopeptidase homolog
VNG2047G	rps27ae	0.04	1	30S ribosomal protein S27E
VNG2048G	rps24e	0.01	1	30S ribosomal protein S24e
VNG2049C	VNG2049C	0.13	1	
VNG2051G	rpoE''	0.01	1	DNA-directed RNA polymerase subunit E''
VNG2053G	rpoE'	0.01	1	DNA-directed RNA polymerase subunit E'
VNG2054H	VNG2054H	0.01	1	
VNG2056G	eif2g	0.01	1	Probable translation initiation factor 2 gamma subunit (eIF-2-gamma)
VNG2063G	aca	0.04	1	Probable acetyl-CoA acetyltransferase
VNG2065G	dgs	0.12	1	Dolichol-P-glucose transferase
VNG2072G	serS	0.02	1	Seryl-tRNA synthetase
VNG2076G	rpl40e	0.02	1	50S ribosomal protein L40E
VNG2084G	phnE	0.05	1	Transport protein
VNG2093G	glnA	0.02	1	Glutamine synthetase
VNG2098H	VNG2098H	0.02	1	
VNG2099C	VNG2099C	0.05	1	Endoribonuclease L-PSP
VNG2102G	citZ	0.07	1	Citrate synthase
VNG2106G	sdh	0.05	1	Succinate dehydrogenase subunit
VNG2118G	pyrE2	0.01	1	Orotate phosphoribosyltransferase
VNG2128C	VNG2128C	0.05	1	
VNG2129H	VNG2129H	0.03	1	

ORF Name	Gene Symbol	Estimate Fold Change	Significance by T-Test	Function
VNG2135G	atpD	0.01	1	V-type ATP synthase subunit D
VNG2138G	atpB	0.01	1	V-type ATP synthase beta chain
VNG2139G	atpA	0.01	1	V-type ATP synthase alpha chain
VNG2140G	atpF	0.01	1	V-type ATP synthase subunit F
VNG2141G	atpC	0.01	1	V-type ATP synthase subunit C
VNG2143G	atpK	0.01	1	H+-transporting ATP synthase subunit K
VNG2144G	atpI	0.01	1	V-type ATP synthase subunit I
VNG2146H	atpH	0.01	1	Archaeal/vacuolar-type H+-ATPase subunit H
VNG2149H	VNG2149H	0.15	1	
VNG2150G	etfB	0.01	1	Electron transfer flavoprotein subunit beta
VNG2151G	etfA	0.01	1	Electron transfer flavoprotein subunit alpha
VNG2153G	fps	0.07	1	Putative isopentenyl pyrophosphate isomerase
VNG2173G	rad24a	0.12	1	DNA repair protein
VNG2178H	VNG2178H	0.65	0	
VNG2181G	mcm	0.03	1	MCM / cell division control protein 21
VNG2189H	VNG2189H	0.06	1	
VNG2190G	ileS	0.04	1	Isoleucyl-tRNA synthetase
VNG2205H	VNG2205H	0.02	1	
VNG2206G	pmu1	0.02	1	Phosphomannomutase
VNG2208G	trpS1	0.04	1	Tryptophanyl-tRNA synthetase
VNG2210G	endA	0.44	0	tRNA intron endonuclease
VNG2213G	brr2	0.07	1	Pre-mRNA splicing helicase
VNG2216G	lip	0.11	1	Probable lipoic acid synthetase (Lip-syn) (Lipoate synthase)
VNG2217G	pdhA2	0.01	1	Pyruvate dehydrogenase alpha subunit
VNG2218G	pdhB	0.01	1	Pyruvate dehydrogenase beta subunit
VNG2219G	dsa	0.01	1	Dihydrolipoamide S-acetyltransferase
VNG2220G	lpdA	0.01	1	Dihydrolipoamide dehydrogenase
VNG2224G	ocd1	0.01	1	Ornithine cyclodeaminase
VNG2226G	cctA	0.03	1	Thermosome alpha subunit
VNG2243G	tbpE	0.05	1	TATA-box binding protein E (TATA-box factor E)
VNG2244H	VNG2244H	0.10	1	
VNG2253H	VNG2253H	0.03	1	
VNG2273H	VNG2273H	0.02	1	
VNG2282C	VNG2282C	0.02	1	
VNG2293G	fer2	0.05	1	Ferredoxin

ORF Name	Gene Symbol	Estimate Fold Change	Significance by T-Test	Function
VNG2302G	yuxL	0.26	1	Acylaminoacyl-peptidase
VNG2332G	cysG	0.49	1	Uroporphyrin-III C-methyltransferase
VNG2333C	VNG2333C	0.24	1	recJ-like phosphoesterase (endonuclease)
VNG2341H	VNG2341H	0.01	1	
VNG2342H	VNG2342H	0.03	1	
VNG2343G	ykfD	0.01	1	Oligopeptide ABC transporter ATP-binding
VNG2344G	oppD2	0.02	1	Oligopeptide ABC transporter
VNG2351C	VNG2351C	0.03	1	
VNG2395C	VNG2395C	0.02	1	
VNG2415H	VNG2415H	0.16	1	
VNG2462G	dpa	0.01	1	Signal recognition particle receptor
VNG2465C	VNG2465C	0.02	1	Prefoldin alpha subunit (GimC alpha subunit)
VNG2467G	rpl31e	0.01	1	50S ribosomal protein L31e
VNG2469G	rpl39e	0.01	1	50S ribosomal protein L39e
VNG2473G	radA1	0.02	1	DNA repair and recombination protein radA
VNG2509H	VNG2509H	0.01	1	
VNG2514G	rps6e	0.01	1	30S ribosomal protein S6e
VNG2515H	VNG2515H	0.07	1	
VNG2519H	VNG2519H	0.47	0	
VNG2533G	pyrC	0.02	1	Dihydroorotase
VNG2541C	VNG2541Cm	0.82	1	Nicotinic acid phosphoribosyltransferase
VNG2547G	valS	0.01	1	Valyl-tRNA synthetase
VNG2553G	yqeC	0.08	1	6-phosphogluconate dehydrogenase
VNG2555C	VNG2555C	0.01	1	putative ferredoxin
VNG2556H	VNG2556H	0.15	1	
VNG2558G	fepC	0.17	1	Ferric enterobactin transport protein
VNG2562H	VNG2562H	0.59	0	periplasmic binding protein, probably involved in iron transport
VNG2570G	dcd	0.20	1	Deoxycytidine triphosphate deaminase
VNG2574G	can	0.01	1	Aconitase
VNG2575G	rimI	0.01	1	Pab N-terminal acetyltransferase
VNG2584C	VNG2584C	0.02	1	Translation initiation factor SUI1
VNG2608C	VNG2608C	0.56	0	
VNG2612G	rli	0.01	1	RNase L inhibitor homolog
VNG2613H	VNG2613H	0.02	1	
VNG2614H	VNG2614H	0.06	1	putative transcription regulator

ORF Name	Gene Symbol	Estimate Fold Change	Significance by T-Test	Function
VNG2639G	uae	0.04	1	UDP-N-acetylglucosamine 2-epimerase
VNG2642H	VNG2642H	0.09	1	
VNG2643H	VNG2643H	0.02	1	
VNG2646C	VNG2646C	0.20	1	
VNG2648G	rps10p	0.02	1	30S ribosomal protein S10P
VNG2649G	eef1a	0.02	1	Elongation factor 1-alpha
VNG2652H	VNG2652H	0.02	1	
VNG2654G	eef2	0.01	1	Translation elongation factor eEF-2
VNG2657G	rps7p	0.01	1	30S ribosomal protein S7P
VNG2658G	rps12p	0.01	1	30S ribosomal protein S12P (HmaS12)
VNG2661G	nusA	0.01	1	NusA protein homolog
VNG2664G	rpoA	0.11	1	DNA-directed RNA polymerase subunit A
VNG2666G	rpoB''	0.06	1	DNA-directed RNA polymerase subunit B
VNG2668G	rpoH	0.03	1	DNA-directed RNA polymerase subunit H
VNG2669G	cyo	0.20	1	Cytochrome oxidase subunit I homolog
VNG2679G	csg	0.02	1	Cell surface glycoprotein
VNG5008H	VNG5008H	0.01	1	
VNG5009H	VNG5009H	0.14	1	transcription regulator
VNG5012H	VNG5012H	0.09	1	
VNG5042H	VNG5042H	0.04	1	transposase
VNG5049H	VNG5049H	0.11	1	
VNG5144H	VNG5144H	0.04	1	Transcriptional regulator PadR-like family
VNG5146H	VNG5146H	0.03	1	
VNG5173H	VNG5173H	0.02	1	
VNG6188H	VNG6188H	0.01	1	
VNG6205C	VNG6205C	0.22	1	
VNG6221H	VNG6221H	0.37	1	
VNG6302C	VNG6302C	0.30	1	
VNG6308G	gltP	0.14	1	Proton/sodium-glutamate symport protein
VNG6309G	pyrB	0.03	1	Aspartate carbamoyltransferase
VNG6311G	pyrI	0.01	1	Aspartate carbamoyltransferase regulatory chain
