# Supplementary material for: Large scale physiological readjustment during growth enables rapid, comprehensive and inexpensive systems analysis
Source: BMC Syst Biol. 2010 May 14;4:64. doi: 10.1186/1752-0509-4-64 (PMC2880973; doi:10.1186/1752-0509-4-64)
Supplement: Additional file 5 — Additional table S2 - Genes whose transcript abundance is increased during the transition to stationary phase. This table lists ORF name, gene symbol, an estimate fold change in expression between pre-stationary and stationary phase expression, an indicator of significance of change between pre-stationary and stationary phase expression values and the putative gene function (if known). Fold change was calculated by taking the ratio between the average non-logged ratio for the last four samples (replicates included) taken in the growth curve to the average of the first four samples taken in the growth curve. A t-test, computed on logged data, was also used on the same selected sets to (first and last four data points for each strain) to ask whether the changes in expression were statistically significant given an overall p-value threshold = 0.05 and enforcing a false discovery rate of 0.05 or less. 713 genes of the 772 in this clustering derived set were deemed to have significantly different expression levels using this criteria while the remaining 59 genes did not. Genes meeting this criteria are marked with a number one while those not meeting the criteria are marked with a zero. Manual inspection of expression profiles of genes not meeting the above criteria suggest that the t-test in this instance may be too conservative as many gene expression profiles deemed not significant show what seems to be clear increase in signal between pre-stationary and stationary phases. [file 1752-0509-4-64-S5.PDF]

Additional table S2

ORF Name	Gene Symbol	Estimate Fold Change	Significance by T-Test	Function
VNG0003C	VNG0003C	2.65	0	
VNG0018H	VNG0018H	61.89	1	putative peroxidase
VNG0020H	VNG0020H	46.07	1	
VNG0022H	VNG0022H	49.01	1	
VNG0024H	VNG0024H	42.18	1	
VNG0026C	VNG0026C	14.45	1	
VNG0028C	VNG0028C	101.84	1	Putative transposase
VNG0030H	VNG0030H	55.19	1	
VNG0033H	VNG0033H	72.05	1	
VNG0034H	VNG0034H	58.12	1	
VNG0037H	VNG0037H	17.45	0	
VNG0053H	VNG0053H	49.01	1	
VNG0057H	VNG0057H	51.71	1	
VNG0062G	lpb	34.15	1	LPS biosynthesis protein
VNG0064G	graD3	97.71	1	Glucose-1-phosphate thymidylyltransferase
VNG0066H	VNG0066H	93.49	1	potential transcriptional regulator
VNG0067H	VNG0067H	162.67	1	
VNG0068H	VNG0068H	71.96	1	
VNG0069H	VNG0069H	70.01	1	
VNG0070H	relE	60.85	1	putative cytotoxic translational repressor
VNG0073C	VNG0073C	59.89	1	
VNG0079H	VNG0079H	62.04	1	
VNG0086G	moeA2	124.29	1	Molybdenum cofactor biosynthesis protein
VNG0089G	pimT1	54.24	1	L-isoaspartyl protein carboxyl methyltransferase
VNG0090G	moeA1	125.30	1	Molybdenum cofactor biosynthesis protein
VNG0097G	hsp2	26.81	1	Putative heat shock protein
VNG0104G	serA3	72.82	1	Phosphoglycerate dehydrogenase
VNG0105H	VNG0105H	144.48	1	
VNG0115G	yusZ1	117.99	1	Oxidoreductase
VNG0119H	VNG0119H	32.38	1	
VNG0121H	VNG0121H	108.96	1	
VNG0138H	VNG0138H	98.85	1	
VNG0154G	merA	168.34	1	putative dihydrolipoamide Dehydrogenase
VNG0156C	VNG0156C	160.27	1	putative transcription regulator
VNG0159G	mutL	74.18	1	DNA mismatch repair protein mutL

ORF Name	Gene Symbol	Estimate Fold Change	Significance by T-Test	Function
VNG0161G	gdhB	140.05	1	NADP-specific glutamate dehydrogenase B
VNG0162G	alkK	84.68	1	Medium-chain acyl-CoA ligase
VNG0167H	VNG0167H	89.55	1	
VNG0170C	VNG0170Cm	51.50	1	Predicted dithiol-disulfide isomerase involved in polyketide biosynthesis
VNG0174G	cat1	12.51	1	Cationic amino acid transporter
VNG0178H	VNG0178H	16.81	0	
VNG0180G	hop	92.73	1	Halorhodopsin precursor (HR)
VNG0181G	lpl	99.66	1	Lipoate protein ligase
VNG0189C	VNG0189C	74.58	1	putative HD superfamily phosphohydrolase
VNG0215C	VNG0215C	70.46	1	
VNG0216H	VNG0216H	91.79	1	
VNG0218G	gspE1	107.63	1	Type II secretion system protein
VNG0223G	moxR	81.37	1	Methanol dehydrogenase regulatory protein
VNG0226G	htrA	108.35	1	Serine proteinase
VNG0230C	VNG0230C	38.11	1	
VNG0231C	VNG0231C	109.56	1	
VNG0243C	VNG0243Cm	96.21	1	Putative tRNA pseudouridine synthase D (TruD)
VNG0244H	VNG0244H	130.85	1	
VNG0254G	tfbG	32.65	1	Transcription initiation factor IIB 7 (TFIIB 7)
VNG0261H	VNG0261H	135.78	1	
VNG0262C	VNG0262C	157.80	1	
VNG0270C	VNG0270C	28.85	0	
VNG0271C	VNG0271C	31.74	1	
VNG0274C	VNG0274C	177.65	1	
VNG0277G	crtI3	154.29	1	oxidoreductase, previously annotated as
VNG0282H	VNG0282H	40.20	1	
VNG0289H	VNG0289H	143.68	1	
VNG0291H	VNG0291H	131.51	1	
VNG0293H	VNG0293H	108.26	1	putative transcription regulator
VNG0297H	VNG0297H	134.43	1	putative metal-dependent hydrolase
VNG0305G	trpC	51.90	1	Indole-3-glycerol phosphate synthase
VNG0309C	VNG0309C	29.93	1	2-amino-3,7-dideoxy-D-threo-hept-6-ulosonate synthase
VNG0319H	VNG0319H	80.02	1	
VNG0321G	ids	11.84	0	putative membrane-associated protease with homology to HflC
VNG0322H	VNG0322H	45.76	1	

ORF Name	Gene Symbol	Estimate Fold Change	Significance by T-Test	Function
VNG0329G	caaX	22.69	0	Zinc metalloproteinase homolog
VNG0331H	VNG0331H	79.22	1	
VNG0339H	VNG0339H	47.85	1	
VNG0340C	VNG0340C	7.40	0	
VNG0342G	smc1	24.71	1	Chromosome segregation
VNG0346H	VNG0346H	88.77	1	
VNG0347H	VNG0347H	34.10	1	
VNG0349G	topA	34.76	1	DNA topoisomerase I
VNG0352H	VNG0352H	16.97	0	
VNG0354C	VNG0354C	111.23	1	putative metal-binding membrane protease
VNG0355G	htr14	40.09	1	Htr14 transducer
VNG0357H	VNG0357H	60.78	1	
VNG0359C	VNG0359C	25.31	1	
VNG0360C	VNG0360C	29.36	1	
VNG0363G	nfi	50.12	1	Endonuclease V
VNG0365G	arsA1	120.71	1	Arsenical pump-driving ATPase
VNG0367H	VNG0367H	136.20	1	
VNG0368C	VNG0368C	115.17	1	
VNG0382G	aroE	101.45	1	Shikimate 5-dehydrogenase
VNG0384G	trpE2	73.58	1	Anthranilate synthase alpha chain
VNG0387G	ilvE1	99.52	1	Branched-chain amino acid aminotransferase
VNG0388C	VNG0388C	47.13	1	
VNG0389C	VNG0389C	121.63	1	putative transcription regulator
VNG0402H	VNG0402H	43.15	1	
VNG0407H	VNG0407H	59.82	1	
VNG0419C	VNG0419C	47.80	1	
VNG0422G	cyc	110.04	1	Putative cytochrome P450 174A1
VNG0427G	yfmO2	24.75	1	transporter of unknown specificity
VNG0437C	VNG0437C	124.96	1	
VNG0440C	VNG0440C	71.68	1	
VNG0442G	rspA	4.84	0	Starvation sensing protein of the muconate lactonizing enzyme family
VNG0444G	dapA	13.21	0	Dihydrodipicolinate synthase
VNG0446G	gcd	73.83	1	Glucose dehydrogenase
VNG0447H	VNG0447H	56.60	1	
VNG0448G	pyrE1	26.21	1	PyrE-like protein

ORF Name	Gene Symbol	Estimate Fold Change	Significance by T-Test	Function
VNG0458G	prp1	62.94	1	Phosphate regulatory protein homolog
VNG0462C	VNG0462C	50.47	1	putative transcription regulator (ArsR family)
VNG0467G	yafB	194.08	1	Aldehyde reductase
VNG0469H	VNG0469H	68.81	1	
VNG0470G	trp3	51.96	1	Daunorubicin resistance ABC transporter ATP-binding protein
VNG0482H	VNG0482H	127.17	1	
VNG0484G	acs1	76.11	1	Acetyl-CoA synthetase
VNG0498C	VNG0498C	28.54	1	
VNG0499G	cna	91.31	1	putative nucleotide methyltransferase
VNG0500G	ppd	50.47	1	3-isopropylmalate dehydratase
VNG0502G	aspB1	61.22	1	Aspartate aminotransferase
VNG0503C	VNG0503C	80.45	1	putative methyltransferase.
VNG0512G	ppe	64.72	1	DNA double-strand break repair exonuclease Mre11.
VNG0516H	VNG0516H	115.76	1	
VNG0518H	VNG0518H	95.03	1	
VNG0520H	VNG0520H	104.25	1	
VNG0523G	inb	124.67	1	Oxidoreductase homolog
VNG0535C	VNG0535C	62.31	1	
VNG0536G	sirR	44.77	1	Transcription repressor
VNG0539C	VNG0539C	2.98	0	
VNG0542C	VNG0542C	128.65	1	putative oxidoreductase
VNG0553C	VNG0553C	116.89	1	
VNG0557H	VNG0557H	71.91	1	putative intracellular cysteine protease homolog of Pyrococcus PfpI
VNG0573C	VNG0573C	105.10	1	
VNG0574C	VNG0574C	25.56	0	
VNG0591C	VNG0591C	41.69	1	putative transcription regulator
VNG0594H	VNG0594H	137.33	1	
VNG0595H	VNG0595H	108.69	1	
VNG0596H	VNG0596H	80.59	1	
VNG0604H	VNG0604H	130.71	1	
VNG0612H	VNG0612H	55.29	1	
VNG0614G	htr16	167.51	1	Htr16 transducer
VNG0622H	VNG0622H	126.85	1	putative NAD/FAD-containing oxidoreductase
VNG0632G	purK	80.11	1	Phosphoribosylaminoimidazole carboxylase ATP binding subunit
VNG0633G	purE	35.53	1	Phosphoribosylaminoimidazole carboxylase catalytic subunit

ORF Name	Gene Symbol	Estimate Fold Change	Significance by T-Test	Function
VNG0651G	imd1	231.93	1	Hypothetical protein VNG0651G
VNG0652H	VNG0652H	91.07	1	
VNG0653G	mcmA1_2	82.14	1	Methylmalonyl-CoA mutase, subunit alpha
VNG0654C	VNG0654C	158.60	1	putative histone acetyl transferase
VNG0673G	mcmA2	35.58	1	Methylmalonyl-CoA mutase
VNG0674C	VNG0674C	35.75	1	
VNG0675C	VNG0675C	46.31	1	
VNG0688H	VNG0688H	172.05	1	
VNG0689G	trp5	107.07	1	ABC transporter, ATP-binding protein
VNG0690C	VNG0690C	89.09	1	
VNG0692C	VNG0692C	89.17	1	
VNG0700G	yvgX	158.99	1	Copper (Cu) transporting P1-type ATPase
VNG0704C	VNG0704C	77.15	1	putative transcription regulator
VNG0705C	VNG0705C	132.02	1	
VNG0708H	VNG0708H	38.30	1	
VNG0709C	VNG0709C	9.13	0	
VNG0711C	VNG0711C	9.74	0	Putative thioredoxin
VNG0716G	afsQ2	58.74	1	Sensory histidine protein kinase homolog
VNG0723G	pepQ1	38.47	1	Probable peptidase
VNG0725H	VNG0725H	115.41	1	
VNG0730C	VNG0730C	64.47	1	Putative 2-dehydropantoate 2-reductase
VNG0732G	tpc	79.39	1	Probable RNA 3'-terminal phosphate cyclase
VNG0734G	tfbB	184.64	1	Transcription initiation factor IIB 2 (TFIIB 2)
VNG0736G	kinA2	112.38	1	Signal-transducing histidine kinase homolog
VNG0737H	VNG0737H	113.49	1	
VNG0742H	VNG0742H	111.29	1	
VNG0745G	spoVR	129.93	1	Spore cortex synthesis protein
VNG0746C	VNG0746C	111.94	1	
VNG0748G	prkA	121.97	1	putative kinase
VNG0749G	prk	89.99	1	Protein kinase
VNG0750C	VNG0750C	132.23	1	putative GAF domain-containing protein (PF1590)
VNG0751C	VNG0751C	49.15	1	putative transcription regulator (PadR family)
VNG0755C	VNG0755C	11.61	0	
VNG0757G	tfeA	1.18	0	Transcription initiation factor IIE alpha subunit
VNG0764C	VNG0764C	9.16	0	NAD kinase, ATP-NAD kinase PF01513.

ORF Name	Gene Symbol	Estimate Fold Change	Significance by T-Test	Function
VNG0765H	VNG0765H	38.71	0	
VNG0766G	prp2	123.81	1	Phosphate regulatory protein homolog
VNG0772H	VNG0772H	47.93	1	
VNG0775G	acd2	91.18	1	Acyl-CoA dehydrogenase
VNG0793G	htr6	41.72	1	Htr6 transducer
VNG0794G	yufN	86.64	1	ABC transporter (Lipoprotein)
VNG0796G	cgs	15.06	1	Cystathionine gamma-synthase
VNG0798H	VNG0798H	92.36	1	
VNG0799C	VNG0799C	205.63	1	
VNG0800H	VNG0800H	130.54	1	
VNG0801C	VNG0801C	62.26	1	twin-arginine protein translocation protein
VNG0804C	VNG0804C	56.21	1	
VNG0810H	VNG0810H	2.90	0	
VNG0811H	VNG0811H	110.44	1	
VNG0812G	htr18	102.16	1	Htr18 transducer
VNG0813G	potD	118.79	1	Spermidine/putrescine-binding protein
VNG0814C	VNG0814C	116.83	1	
VNG0816G	chi	105.48	1	Chitinase
VNG0818C	VNG0818C	164.41	1	putative chitinase
VNG0819C	VNG0819C	150.11	1	
VNG0822C	VNG0822C	20.98	1	
VNG0823G	gspE2	1.93	0	Type II secretion system protein
VNG0824G	gdb	96.80	1	Molybdopterin-guanine dinucleotide biosynthesis protein A
VNG0826C	dmsR	124.17	1	putative transcription regulator
VNG0830G	dmsB	10.38	1	Molybdopterin oxidoreductase
VNG0831G	dmsC	17.94	1	Molybdopterin oxidoreductase
VNG0832C	dmsD	13.05	1	Protein induced by anaerobic growth on TMAO and/or DMSO
VNG0836H	VNG0836H	70.00	1	
VNG0837H	VNG0837H	87.72	1	
VNG0838G	ssrA	51.96	1	Integrase/recombinase
VNG0840H	VNG0840H	60.14	1	
VNG0841G	icfA	111.56	1	Carbonic anhydrase
VNG0845C	VNG0845C	14.68	1	putative methyltransferase family protein
VNG0849C	VNG0849C	3.42	0	
VNG0852C	VNG0852C	66.55	1	putative transcription regulator

ORF Name	Gene Symbol	Estimate Fold Change	Significance by T-Test	Function
VNG0853C	VNG0853C	120.00	1	
VNG0858C	VNG0858C	75.01	1	
VNG0869G	tfbD	78.95	1	Transcription initiation factor IIB 4 (TFIIB 4)
VNG0870G	gatC	65.81	1	Glutamyl-tRNA(Gln) amidotransferase subunit C
VNG0875C	VNG0875Cm	71.22	1	M50 family peptidase (metalloprotease)
VNG0883H	VNG0883H	48.17	1	puatative metallo-B-lactamase family protein
VNG0906H	VNG0906H	21.36	1	
VNG0915G	hakA	66.44	1	Atrazine chlorohydrolase
VNG0916G	ark	84.48	1	Adaptive-response sensory-kinase
VNG0919G	gst	21.17	1	Galactosyltransferase homolog
VNG0926H	VNG0926H	51.63	1	
VNG0928G	mak	17.47	1	MAPK-activated protein kinase
VNG0930G	yvbT	30.48	1	Alkanal monooxygenase homolog
VNG0933G	yqjM	26.15	1	NADH-dependent flavin oxidoreductase
VNG0935G	noxC	71.31	1	NADH oxidase
VNG0938G	gufA	13.48	1	putative divalent cation transporter
VNG0946G	minD1	15.19	0	Cell division inhibitor
VNG0947G	fapJ	99.26	1	Flagella accessory protein J
VNG0960G	flaB1	35.86	1	Flagellin B1 precursor
VNG0961G	flaB2	16.35	1	Flagellin B2 precursor
VNG0962G	flaB3	26.07	1	Flagellin B3 precursor
VNG0963G	cpcE	7.14	0	Phycocyanin alpha phycocyanobilin lyase-like
VNG0969H	VNG0969H	31.36	1	
VNG0978H	VNG0978H	25.31	1	
VNG0979H	VNG0979H	120.73	1	
VNG0988H	VNG0988H	31.30	1	
VNG0991H	VNG0991H	107.89	1	
VNG0992H	VNG0992H	201.29	1	
VNG0995H	VNG0995H	51.66	1	
VNG0997G	acs2	43.20	1	Acetyl-CoA synthetase
VNG0999H	VNG0999H	81.46	1	
VNG1012H	VNG1012H	85.00	1	glutaredoxin
VNG1013G	htr13	96.34	1	Htr13 transducer
VNG1015H	VNG1015H	83.18	1	
VNG1017H	VNG1017H	119.67	1	

ORF Name	Gene Symbol	Estimate Fold Change	Significance by T-Test	Function
VNG1018G	adh3	37.67	1	Alcohol dehydrogenase
VNG1020C	VNG1020C	24.82	1	
VNG1023C	VNG1023C	58.09	1	Zn-binding alcohol dehydrogenase
VNG1024C	VNG1024C	97.70	1	6-pyruvoyl-tetrahydropterin synthase
VNG1025H	VNG1025H	75.04	1	
VNG1026H	VNG1026H	54.04	1	
VNG1033G	hisC1	20.89	1	Histidinol-phosphate aminotransferase
VNG1035C	VNG1035C	21.91	1	putative choline dehydrogenase flavoprotein/oxidoreductase
VNG1038C	VNG1038C	12.80	1	
VNG1039H	VNG1039H	124.22	1	
VNG1041H	VNG1041H	61.85	1	
VNG1046H	VNG1046H	41.02	1	
VNG1047H	VNG1047H	129.31	1	
VNG1052H	VNG1052H	45.10	1	
VNG1059C	VNG1059C	44.90	1	
VNG1060H	VNG1060H	104.52	1	
VNG1063H	VNG1063H	84.26	1	
VNG1065C	VNG1065C	40.99	1	
VNG1073G	lfl1	37.21	1	Long-chain fatty-acid-CoA ligase
VNG1074G	ykfB2	28.23	1	Chloromuconate cycloisomerase
VNG1075G	menA	30.20	1	UbiA prenyltransferase, Involved in menaquinone biosynthesis
VNG1081G	menD	61.50	1	2-succinyl-6-hydroxy-2, 4-cyclohexadiene-1-carboxylate synthase
VNG1085H	VNG1085H	115.78	1	
VNG1087C	VNG1087C	20.55	1	
VNG1088C	VNG1088C	18.62	1	
VNG1092C	VNG1092C	66.24	1	
VNG1093C	VNG1093C	233.96	1	
VNG1094H	VNG1094H	52.18	1	
VNG1121G	aspC2	171.83	1	Aspartate aminotransferase
VNG1144H	VNG1144H	105.42	1	
VNG1168C	VNG1168C	111.76	1	
VNG1174G	nop58	60.07	1	Archaeal nucleolar protein homolog
VNG1175G	phoR	110.26	1	PhoR protein homolog
VNG1180G	msrA	142.69	1	Peptide methionine sulfoxide reductase msrA
VNG1181G	flaA1b	138.88	1	Flagellin A1 precursor

ORF Name	Gene Symbol	Estimate Fold Change	Significance by T-Test	Function
VNG1183H	VNG1183H	18.05	1	
VNG1185G	pqqE	23.63	1	Coenzyme PQQ synthesis protein
VNG1189H	VNG1189H	12.88	0	
VNG1191G	acd3	104.59	1	Acyl-CoA dehydrogenase
VNG1193C	VNG1193C	28.71	1	
VNG1200H	VNG1200H	124.98	1	
VNG1207C	VNG1207C	44.05	1	HTH domain-containing protein
VNG1208G	hutU	121.92	1	Probable urocanate hydratase
VNG1209G	hutG	128.62	1	Probable formimidoylglutamase/arginase family protein
VNG1211G	hutI	133.96	1	Probable imidazolonepropionase
VNG1212G	hutH	99.78	1	Probable histidine ammonia-lyase
VNG1213C	VNG1213C	117.48	1	Archaeal DNA polymerase
VNG1232G	psc	46.22	1	Probable 3-phosphoshikimate 1-carboxyvinyltransferase
VNG1238C	VNG1238C	28.18	1	
VNG1240G	yhdG	44.17	1	Putative ornithine-arginine antiporter
VNG1251G	helA	168.25	1	ATP-dependent helicase
VNG1256G	ribG	128.99	1	Riboflavin-specific deaminase
VNG1261H	VNG1261H	7.40	0	
VNG1264C	VNG1264C	93.61	1	
VNG1266G	ogg	74.39	1	8-oxoguanine DNA glycosylase
VNG1271H	VNG1271H	52.35	1	
VNG1275G	hflX2	98.45	1	GTP-binding protein
VNG1285G	trh2	20.88	1	Transcription regulator
VNG1295H	VNG1295H	132.68	1	
VNG1296C	VNG1296C	198.08	1	putative adenylate cyclase
VNG1301G	cysK	198.69	1	Cysteine synthase
VNG1302H	VNG1302H	51.65	1	
VNG1311G	alkA	97.19	1	3-methyladenine DNA glycosylase
VNG1314H	VNG1314H	116.90	1	
VNG1315H	VNG1315H	132.17	1	
VNG1318H	VNG1318H	128.10	1	
VNG1324C	VNG1324C	243.41	1	
VNG1326H	VNG1326H	20.22	1	
VNG1332G	sod2	27.85	1	Superoxide dismutase [Mn] 2
VNG1335G	phr2	53.42	1	Deoxyribodipyrimidine photolyase

ORF Name	Gene Symbol	Estimate Fold Change	Significance by T-Test	Function
VNG1337C	VNG1337C	25.89	0	
VNG1342G	mer	264.81	1	N5,N10-methylenetetrahydromethanopterin reductase
VNG1353C	VNG1353C	124.38	1	
VNG1372C	VNG1372C	90.66	1	
VNG1375C	VNG1375C	56.51	1	
VNG1380H	VNG1380H	63.18	1	
VNG1381H	VNG1381H	42.22	1	
VNG1384H	VNG1384H	92.83	1	
VNG1385G	yvoF	117.24	1	Acetyltransferase homolog
VNG1387H	VNG1387H	67.62	1	
VNG1388H	VNG1388H	23.64	0	
VNG1395G	htr9	68.54	1	Halobacterial transducer protein III
VNG1404G	trh1	44.33	1	Transcription regulator
VNG1406G	rhl	2.70	0	putative DNA helicase
VNG1413H	VNG1413H	17.28	1	
VNG1418C	VNG1418C	100.32	1	
VNG1420H	VNG1420H	112.14	1	
VNG1423H	VNG1423H	46.35	1	
VNG1425H	VNG1425H	158.91	1	
VNG1426H	VNG1426H	11.62	1	putative transcription regulator (PadR family)
VNG1431C	VNG1431C	7.25	0	Dihydroorotate Dehydrogenase B
VNG1437G	serA2	65.96	1	Phosphoglycerate dehydrogenase
VNG1440H	VNG1440H	68.46	1	
VNG1446H	VNG1446H	70.33	1	
VNG1447H	VNG1447H	51.07	1	
VNG1453H	VNG1453H	91.46	1	
VNG1455H	VNG1455H	84.47	1	putative DoxD-like protein
VNG1457C	VNG1457C	45.35	1	putative acetyltransferase
VNG1458G	crtB1	146.86	1	Phytoene synthase
VNG1459H	VNG1459H	146.14	1	
VNG1463G	blp	135.48	1	Bacterio-opsin linked product
VNG1464G	bat	131.26	1	Bacterio-opsin activator
VNG1465G	brp	165.23	1	Bacteriorhodopsin related protein
VNG1467G	bop	122.23	1	Bacteriorhodopsin precursor (BR)
VNG1468H	VNG1468H	112.64	1	

ORF Name	Gene Symbol	Estimate Fold Change	Significance by T-Test	Function
VNG1474G	est	9.22	0	Carboxylesterase
VNG1484H	VNG1484H	33.90	1	
VNG1490H	VNG1490H	3.65	0	putative transcription regulator
VNG1493G	purF	12.96	0	Glutamine phosphoribosylpyrophosphate amidotransferase
VNG1497C	VNG1497C	32.59	1	
VNG1503C	VNG1503C	113.99	1	
VNG1514H	VNG1514H	53.68	1	
VNG1519H	VNG1519H	168.77	1	
VNG1523G	htr8	112.74	1	Htr8 transducer
VNG1529G	mmdA	40.95	1	Acetyl-CoA carboxylase, subunit alpha
VNG1536C	VNG1536C	55.77	1	Putative universal stress protein (UspA)
VNG1540G	ywfD	121.73	1	Glucose 1-dehydrogenase
VNG1546H	VNG1546H	86.13	1	
VNG1547C	VNG1547C	95.89	1	
VNG1565G	hmcA	3.57	0	Protoporphyrin IX magnesium chelatase
VNG1574G	cobA	71.97	1	Cobalamin adenosyltransferase
VNG1576G	cbiP	51.54	1	Cobyric acid synthase
VNG1583C	cbiZ	31.97	1	adenosylcobinamide (AdoCbi) amidohydrolase
VNG1589C	VNG1589C	125.58	1	
VNG1601G	gcvP2	4.35	0	Glycine dehydrogenase subunit 2
VNG1607G	cheC2	16.92	1	Chemotaxis protein
VNG1609C	VNG1609C	12.38	1	
VNG1619H	VNG1619H	88.17	1	
VNG1621H	VNG1621H	131.07	1	
VNG1625H	VNG1625H	119.62	1	
VNG1626C	VNG1626C	68.31	1	putative membrane protein.
VNG1630H	VNG1630H	87.24	1	
VNG1637G	hcpA	115.73	1	Halocyanin precursor-like
VNG1645H	VNG1645H	61.28	1	
VNG1646G	trpG1	25.19	1	Anthranilate synthase beta chain
VNG1647G	trpE1	40.72	1	Anthranilate synthase alpha chain
VNG1648G	trpF	104.61	1	N-(5'-phosphoribosyl)anthranilate isomerase
VNG1649G	trpD1	63.75	1	Phosphoribosyl transferase
VNG1650H	VNG1650H	87.35	1	
VNG1656H	VNG1656H	90.65	1	

ORF Name	Gene Symbol	Estimate Fold Change	Significance by T-Test	Function
VNG1658C	VNG1658C	27.39	1	
VNG1659G	htr1	70.33	1	Sensory rhodopsin I transducer (HTR-I)
VNG1660G	sop1	100.47	1	Sensory rhodopsin I (SR-I)
VNG1663C	VNG1663C	18.58	0	
VNG1666H	VNG1666H	83.02	1	
VNG1667G	cdc48c	95.52	1	CdcH protein
VNG1674H	VNG1674H	55.43	1	
VNG1675H	VNG1675H	101.81	1	
VNG1676G	gbp2	63.23	1	GTP-binding protein homolog
VNG1678H	VNG1678H	85.61	1	
VNG1680G	crtB2	60.37	1	Phytoene synthase
VNG1681C	VNG1681C	102.89	1	
VNG1686G	mch	3.41	0	N(5),N(10)-methenyltetrahydromethanopterin cyclohydrolase
VNG1723H	VNG1723H	93.60	1	
VNG1733G	htr17	172.36	1	Htr17 transducer
VNG1734H	VNG1734H	163.46	1	
VNG1735C	VNG1735C	77.22	1	putative molybdenum cofactor sulfurase
VNG1744H	VNG1744H	76.04	1	
VNG1746C	VNG1746C	140.08	1	
VNG1751H	VNG1751H	215.16	1	
VNG1754G	phr1	124.10	1	Photolyase/cryptochrome
VNG1755G	crtI2	227.50	1	Phytoene dehydrogenase
VNG1758H	VNG1758H	147.72	1	
VNG1759G	htr7	144.35	1	Halobacterial transducer protein V
VNG1760G	htr5	65.91	1	Halobacterial transducer protein IV
VNG1762G	proX	136.78	1	Putative ABC transporter
VNG1781C	uppS	53.38	1	putative undecaprenyl pyrophosphate synthase
VNG1783H	VNG1783H	68.15	1	
VNG1785G	panF	90.23	1	Pantothenate permease
VNG1788C	VNG1788C	111.15	1	
VNG1794C	VNG1794C	67.47	1	putative Staphylococcal nuclease homolog
VNG1796H	VNG1796H	95.61	1	
VNG1801G	hsp1	63.53	1	Small heat shock protein
VNG1802H	VNG1802H	44.89	1	
VNG1806H	VNG1806H	74.30	1	succinate dehydrogenase/fumarate reductase

ORF Name	Gene Symbol	Estimate Fold Change	Significance by T-Test	Function
VNG1811G	eye	81.39	1	Succinoglycan biosynthesis
VNG1815G	carA	7.86	0	Carbamoyl-phosphate synthase small chain
VNG1816G	trh3	27.56	1	Transcription regulator
VNG1821G	adh4	95.26	1	Alcohol dehydrogenase
VNG1822G	moaB	11.24	0	Molybdenum cofactor biosynthesis protein
VNG1823C	VNG1823C	46.33	1	
VNG1834G	hyrA	179.71	1	Putative peroxiredoxin.
VNG1837G	ykfB1	20.39	1	Chloromuconate cycloisomerase
VNG1838H	VNG1838H	106.49	1	
VNG1846C	VNG1846C	76.47	1	
VNG1848H	VNG1848H	57.83	1	ThiS like protein
VNG1852H	VNG1852H	53.96	1	
VNG1853G	eif2ba	96.06	1	Translation initiation factor eIF-2B subunit alpha
VNG1856G	htr3	101.09	1	Htr3 transducer
VNG1857C	VNG1857C	49.44	1	putative leucine binding protein
VNG1859G	deoC	9.62	0	Deoxyribose-phosphate aldolase
VNG1865H	VNG1865H	56.27	1	
VNG1872C	VNG1872C	92.19	1	Asparaginase
VNG1890H	VNG1890H	87.79	1	
VNG1891H	VNG1891H	62.59	1	
VNG1894C	VNG1894C	73.18	1	
VNG1903C	VNG1903Cm	28.98	1	predicted transcriptional regulator, exact copy of VNG1886C
VNG1904H	VNG1904H	143.02	1	
VNG1906H	VNG1906H	91.89	1	putative membrane protein of FxsA family
VNG1917H	VNG1917H	95.51	1	
VNG1919H	VNG1919H	199.01	1	Protein crcB homolog 1
VNG1921H	VNG1921H	26.08	1	Protein crcB homolog 2
VNG1922G	trh5	90.52	1	Transcription regulator
VNG1924G	trkA6	88.81	1	TRK potassium uptake system protein
VNG1925H	VNG1925H	79.06	1	
VNG1932G	nolA	10.67	0	NADH dehydrogenase/oxidoreductase-like protein
VNG1938C	VNG1938C	98.58	1	
VNG1942H	VNG1942H	45.15	1	
VNG1943H	VNG1943H	24.10	1	
VNG1945G	purL2	60.15	1	Phosphoribosylformylglycinamidine synthase I

ORF Name	Gene Symbol	Estimate Fold Change	Significance by T-Test	Function
VNG1946G	purU	92.45	1	Formyltetrahydrofolate deformylase
VNG1949G	gul2	138.63	1	Inosine-5'-monophosphate dehydrogenase-like
VNG1951G	sub	53.78	1	Subtilisin-like serine protease homolog
VNG1952H	VNG1952H	41.79	1	
VNG1962C	VNG1962C	75.30	1	ADP-Ribose Pyrophosphatase
VNG1963H	VNG1963H	34.89	1	
VNG1964H	VNG1964H	52.76	1	
VNG1971G	gpdB	48.87	1	anaerobic glycerol-3-phosphate dehydrogenase chain B
VNG1973H	VNG1973H	169.55	1	
VNG1974H	VNG1974H	54.58	1	
VNG1976H	VNG1976H	96.31	1	
VNG1977H	VNG1977H	112.56	1	
VNG1985C	VNG1985C	127.13	1	Na+/H+ antiporter family PF03553
VNG2001G	yjbG	74.22	1	Oligopeptidase
VNG2002H	VNG2002H	95.74	1	
VNG2006C	VNG2006C	145.05	1	member of the PP-loop superfamily (PF01171).
VNG2008H	VNG2008H	122.05	1	
VNG2012C	VNG2012C	105.94	1	
VNG2014H	VNG2014H	159.21	1	
VNG2024H	VNG2024H	104.34	1	
VNG2027H	VNG2027H	81.12	1	
VNG2028H	VNG2028H	55.48	1	
VNG2031G	nadE	139.79	1	NAD+ synthetase
VNG2034H	VNG2034H	122.86	1	
VNG2036G	hlx1	29.14	1	putative response regulator
VNG2037C	VNG2037C	45.37	1	putative sensory histidine kinase
VNG2059H	VNG2059H	4.44	0	
VNG2068C	VNG2068C	29.71	0	
VNG2073C	VNG2073C	70.29	1	
VNG2074H	VNG2074H	66.41	1	
VNG2081H	VNG2081H	74.78	1	
VNG2082G	polC	153.59	1	DNA polymerase bacteriophage-type
VNG2087G	hisH2	111.09	1	Imidazole glycerol phosphate synthase subunit hisH
VNG2091H	VNG2091H	60.14	1	putative phosphatase
VNG2101H	VNG2101H	137.72	1	

ORF Name	Gene Symbol	Estimate Fold Change	Significance by T-Test	Function
VNG2104G	pchB	29.64	1	Potassium channel homolog
VNG2109H	VNG2109H	43.66	1	
VNG2113C	VNG2113C	75.88	1	
VNG2115H	VNG2115H	114.00	1	glutaredoxin
VNG2123G	nhaC2	42.77	1	Na+/H+ antiporter
VNG2125G	ribA	23.42	1	GTP cyclohydrolase II
VNG2126C	VNG2126C	34.24	1	putative transcription regulator
VNG2130G	minD2	100.95	1	Cell division inhibitor
VNG2132G	fer1	106.98	1	Ferredoxin
VNG2136G	blh	111.82	1	Brp-like homolog
VNG2152C	VNG2152C	76.78	1	
VNG2154C	VNG2154C	35.71	1	
VNG2156C	VNG2156C	87.74	1	
VNG2159G	manC	67.78	1	Mannose-1-phosphate guanylyltransferase
VNG2162C	VNG2162C	6.18	0	
VNG2168C	VNG2168C	86.73	1	
VNG2176H	VNG2176H	68.01	1	
VNG2179H	VNG2179H	67.74	1	
VNG2184G	tfbA	215.58	1	Transcription initiation factor IIB 1 (TFIIB 1)
VNG2185H	VNG2185H	70.84	1	
VNG2191H	VNG2191H	9.78	0	
VNG2214G		96.56	1	multidrug efflux pump like protein
VNG2238C	VNG2238C	113.60	1	
VNG2246H	VNG2246H	69.15	1	
VNG2259C	VNG2259C	57.04	1	
VNG2260H	VNG2260H	70.35	1	
VNG2262H	VNG2262H	45.07	1	
VNG2271G	orc6	70.67	1	Orc / cell division control protein 6
VNG2274C	VNG2274C	31.29	1	predicted nuclease
VNG2281C	VNG2281C	37.15	1	
VNG2285C	VNG2285C	55.03	1	Putative signal peptidase
VNG2286G	mamA	55.11	1	vitamin B12 (cobalamin) binding domain-containing protein.
VNG2288G	mamB	68.32	1	Methylaspartate mutase
VNG2290G	maoC1	47.32	1	Monoamine oxidase regulatory-like
VNG2292H	VNG2292H	56.13	1	

ORF Name	Gene Symbol	Estimate Fold Change	Significance by T-Test	Function
VNG2294G	hisA	71.84	1	
VNG2296C	VNG2296C	42.58	1	
VNG2298H	VNG2298H	85.48	1	
VNG2308G	hlp	57.78	1	Hemolysin protein
VNG2310H	VNG2310H	68.50	1	
VNG2311H	VNG2311H	104.10	1	
VNG2315H	VNG2315H	37.26	1	
VNG2328H	VNG2328H	49.33	1	
VNG2329C	VNG2329C	46.98	1	
VNG2334C	VNG2334C	73.89	1	oxygen and light sensing response regulatory kinase
VNG2335H	VNG2335H	146.08	1	
VNG2338G	polA2	30.48	1	DNA polymerase II large subunit
VNG2340H	VNG2340H	67.45	1	
VNG2358G	appA	59.87	1	Oligopeptide binding protein
VNG2359G	appB	44.19	1	Oligopeptide ABC permease
VNG2361G	appC	57.95	1	Oligopeptide transport permease protein
VNG2363G	oppD1	52.20	1	Oligopeptide ABC transporter ATP-binding
VNG2365G	appF	68.63	1	Oligopeptide ABC transporter ATP-binding
VNG2366C	VNG2366C	45.99	1	
VNG2369C	VNG2369C	54.09	1	
VNG2373G	tnaA	67.71	1	Probable tryptophanase
VNG2374G	lysC	20.33	1	Aspartokinase II alpha subunit
VNG2376H	VNG2376H	77.48	1	
VNG2380H	VNG2380H	78.00	1	
VNG2385H	VNG2385H	85.73	1	
VNG2387H	VNG2387H	96.96	1	
VNG2392H	VNG2392H	57.36	1	
VNG2393G	tssA	19.34	1	Probable thiosulfate sulfurtransferase
VNG2394G	tssB	24.90	1	Thiosulfate sulfurtransferase
VNG2397G	cysA	100.97	1	Sulfate transport system ATP-binding protein
VNG2406C	VNG2406C	6.82	0	putative rubredoxin (Fe-containing electron transfer protein)
VNG2418G	aspC1	281.59	1	Aspartate aminotransferase
VNG2419C	VNG2419C	168.14	1	
VNG2420G	metA	122.11	1	Probable homoserine O-acetyltransferase
VNG2421G	hal	324.58	1	O-acetyl homoserine

ORF Name	Gene Symbol	Estimate Fold Change	Significance by T-Test	Function
VNG2422G	glcD	181.90	1	Glycolate oxidase subunit
VNG2423G	serB	92.76	1	Phosphoserine phosphatase
VNG2424G	serA1	42.39	1	Phosphoglycerate dehydrogenase
VNG2429G	dld	93.34	1	D-lactate dehydrogenase
VNG2430G	thrC1	133.33	1	Threonine synthase
VNG2431C	VNG2431C	68.55	1	
VNG2432C	VNG2432C	24.54	1	
VNG2433H	VNG2433H	155.73	1	
VNG2436G	argH	131.47	1	Argininosuccinate lyase
VNG2437G	argG	175.17	1	Argininosuccinate synthetase
VNG2442H	VNG2442H	4.20	0	
VNG2443G	dpsA	28.76	1	Starvation induced DNA binding protein
VNG2446H	VNG2446H	32.68	1	
VNG2447G	lta	184.18	1	L-allo-threonine aldolase
VNG2458C	VNG2458C	137.07	1	putative divalent cation transporter
VNG2461H	VNG2461H	58.36	1	
VNG2466C	VNG2466C	67.85	1	protein of unknown function
VNG2477H	VNG2477H	116.12	1	
VNG2480H	VNG2480Hm	131.69	1	Uncharacterized conserved protein
VNG2482G	pstB1	104.51	1	Phosphate ABC transporter ATP-binding
VNG2483G	pstA1	105.17	1	Phosphate ABC transporter permease
VNG2484G	pstC1	114.46	1	Phosphate transporter permease
VNG2486G	yqgG	99.08	1	Phosphate ABC transporter binding
VNG2488C	VNG2488C	218.76	1	
VNG2490H	VNG2490H	146.13	1	
VNG2493C	VNG2493C	92.10	1	
VNG2497H	VNG2497H	7.64	0	
VNG2498H	VNG2498H	95.08	1	
VNG2508C	VNG2508C	15.87	0	
VNG2511H	VNG2511H	145.76	1	
VNG2512G	epf1	68.72	1	mRNA 3'-end processing factor homolog
VNG2520C	VNG2520C	38.23	1	
VNG2521H	VNG2521H	89.57	1	
VNG2527G	dppD	22.23	0	Dipeptide ABC transporter ATP-binding
VNG2529G	dppB2	5.31	0	Dipeptide ABC transporter permease

ORF Name	Gene Symbol	Estimate Fold Change	Significance by T-Test	Function
VNG2532H	VNG2532H	11.55	1	
VNG2551G	fhuG	7.32	0	Ferrichrome ABC transporter permease
VNG2560G	yfmD2	1.71	0	Ferrichrome ABC transporter permease protein
VNG2563H	VNG2563H	30.69	1	
VNG2566H	VNG2566H	54.40	1	
VNG2569H	VNG2569H	170.34	1	
VNG2573G	hly	116.14	1	Halolysin
VNG2579G	idr1	123.84	1	Iron-dependent repressor homolog
VNG2593H	VNG2593H	135.23	1	
VNG2603H	VNG2603H	36.31	1	
VNG2610C	VNG2610C	141.17	1	
VNG2611G	hflX1	88.22	1	GTP-binding protein
VNG2617G	adh2	31.37	1	Alcohol dehydrogenase
VNG2619H	VNG2619H	96.67	1	
VNG2624G	ribC	30.25	1	Riboflavin synthase alpha subunit
VNG2626H	VNG2626H	77.67	1	
VNG2627C	VNG2627C	158.68	1	
VNG2631H	VNG2631H	130.46	1	
VNG2638G	bchP	38.22	1	putative flavoprotein
VNG2641H	VNG2641H	15.26	0	
VNG2645H	VNG2645H	61.96	1	
VNG2647G	vacB	61.71	1	Ribonuclease II family protein
VNG2656H	VNG2656H	93.83	1	
VNG2673H	VNG2673H	95.76	1	
VNG2674H	VNG2674H	124.42	1	
VNG2678H	VNG2678H	66.98	1	
VNG5001H	VNG5001H	118.77	1	
VNG5027G	gvpF1	26.49	1	GvpF protein, cluster A
VNG5028G	gvpE1	82.06	1	GvpE protein, cluster A
VNG5029G	gvpD1	94.86	1	GvpD protein, cluster A
VNG5030G	gvpA1	68.40	1	GvpA protein, cluster A
VNG5032G	gvpC1	90.72	1	GvpC protein, cluster A
VNG5033G	gvpN1	77.61	1	GvpN protein, cluster A
VNG5034G	gvpO1	43.18	1	GvpO protein, cluster A
VNG5035G	sojB	40.64	1	Spo0A activation inhibitor

ORF Name	Gene Symbol	Estimate Fold Change	Significance by T-Test	Function
VNG5038H	VNG5038H	31.10	1	
VNG5053H	VNG5053H	53.94	1	
VNG5055G	cydA	27.33	1	cytochrome d oxidase chain I
VNG5057G	cydB	25.88	1	cytochrome d oxidase chain II
VNG5062C	VNG5062C	131.39	1	
VNG5068G	boa3	65.51	1	bacterio-opsin activator-like protein
VNG5073H	VNG5073H	65.18	1	
VNG5079H	xerD	79.78	1	circular chromosome separation site-specific recombinase homolog
VNG5083H	VNG5083H	72.08	1	
VNG5098C	VNG5098C	94.61	1	
VNG5105H	VNG5105H	82.51	1	
VNG5118H	VNG5118H	116.04	1	
VNG5133C	VNG5133C	41.78	1	
VNG5141G	hepA	68.66	1	ATP-dependent RNA helicase
VNG5143C	VNG5143C	8.98	0	
VNG5145H	VNG5145H	99.81	1	likely involved in protein transport
VNG5150H	VNG5150H	66.49	1	
VNG5156H	VNG5156H	47.45	1	potential transcriptional regulator (repressor), COG1552
VNG5166H	VNG5166H	108.46	1	
VNG5175H	VNG5175H	56.30	1	
VNG5176C	arsR2	45.91	1	transcriptional regulator, arsR family, COG0640
VNG5177C	arsM	145.92	1	
VNG5185H	VNG5185H	99.07	1	
VNG6081G	crt	42.30	1	carotenoid biosynthetic protein
VNG6143H	VNG6143H	88.52	1	
VNG6144G	trsE	119.14	1	putative conjugation/plasmid transfer protein.
VNG6145H	VNG6145H	94.92	1	
VNG6149H	VNG6149H	99.57	1	
VNG6152H	VNG6152H	87.99	1	
VNG6157H	VNG6157H	65.77	1	
VNG6158H	VNG6158H	50.64	1	
VNG6160H	VNG6160H	80.15	1	
VNG6162H	VNG6162H	147.15	1	
VNG6163H	VNG6163H	50.28	1	
VNG6164G	orc2	62.43	1	Orc / cell division control protein 6

ORF Name	Gene Symbol	Estimate Fold Change	Significance by T-Test	Function
VNG6166H	VNG6166H	63.19	1	
VNG6168H	VNG6168H	55.70	1	
VNG6170H	VNG6170H	112.37	1	
VNG6175G	trkA2	97.77	1	TRK potassium uptake system protein
VNG6176G	kdpA	40.69	1	Potassium-transporting ATPase A chain
VNG6177G	kdpB	89.51	1	Potassium-transporting ATPase B chain
VNG6178G	kdpC	89.90	1	Potassium-transporting ATPase C chain
VNG6179G	cat3	145.74	1	Cationic amino acid transporter
VNG6183C	VNG6183C	71.58	1	
VNG6184G	cat4	87.23	1	Cationic amino acid transporter
VNG6185H	VNG6185H	98.63	1	
VNG6193H	VNG6193H	60.65	1	putative transcription regulator of the CopG family
VNG6195H	VNG6195H	140.37	1	putative RNA-binding protein
VNG6197H	VNG6197H	80.48	1	
VNG6198H	VNG6198H	102.70	1	
VNG6199G	cdc48d	143.00	1	Cell division cycle protein
VNG6201G	hsp5	73.97	1	Heat shock protease protein
VNG6203H	VNG6203H	55.55	1	
VNG6204H	VNG6204H	75.38	1	
VNG6206H	VNG6206H	151.80	1	
VNG6208C	VNG6208C	26.59	1	
VNG6210G	gabT	71.03	1	Gamma-aminobutyrate aminotransferase
VNG6211G	bdb	31.48	1	L-2,4-diaminobutyrate decarboxylase
VNG6212G	iucA	70.01	1	putative siderophore biosynthesis protein.
VNG6213G	iucB	45.63	1	Putative siderophore biosynthesis protein/acetyltransferase.
VNG6214G	hxyA	49.53	1	Monooxygenase putatively involved in siderophore biosynthesis
VNG6216G	iucC	41.40	1	putative siderophore biosynthesis protein
VNG6218G	trkA3	61.36	1	TRK potassium uptake system protein
VNG6225C	VNG6225C	47.89	1	
VNG6229G	gvpL2	93.35	1	GvpL protein 2
VNG6230G	gvpK2	160.41	1	GvpK protein 2
VNG6232G	gvpJ2	111.43	1	Gas vesicle protein gvpJ 2
VNG6233G	gvpI2	137.67	1	GvpI protein 2
VNG6235G	gvpH2	181.58	1	GvpH protein 2
VNG6236G	gvpG2	189.12	1	GvpG protein 2

ORF Name	Gene Symbol	Estimate Fold Change	Significance by T-Test	Function
VNG6237G	gvpF2	143.97	1	GvpF protein 2
VNG6239G	gvpE2	186.96	1	GvpE protein 2
VNG6240G	gvpD2	183.19	1	GvpD protein 2
VNG6241G	gvpA2	97.08	1	Gas vesicle structural protein 2 (GVP) (C-VAC)
VNG6242G	gvpC2	121.30	1	Gas vesicle protein C 2
VNG6244G	gvpN2	154.85	1	Gas vesicle protein gvpN 2
VNG6246G	gvpO2	106.77	1	GvpO protein 2
VNG6247G	trkA4	14.67	0	TRK potassium uptake system protein
VNG6250G	phoT3	43.15	1	Sodium-dependent phosphate transporter
VNG6251H	VNG6251H	10.68	0	
VNG6254C	VNG6254C	116.40	1	
VNG6258C	VNG6258C	43.03	1	putative cytosine/adenine deaminase, PFAM 1979
VNG6262G	zurM	245.42	1	ABC transporter, permease protein
VNG6264G	zurA	126.55	1	ABC transporter, ATP-binding protein
VNG6265G	ycdH	289.98	1	Adhesion protein
VNG6266H	VNG6266H	171.75	1	
VNG6275H	VNG6275H	74.15	1	
VNG6277G	ugpB	163.14	1	Glycerol-3-phosphate-binding protein precursor
VNG6279G	ugpA	73.46	1	Sn-glycerol-3-phosphate transport system permease
VNG6280G	ugpE	145.02	1	Sn-glycerol-3-phosphate transport system permease
VNG6281G	ugpC	146.42	1	Sn-glycerol-3-phosphate transport system ATP-binding
VNG6283H	VNG6283H	87.55	1	
VNG6284H	VNG6284H	65.37	1	
VNG6286H	VNG6286H	74.19	1	
VNG6288C	VNG6288C	110.32	1	putative transcription regulator of the CopG family
VNG6290H	VNG6290H	128.81	1	
VNG6293C	VNG6293C	75.17	1	
VNG6296C	VNG6296C	56.84	1	
VNG6301G	aph	167.69	1	Alkaline phosphatase
VNG6303G	exsB	57.01	0	Succinoglycan biosynthesis
VNG6313G	nhaC3	19.25	1	arcD, arginine/ornithine antiporter
VNG6318G	arcR	49.52	1	Transcription regulator, PFAM1614
VNG6320C	VNG6320C	24.44	0	
VNG6321H	VNG6321H	138.59	1	
VNG6323H	VNG6323H	83.91	1	

ORF Name	Gene Symbol	Estimate Fold Change	Significance by T-Test	Function
VNG6329H	VNG6329H	150.06	1	
VNG6330H	VNG6330H	85.64	1	
VNG6334H	VNG6334H	51.05	1	
VNG6335H	VNG6335H	83.78	1	
VNG6339H	VNG6339H	150.53	1	
VNG6340H	VNG6340H	57.72	1	
VNG6346H	VNG6346H	55.28	1	
VNG6348H	VNG6348H	139.23	1	
VNG6353H	VNG6353H	28.00	1	
VNG6354G	comA	84.70	1	Competence-like protein
VNG6364H	VNG6364H	57.70	1	
VNG6365H	VNG6365H	101.33	1	
VNG6366H	VNG6366H	77.22	1	
VNG6368H	VNG6368H	14.75	1	
VNG6370H	VNG6370H	13.10	1	
VNG6371G	mrr	47.92	1	Mrr restriction system protein homolog
VNG6373G	phrH	29.59	1	PhiH1 repressor homolog
VNG6375H	VNG6375H	119.48	1	
VNG6377H	VNG6377H	23.35	1	
VNG6378H	VNG6378H	116.79	1	
VNG6385H	VNG6385H	5.34	0	
VNG6389G	tfbE	62.28	1	Transcription initiation factor IIB 5 (TFIIB 5)
VNG6390H	VNG6390H	69.49	1	
VNG6400H	VNG6400H	37.21	1	
VNG6404H	VNG6404H	103.41	1	
VNG6407H	VNG6407H	79.27	1	
VNG6408G	phzF	17.43	1	Phenazine biosynthetic protein
VNG6412H	VNG6412H	87.76	1	
VNG6413H	VNG6413H	18.27	1	
VNG6416H	VNG6416H	116.91	1	
VNG6424H	VNG6424H	50.54	1	
VNG6427H	VNG6427H	75.70	1	
VNG6429H	VNG6429H	189.99	1	
VNG6430C	VNG6430C	45.48	1	
VNG6434H	VNG6434H	49.69	1	

ORF Name	Gene Symbol	Estimate Fold Change	Significance by T-Test	Function
VNG6438G	tbpF	34.35	1	TATA-box binding protein F (TATA-box factor F)
VNG6439H	VNG6439H	60.66	1	
