# Supplementary material for: Large scale physiological readjustment during growth enables rapid, comprehensive and inexpensive systems analysis
Source: BMC Syst Biol. 2010 May 14;4:64. doi: 10.1186/1752-0509-4-64 (PMC2880973; doi:10.1186/1752-0509-4-64)
Supplement: Additional file 6 — Functional KEGG pathway and ontology annotations for those genes reported in Additional tables S1 and S2. Functional assignments are as reported by KEGG, unmodified. [file 1752-0509-4-64-S6.DOC]

**Additional table S3. Overrepresented KEGG pathway annotations for the 471 stationary phase down regulated genes**

| **KEGG term** | **P-value** | **Count/Total** |
| --- | --- | --- |
| Ribosome | 0.00E+00 | 55/57 |
| Peptidoglycan biosynthesis | 0.00E+00 | 1/1 |
| Citrate cycle (TCA cycle) | 3.97E-03 | 14/18 |
| Oxidative phosphorylation | 5.90E-03 | 16/41 |
| ATP synthesis | 8.28E-03 | 8/9 |
| Reductive carboxylate cycle (CO2 fixation) | 9.46E-03 | 13/17 |
| RNA polymerase | 8.59E-02 | 9/12 |

**Additional table S4.** Overrepresented KEGG ontology annotations for the 471 stationary phase down regulated genes

| **KEGG term** | **P-value** | **Count/Total** |
| --- | --- | --- |
| Ribosome | 0.00E+00 | 55/60 |
| Pyruvate/Oxoglutarate oxidoreductases | 0.00E+00 | 4/4 |
| Protein folding and associated processing Environmental Information Processing | 0.00E+00 | 1/1 |
| Cell motility and secretion | 0.00E+00 | 1/1 |
| Inositol metabolism | 0.00E+00 | 1/1 |
| Glutathione metabolism | 0.00E+00 | 1/1 |
| Other nucleotide metabolism | 0.00E+00 | 1/1 |
| Glycan Biosynthesis and Metabolism | 0.00E+00 | 1/1 |
| Peptidoglycan biosynthesis | 0.00E+00 | 1/1 |
| mTOR signaling pathway | 0.00E+00 | 1/1 |
| Translation | 1.93E-14 | 81/129 |
| Genetic Information Processing | 4.13E-05 | 130/312 |
| Oxidative phosphorylation | 5.24E-04 | 26/41 |
| Citrate cycle (TCA cycle) | 1.03E-03 | 14/18 |
| Reductive carboxylate cycle (CO2 fixation) | 2.87E-03 | 13/17 |
| ATP synthesis | 4.43E-03 | 8/9 |
| Energy Metabolism | 5.13E-03 | 46/93 |
| RNA polymerase | 5.88E-03 | 11/14 |

**Additional table S5. Overrepresented KEGG pathway annotations for the 776 stationary phase upregulated genes**

| **KEGG term** | **P-value** | **Count/Total** |
| --- | --- | --- |
| Fluorene degradation | 0.00E+00 | 2/2 |
| Stilbene, coumarine and lignin biosynthesis | 0.00E+00 | 1/1 |
| Tyrosine metabolism | 1.44E-02 | 8/13 |
| ABC transporters - General | 2.73E-02 | 27/76 |

**Additional table S**6. Overrepresented KEGG ontology annotations for the 776 stationary phase upregulated genes

| **KEGG term** | **P-value** | **Count/Total** |
| --- | --- | --- |
| ATPases | 0.00E+00 | 1/1 |
| Signal transduction mechanisms | 0.00E+00 | 1/1 |
| Function unknown | 0.00E+00 | 1/1 |
| Diterpenoid biosynthesis | 0.00E+00 | 2/2 |
| Metabolism of xenobiotics by cytochrome P450 | 0.00E+00 | 2/2 |
| Styrene degradation | 0.00E+00 | 1/1 |
| Nitrobenzene degradation | 0.00E+00 | 1/1 |
| Fluorene degradation | 0.00E+00 | 2/2 |
| Carbazole degradation | 0.00E+00 | 1/1 |
| ATPases Environmental Information Processing | 0.00E+00 | 3/3 |
| Aminophosphonate metabolism | 0.00E+00 | 1/1 |
| Stilbene, coumarine and lignin biosynthesis | 0.00E+00 | 1/1 |
| 3-Chloroacrylic acid degradation | 0.00E+00 | 1/1 |
| 1,1,1-Trichloro-2,2-bis(4-chlorophenyl)ethane (DDT) degradation | 0.00E+00 | 1/1 |
| gamma-Hexachlorocyclohexane degradation | 0.00E+00 | 1/1 |
| Environmental Information Processing | 3.07E-02 | 44/136 |
| Tyrosine metabolism | 3.49E-02 | 8/13 |
| Membrane Transport | 9.49E-02 | 39/122 |
| ABC transporters | 1.54E-01 | 28/82 |
