# Supplementary material for: Large scale physiological readjustment during growth enables rapid, comprehensive and inexpensive systems analysis
Source: BMC Syst Biol. 2010 May 14;4:64. doi: 10.1186/1752-0509-4-64 (PMC2880973; doi:10.1186/1752-0509-4-64)
Supplement: Additional file 7 — Additional figure S3. Metabolites showing significant changes in abundance during growth. Each panel show a bar chart illustrating the changes in abundance for 51 detected metabolites whose levels change significantly during growth. The numbering scheme at the top of each panel does not have any practical significance and simply serves to identify each MS peak in the experiment. Histogram heights correspond to average counts for each metabolite in the triplicates. Measured levels are not indicative of absolute cellular concentrations. However, relative changes in concentrations between samples derived from different growth states may be inferred the histograms. [file 1752-0509-4-64-S7.PDF]

Halobacteria data

Differentially abundant early exponential, exponential and stationary phase metabolites

54 features

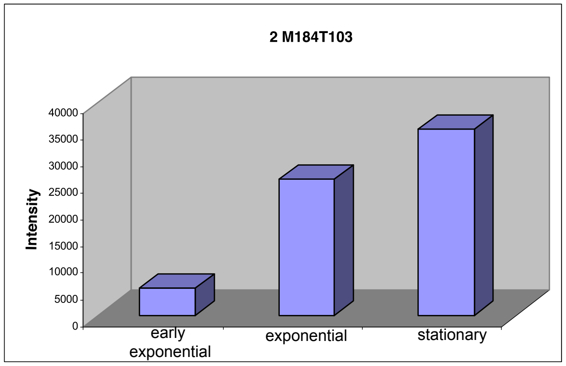
2 M184T103
Intensity
40000
35000
30000
25000
20000
15000
10000
5000
0
early exponential
exponential
stationary

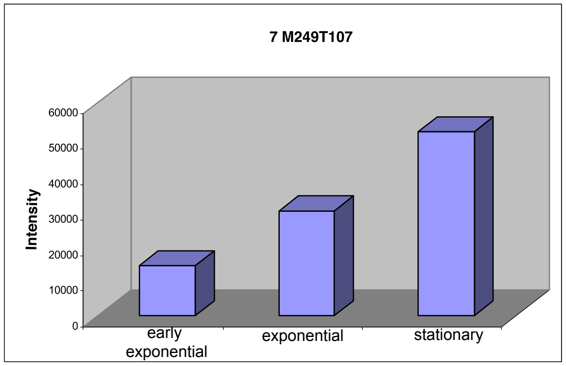
7 M249T107
Intensity
60000
50000
40000
30000
20000
10000
0
early exponential
exponential
stationary

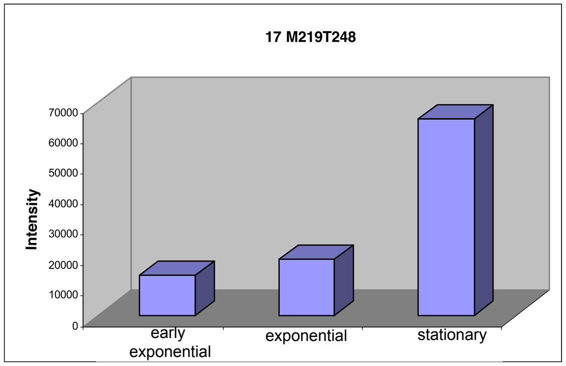
17 M219T248
Intensity
70000
60000
50000
40000
30000
20000
10000
0
early exponential
exponential
stationary

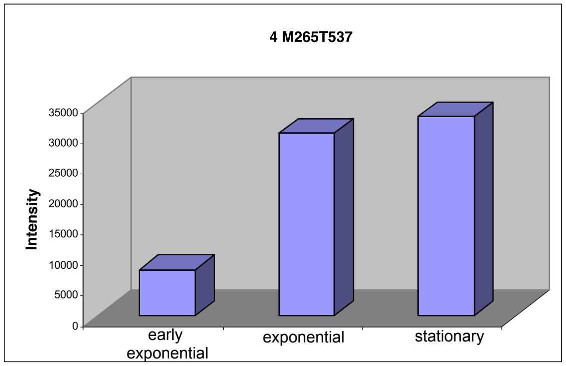
4 M265T537
Intensity
35000
30000
25000
20000
15000
10000
5000
0
early exponential
exponential
stationary

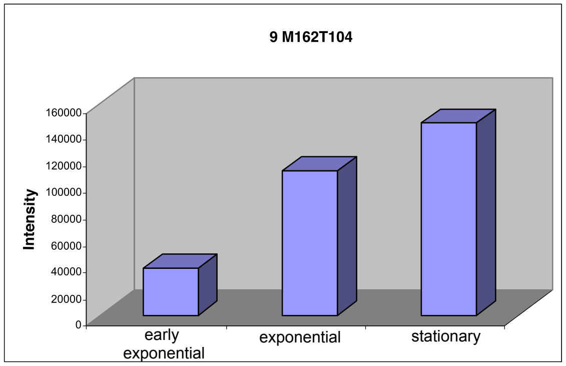
9 M162T104
Intensity
160000
140000
120000
100000
80000
60000
40000
20000
0
early exponential
exponential
stationary

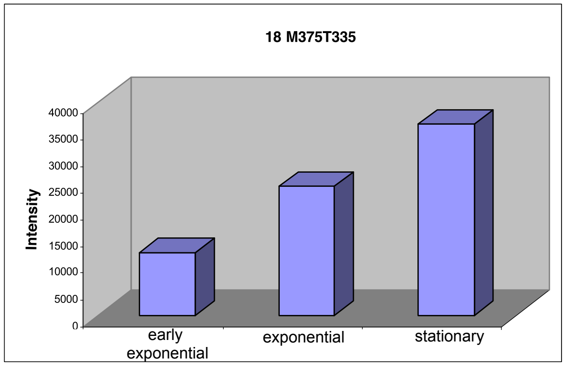
18 M375T335
Intensity
40000
35000
30000
25000
20000
15000
10000
5000
0
early exponential
exponential
stationary

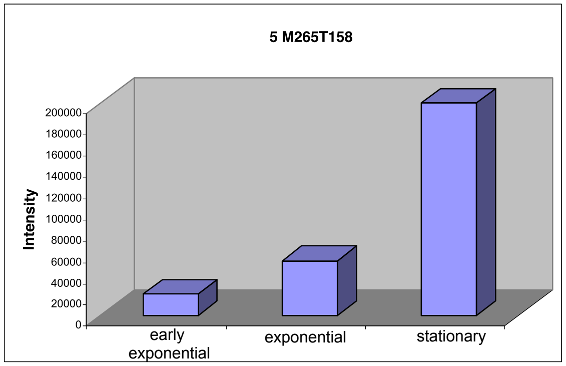
5 M265T158
Intensity
200000
180000
160000
140000
120000
100000
80000
60000
40000
20000
0
early exponential
exponential
stationary

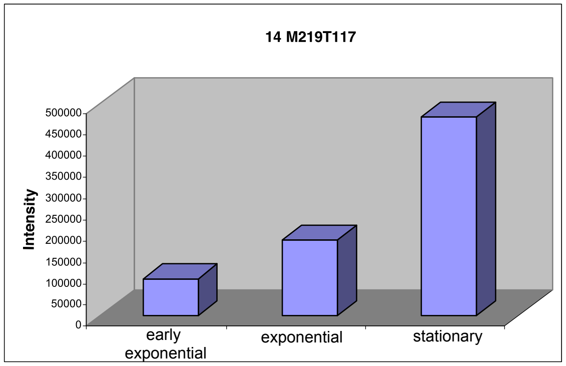
14 M219T117
Intensity
500000
450000
400000
350000
300000
250000
200000
150000
100000
50000
0
early exponential
exponential
stationary

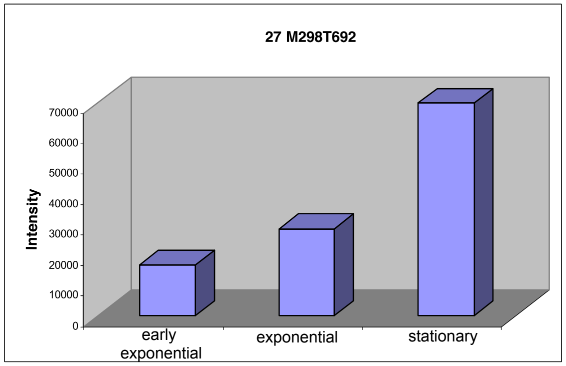
27 M298T692
Intensity
70000
60000
50000
40000
30000
20000
10000
0
early exponential
exponential
stationary

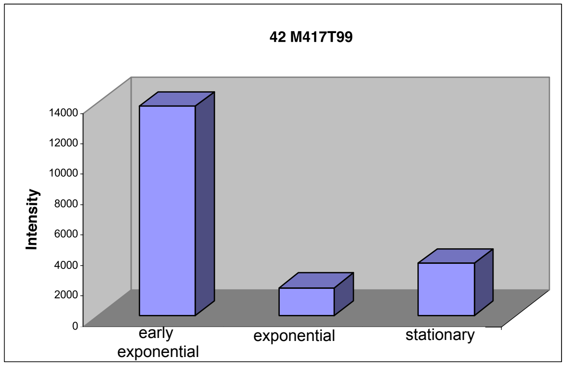
42 M417T99
Intensity
0
2000
4000
6000
8000
10000
12000
14000
early exponential
exponential
stationary

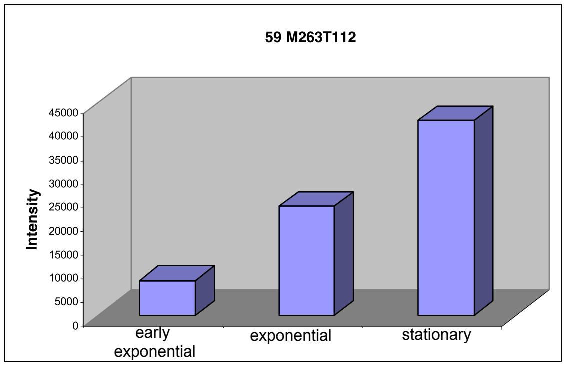
59 M263T112
Intensity
0
5000
10000
15000
20000
25000
30000
35000
40000
45000
early exponential
exponential
stationary

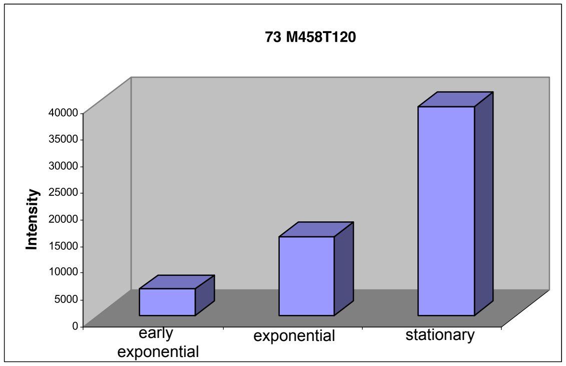
73 M458T120
Intensity
0
5000
10000
15000
20000
25000
30000
35000
40000
early exponential
exponential
stationary

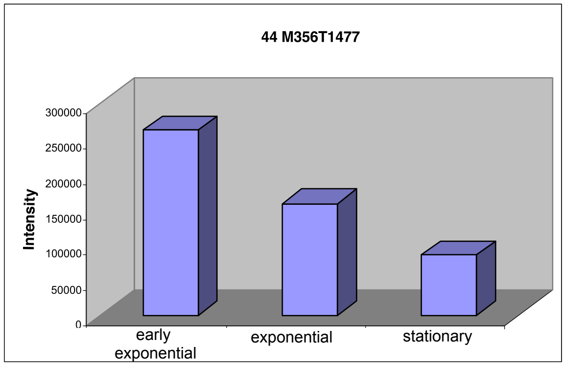
44 M356T1477
Intensity
0
50000
100000
150000
200000
250000
300000
early exponential
exponential
stationary

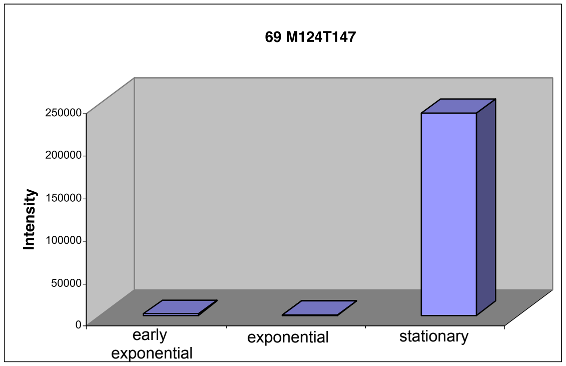
69 M124T147
Intensity
0
50000
100000
150000
200000
250000
early exponential
exponential
stationary

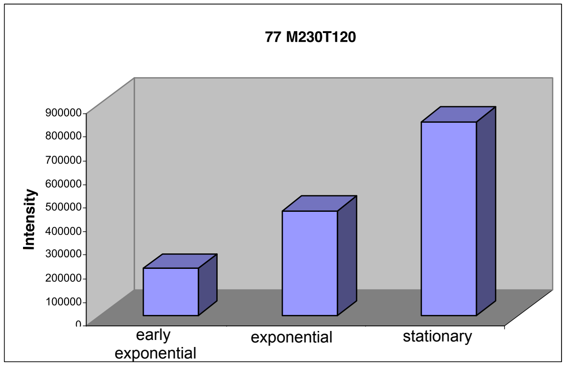
77 M230T120
Intensity
100000
200000
300000
400000
500000
600000
700000
800000
900000
early exponential
exponential
stationary

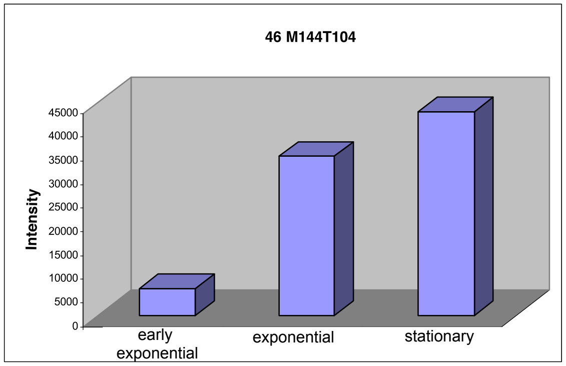
46 M144T104
Intensity
0
5000
10000
15000
20000
25000
30000
35000
40000
45000
early exponential
exponential
stationary

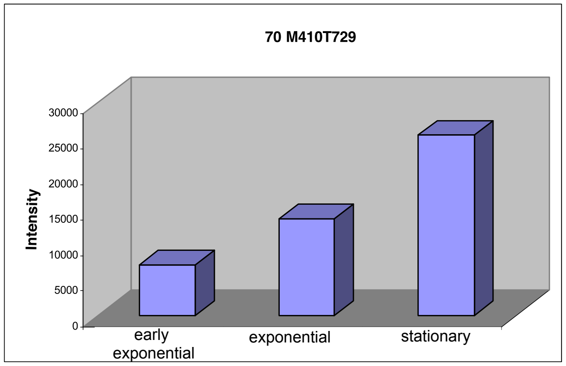
70 M410T729
Intensity
0
5000
10000
15000
20000
25000
30000
early exponential
exponential
stationary

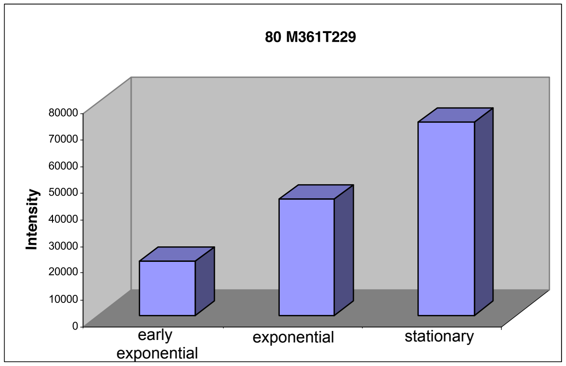
80 M361T229
Intensity
0
10000
20000
30000
40000
50000
60000
70000
80000
early exponential
exponential
stationary

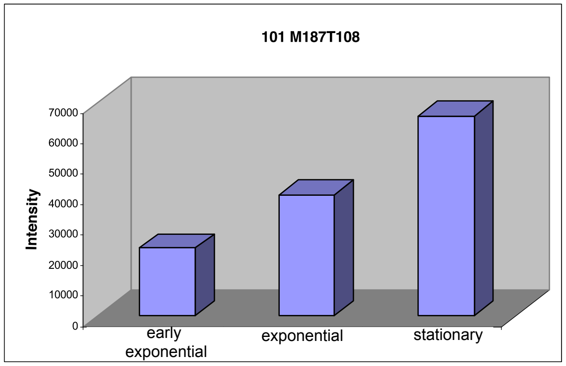
101 M187T108
Intensity
70000
60000
50000
40000
30000
20000
10000
0
early exponential
exponential
stationary

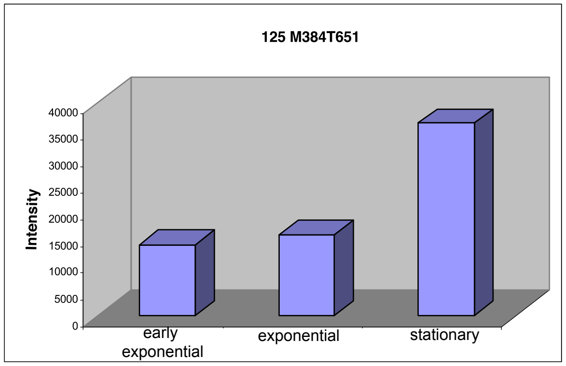
125 M384T651
Intensity
40000
35000
30000
25000
20000
15000
10000
5000
0
early exponential
exponential
stationary

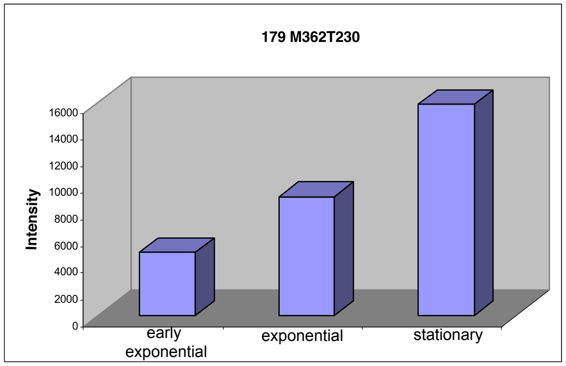
179 M362T230
Intensity
16000
14000
12000
10000
8000
6000
4000
2000
0
early exponential
exponential
stationary

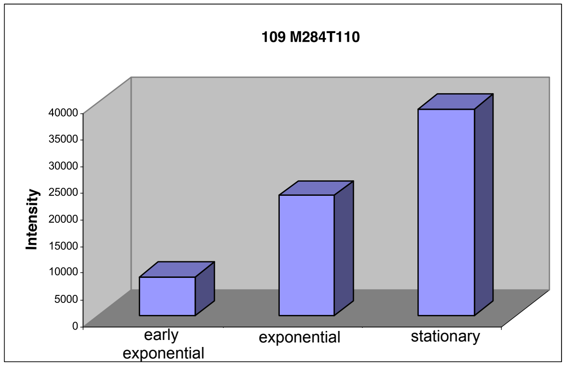
109 M284T110
Intensity
40000
35000
30000
25000
20000
15000
10000
5000
0
early exponential
exponential
stationary

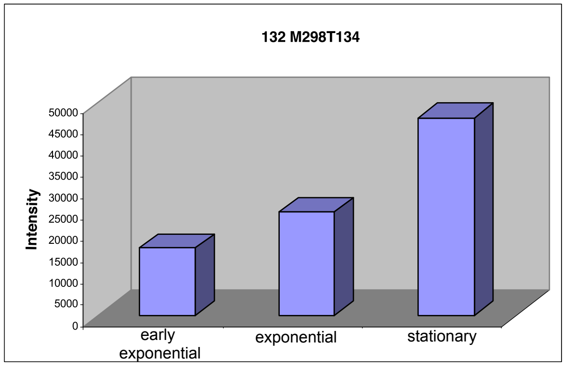
132 M298T134
Intensity
50000
45000
40000
35000
30000
25000
20000
15000
10000
5000
0
early exponential
exponential
stationary

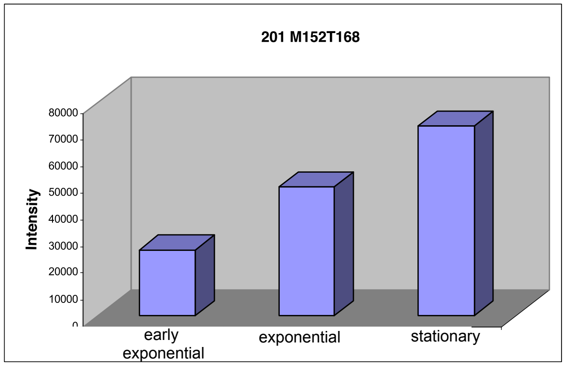
201 M152T168
Intensity
80000
70000
60000
50000
40000
30000
20000
10000
early exponential
exponential
stationary

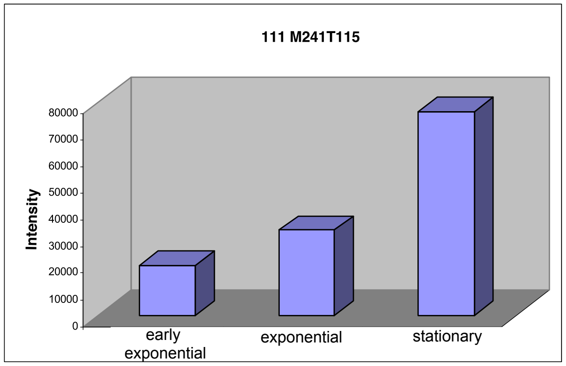
111 M241T115
Intensity
80000
70000
60000
50000
40000
30000
20000
10000
0
early exponential
exponential
stationary

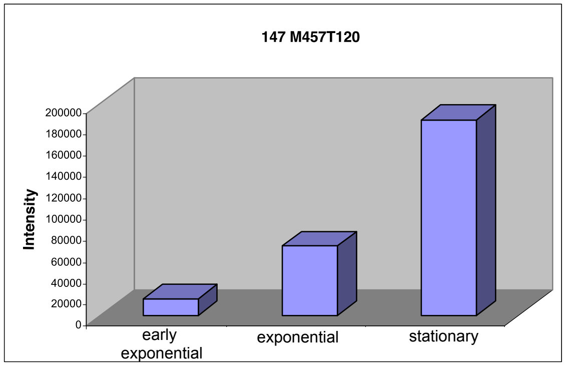
147 M457T120
Intensity
200000
180000
160000
140000
120000
100000
80000
60000
40000
20000
0
early exponential
exponential
stationary

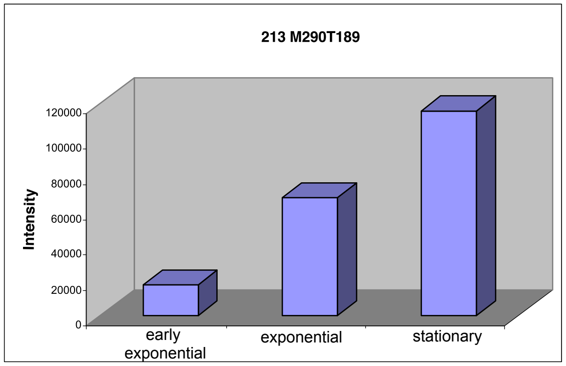
213 M290T189
Intensity
120000
100000
80000
60000
40000
20000
0
early exponential
exponential
stationary

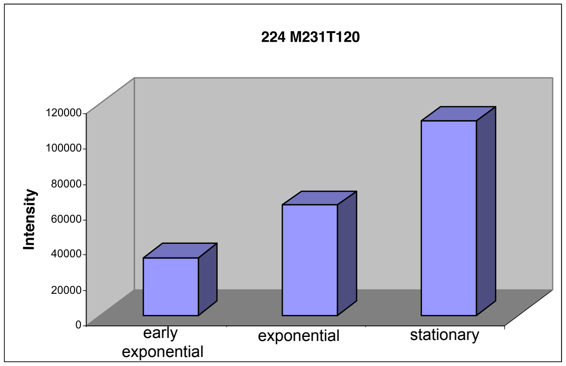
224 M231T120
Intensity
120000
100000
80000
60000
40000
20000
0
early exponential
exponential
stationary

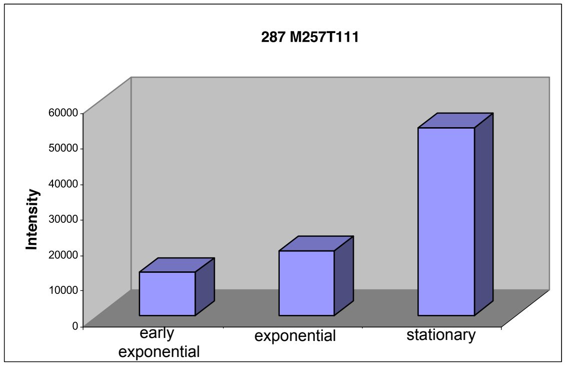
287 M257T111
Intensity
60000
50000
40000
30000
20000
10000
0
early exponential
exponential
stationary

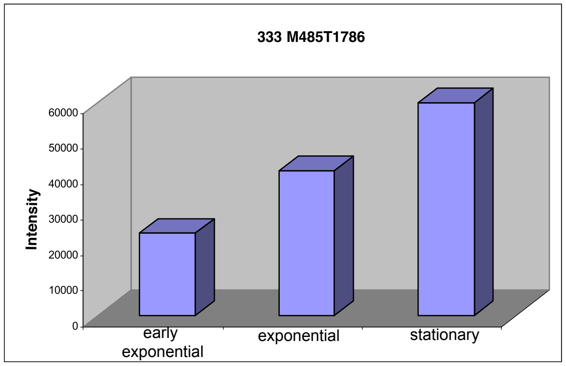
333 M485T1786
Intensity
60000
50000
40000
30000
20000
10000
0
early exponential
exponential
stationary

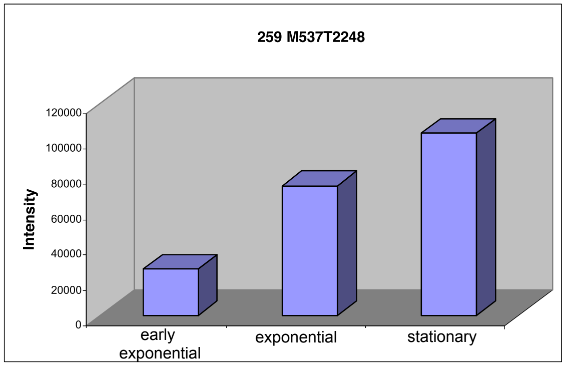
259 M537T2248
Intensity
120000
100000
80000
60000
40000
20000
0
early exponential
exponential
stationary

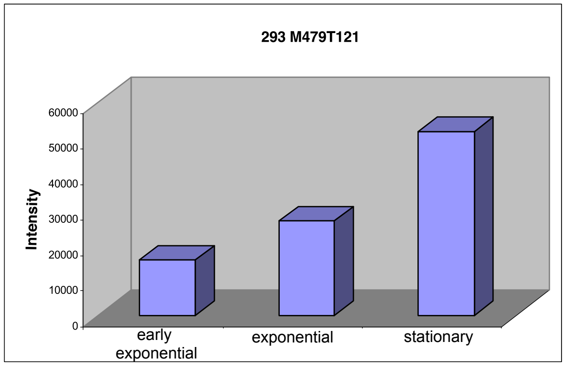
293 M479T121
Intensity
60000
50000
40000
30000
20000
10000
0
early exponential
exponential
stationary

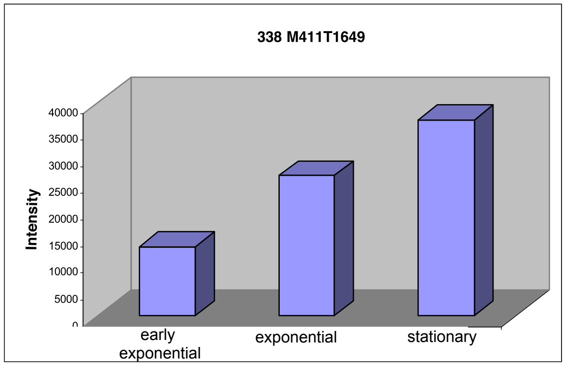
338 M411T1649
Intensity
40000
35000
30000
25000
20000
15000
10000
5000
early exponential
exponential
stationary

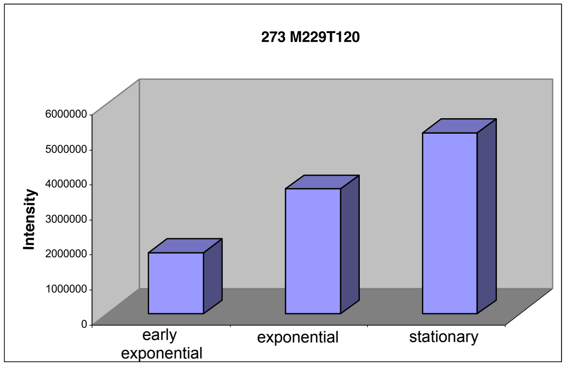
273 M229T120
Intensity
6000000
5000000
4000000
3000000
2000000
1000000
0
early exponential
exponential
stationary

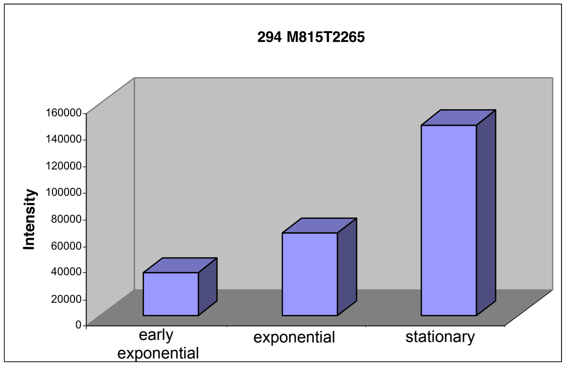
294 M815T2265
Intensity
160000
140000
120000
100000
80000
60000
40000
20000
0
early exponential
exponential
stationary

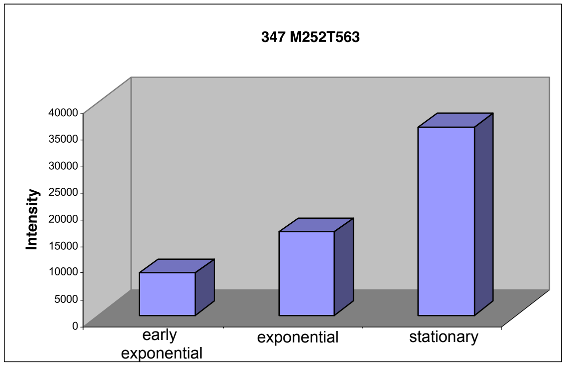
347 M252T563
Intensity
40000
35000
30000
25000
20000
15000
10000
5000
0
early exponential
exponential
stationary

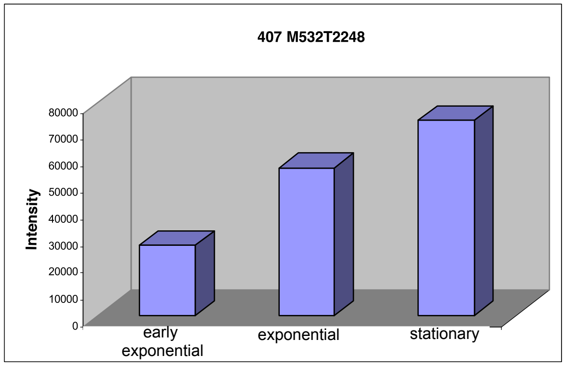
407 M532T2248
Intensity
80000
70000
60000
50000
40000
30000
20000
10000
0
early exponential
exponential
stationary

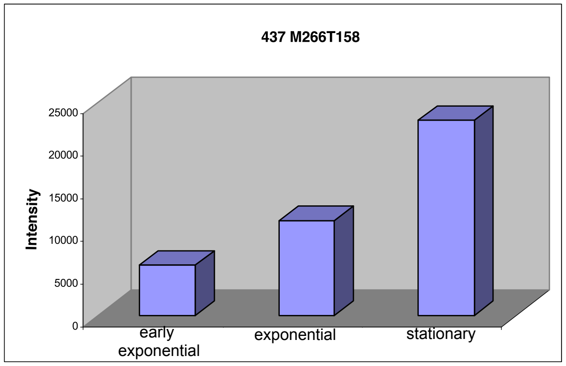
437 M266T158
Intensity
25000
20000
15000
10000
5000
0
early exponential
exponential
stationary

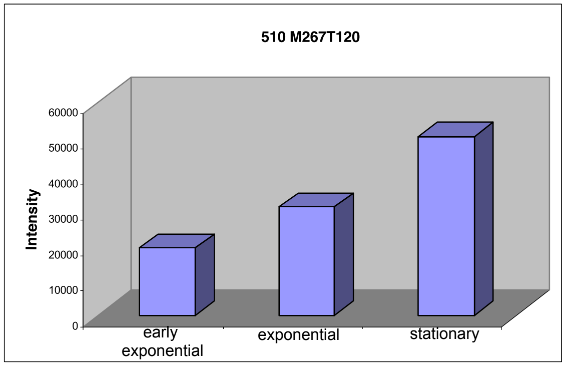
510 M267T120
Intensity
60000
50000
40000
30000
20000
10000
0
early exponential
exponential
stationary

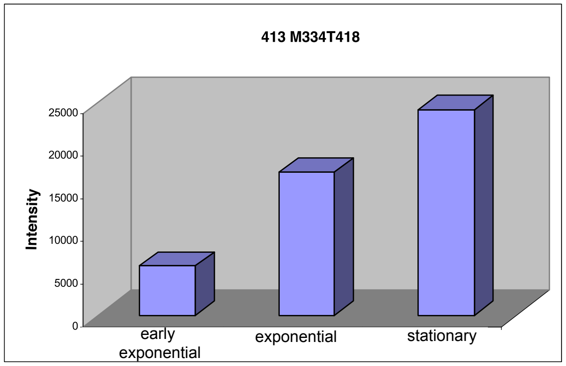
413 M334T418
Intensity
25000
20000
15000
10000
5000
0
early exponential
exponential
stationary

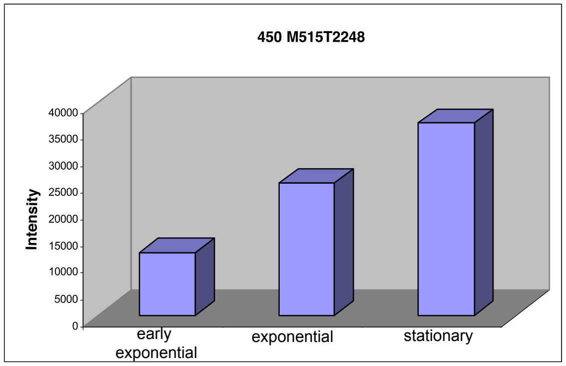
450 M515T2248
Intensity
40000
35000
30000
25000
20000
15000
10000
5000
0
early exponential
exponential
stationary

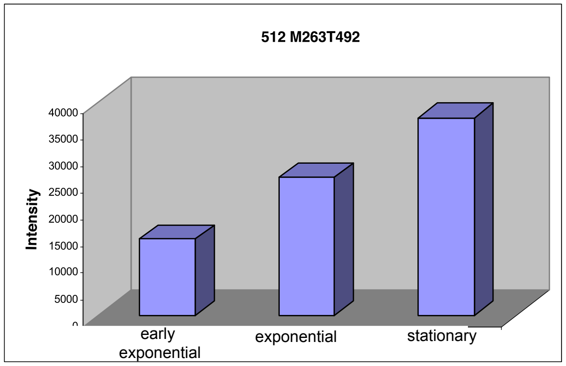
512 M263T492
Intensity
40000
35000
30000
25000
20000
15000
10000
5000
early exponential
exponential
stationary

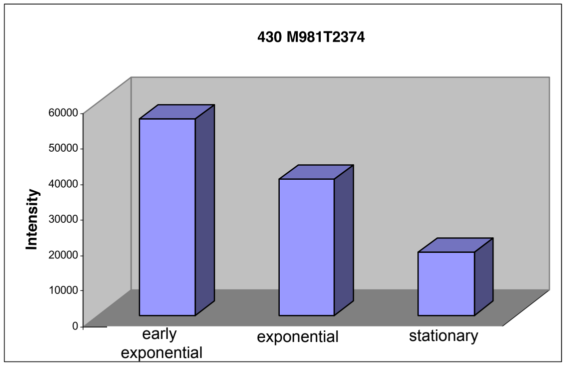
430 M981T2374
Intensity
60000
50000
40000
30000
20000
10000
0
early exponential
exponential
stationary

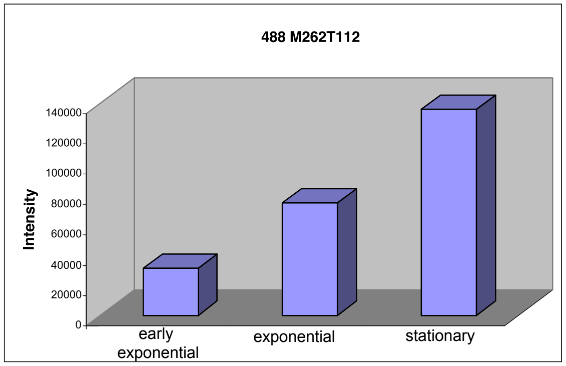
488 M262T112
Intensity
140000
120000
100000
80000
60000
40000
20000
0
early exponential
exponential
stationary

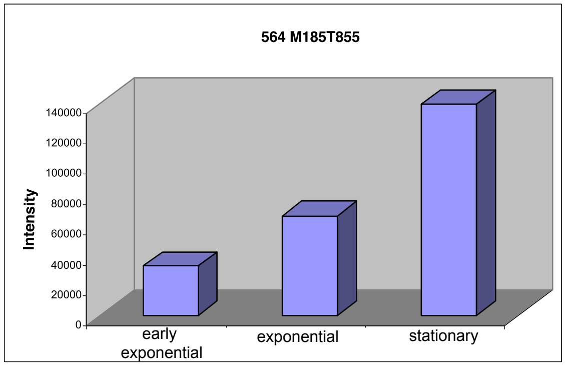
564 M185T855
Intensity
140000
120000
100000
80000
60000
40000
20000
0
early exponential
exponential
stationary

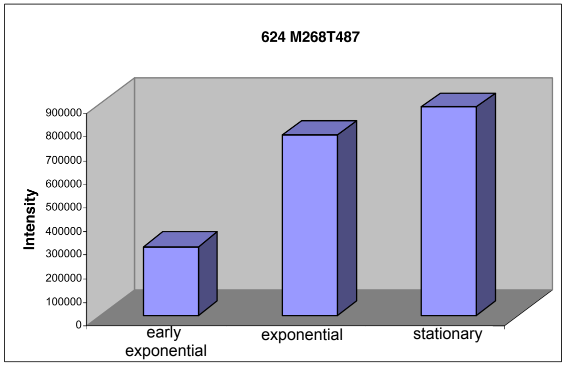
624 M268T487
Intensity
900000
800000
700000
600000
500000
400000
300000
200000
100000
0
early exponential
exponential
stationary

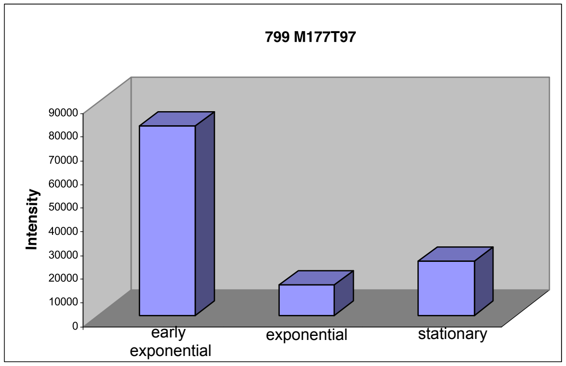
799 M177T97
Intensity
90000
80000
70000
60000
50000
40000
30000
20000
10000
0
early exponential
exponential
stationary

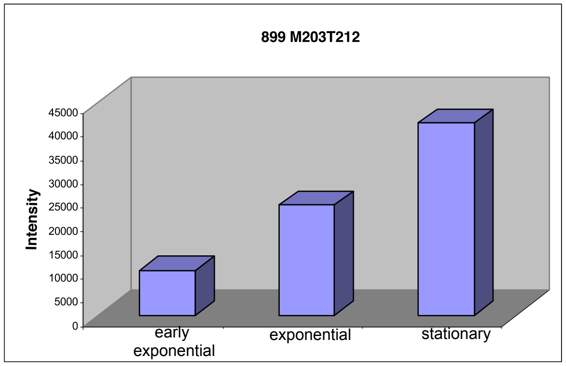
899 M203T212
Intensity
45000
40000
35000
30000
25000
20000
15000
10000
5000
0
early exponential
exponential
stationary

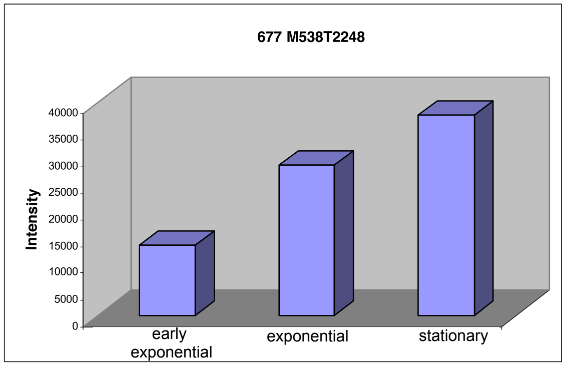
677 M538T2248
Intensity
40000
35000
30000
25000
20000
15000
10000
5000
0
early exponential
exponential
stationary

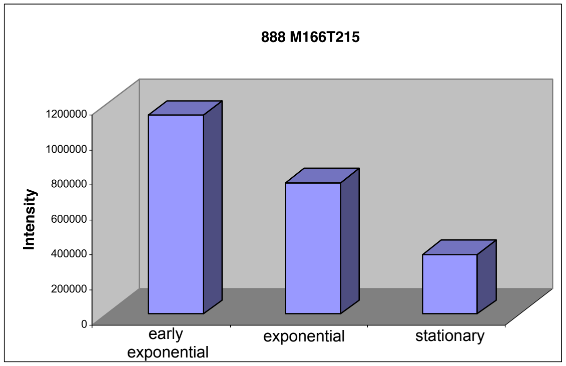
888 M166T215
Intensity
1200000
1000000
800000
600000
400000
200000
0
early exponential
exponential
stationary

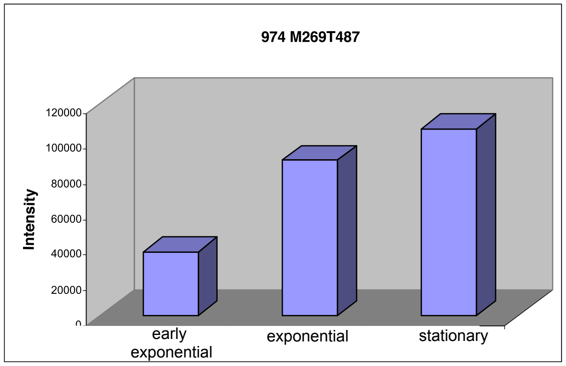
974 M269T487
Intensity
120000
100000
80000
60000
40000
20000
early exponential
exponential
stationary

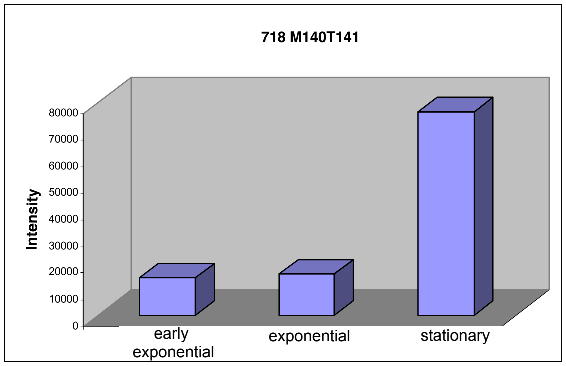
718 M140T141
Intensity
80000
70000
60000
50000
40000
30000
20000
10000
0
early exponential
exponential
stationary

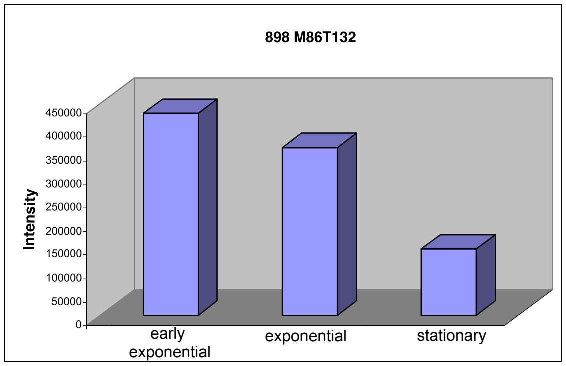
898 M86T132
Intensity
450000
400000
350000
300000
250000
200000
150000
100000
50000
0
early exponential
exponential
stationary

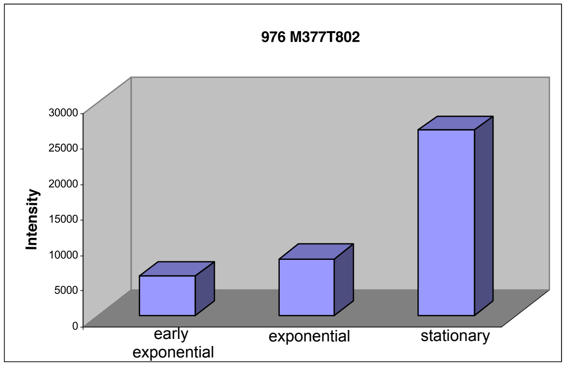
976 M377T802
Intensity
30000
25000
20000
15000
10000
5000
0
early exponential
exponential
stationary
