# Supplementary material for: Large scale physiological readjustment during growth enables rapid, comprehensive and inexpensive systems analysis
Source: BMC Syst Biol. 2010 May 14;4:64. doi: 10.1186/1752-0509-4-64 (PMC2880973; doi:10.1186/1752-0509-4-64)
Supplement: Additional file 8 — Additional figure S4. Spectra of citrulline, phenylalanine, riboflavin and 5-deoxyadenosine. Paired presentation of ion spectra measured from both H. salinarum NRC-1 and from purified metabolite standard for citrulline, phenylalanine, riboflavin and 5-deoxyadenosine. Relative abundance levels are also shown as a histogram. [file 1752-0509-4-64-S8.PDF]

Natural

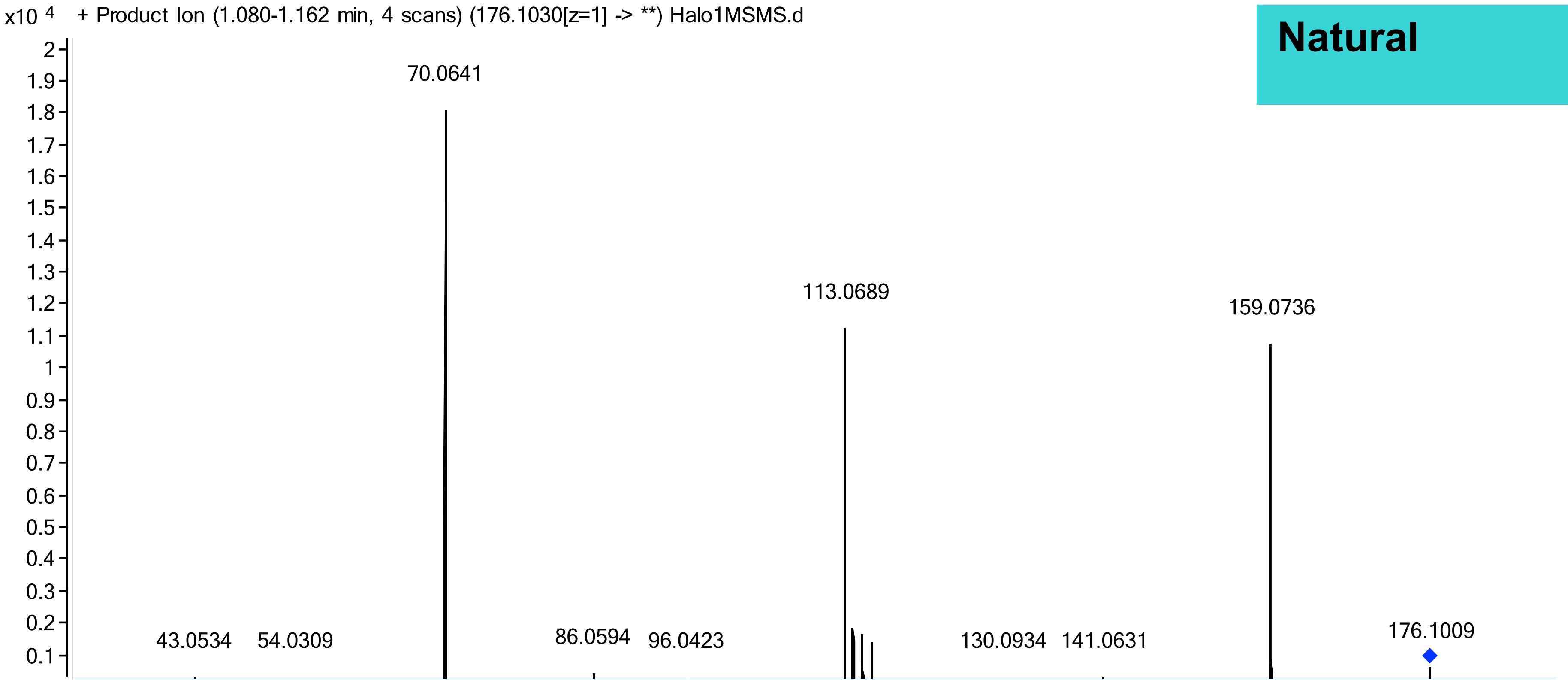

Standard

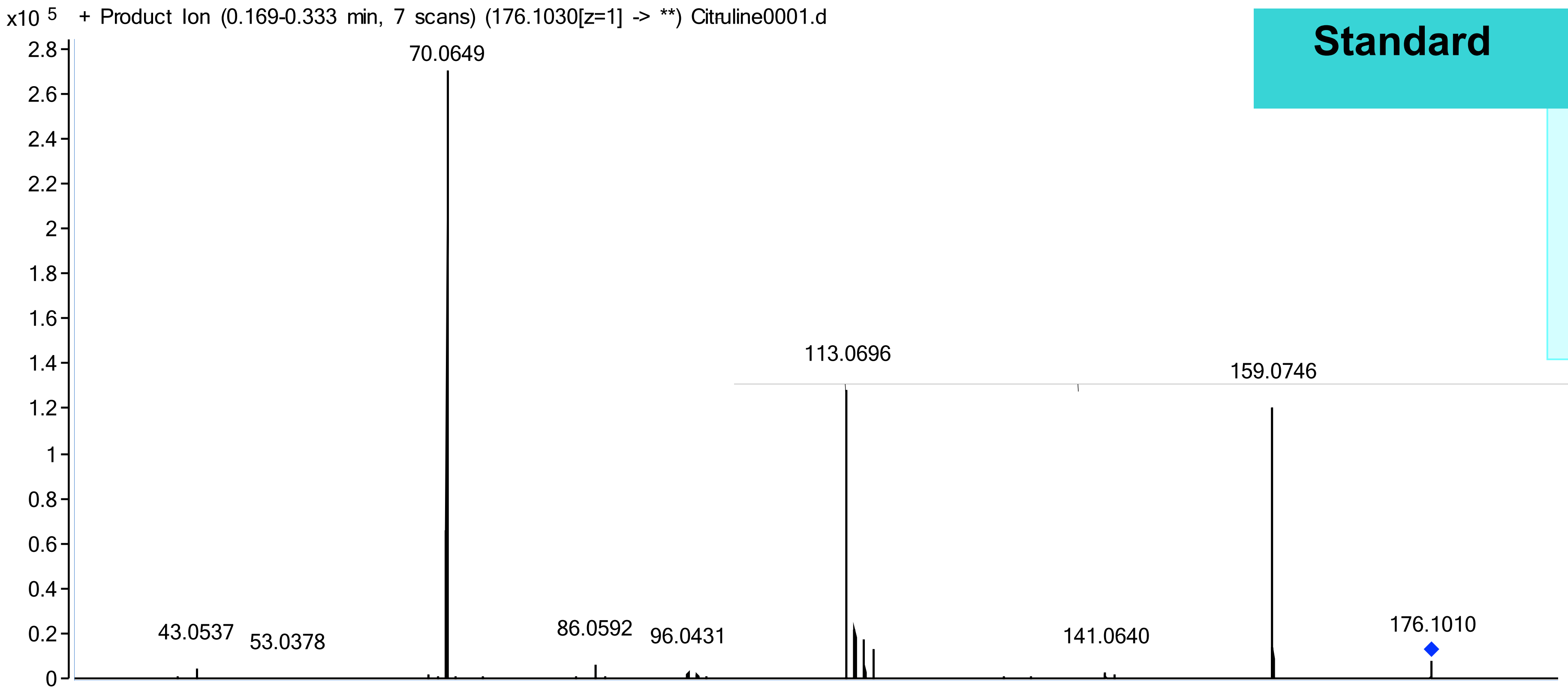

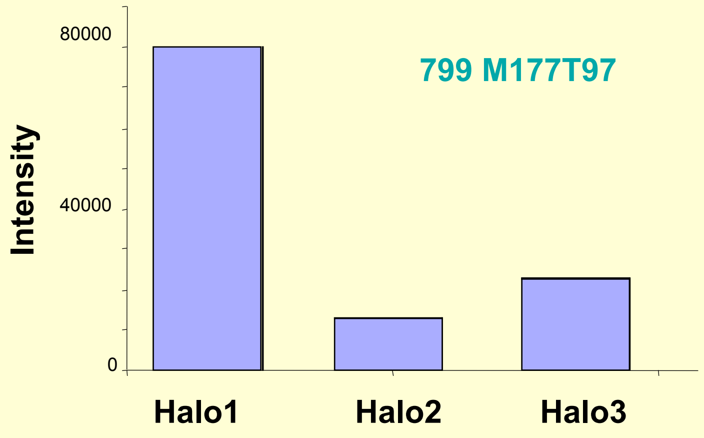

Retention time matches ~1.1 min

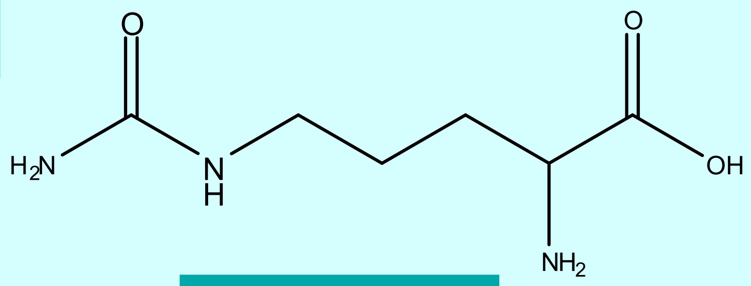

CITRULLINE

Formula:	$C_6H_{13}N_3O_3$
MM:	175.0957
MH+:	176.1030

Natural

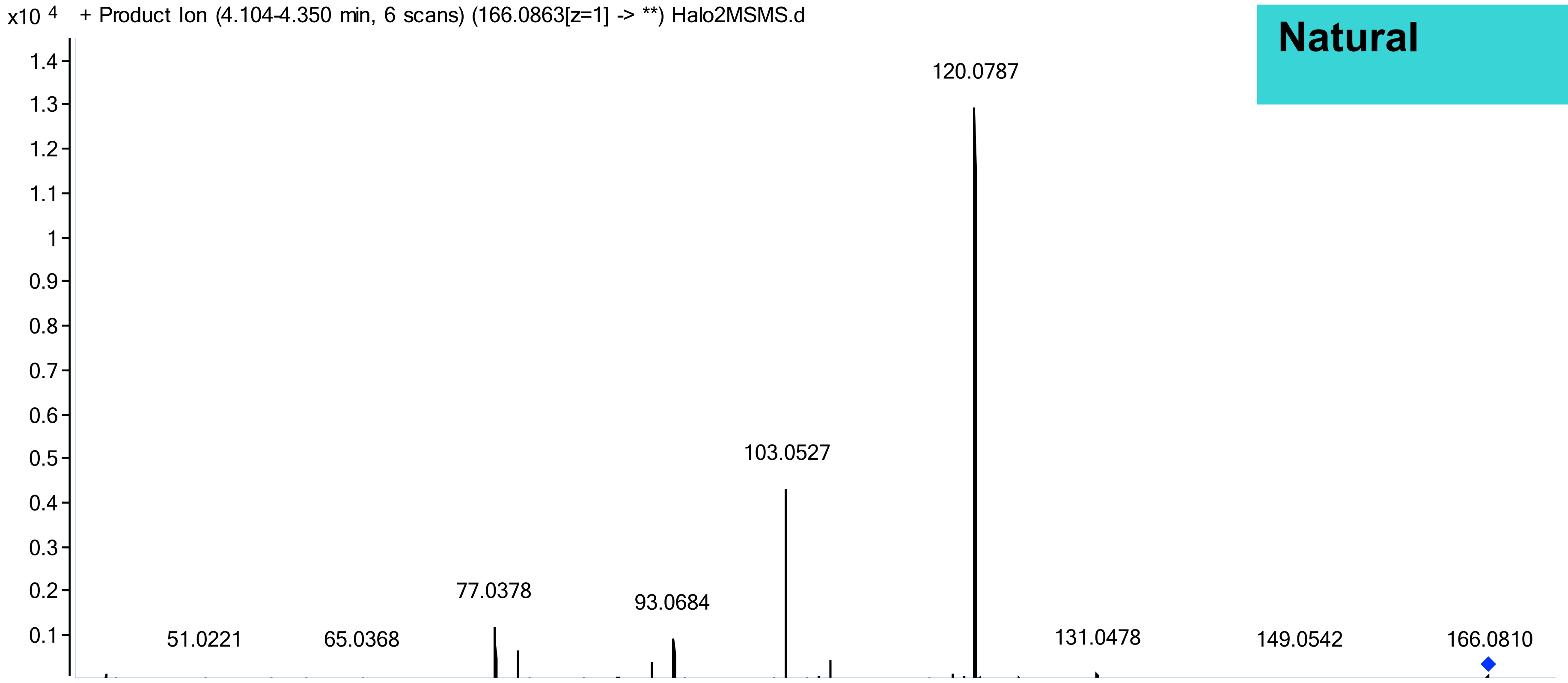

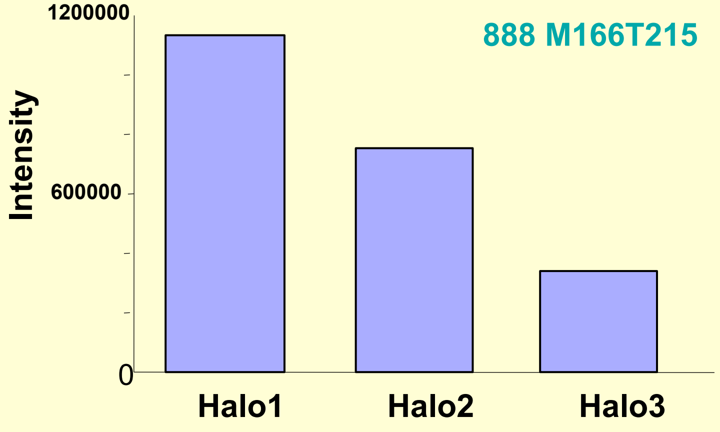

Retention time matches ~3.5 min

Standard

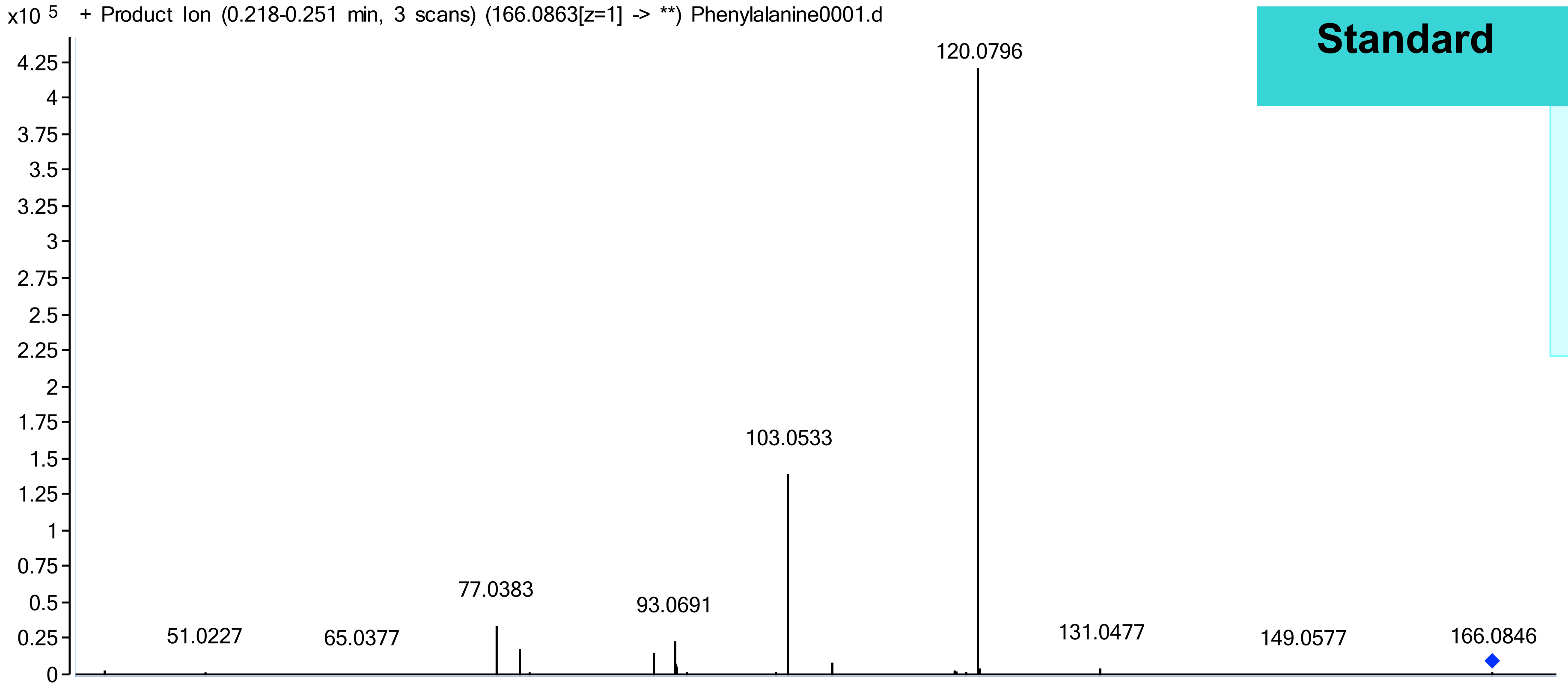

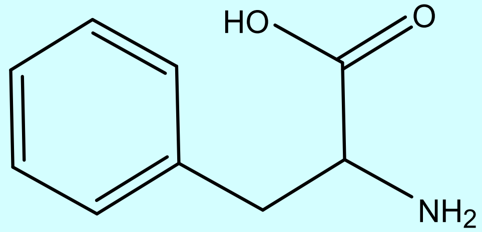

PHENYLALANINE

Formula:	$C_9H_{11}NO_2$
MM:	165.0790
MH$^+$:	166.0863

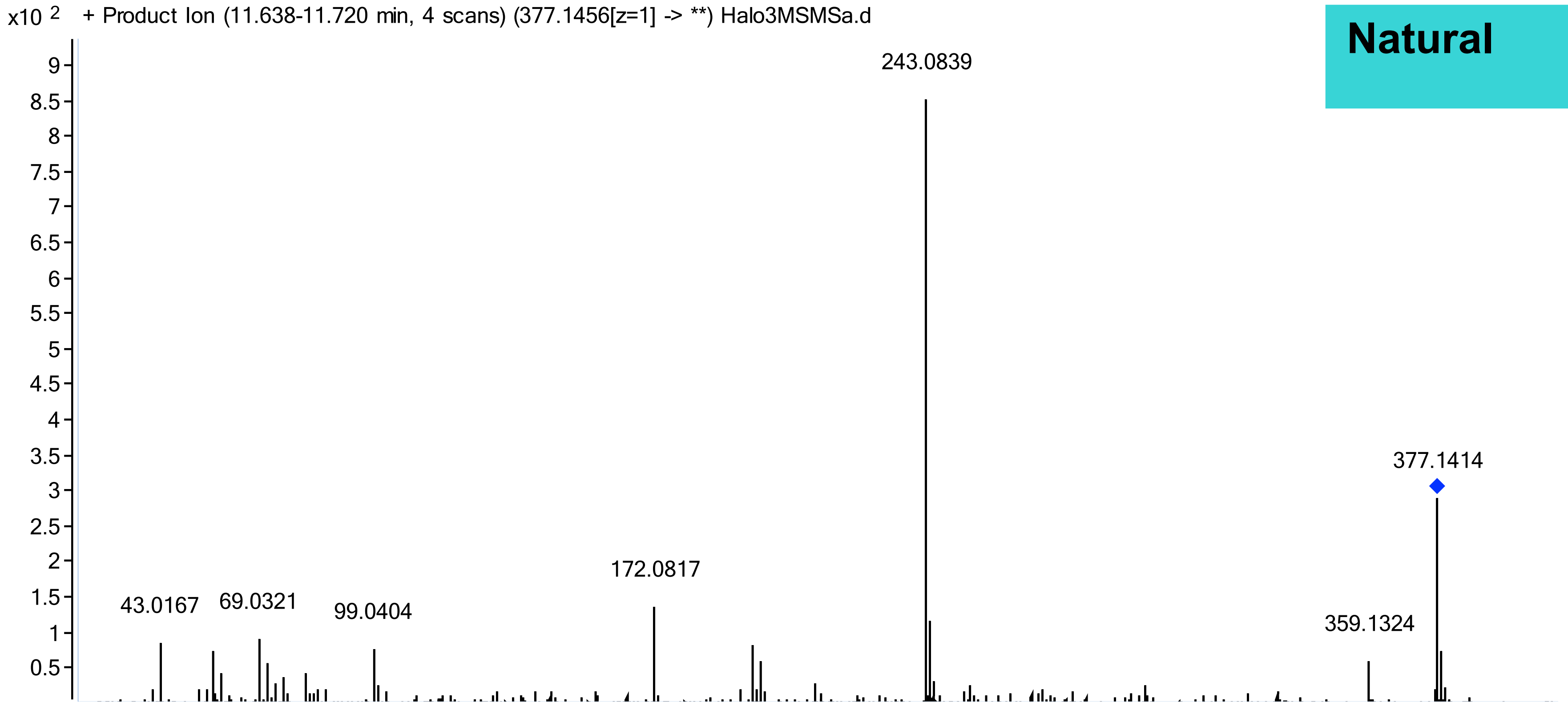

Natural

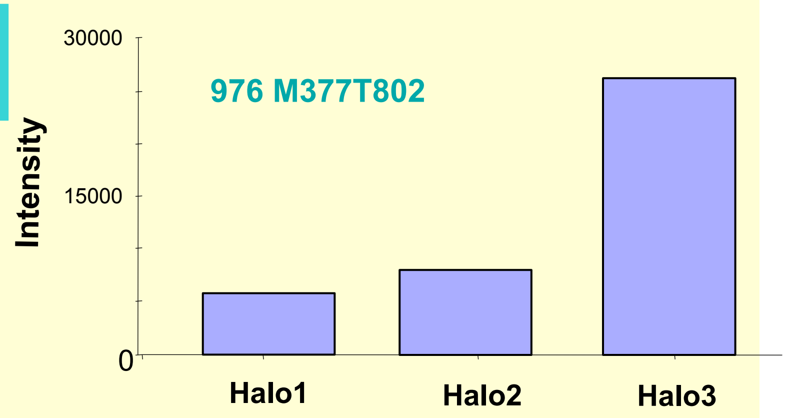

Retention time matches ~11.5 min

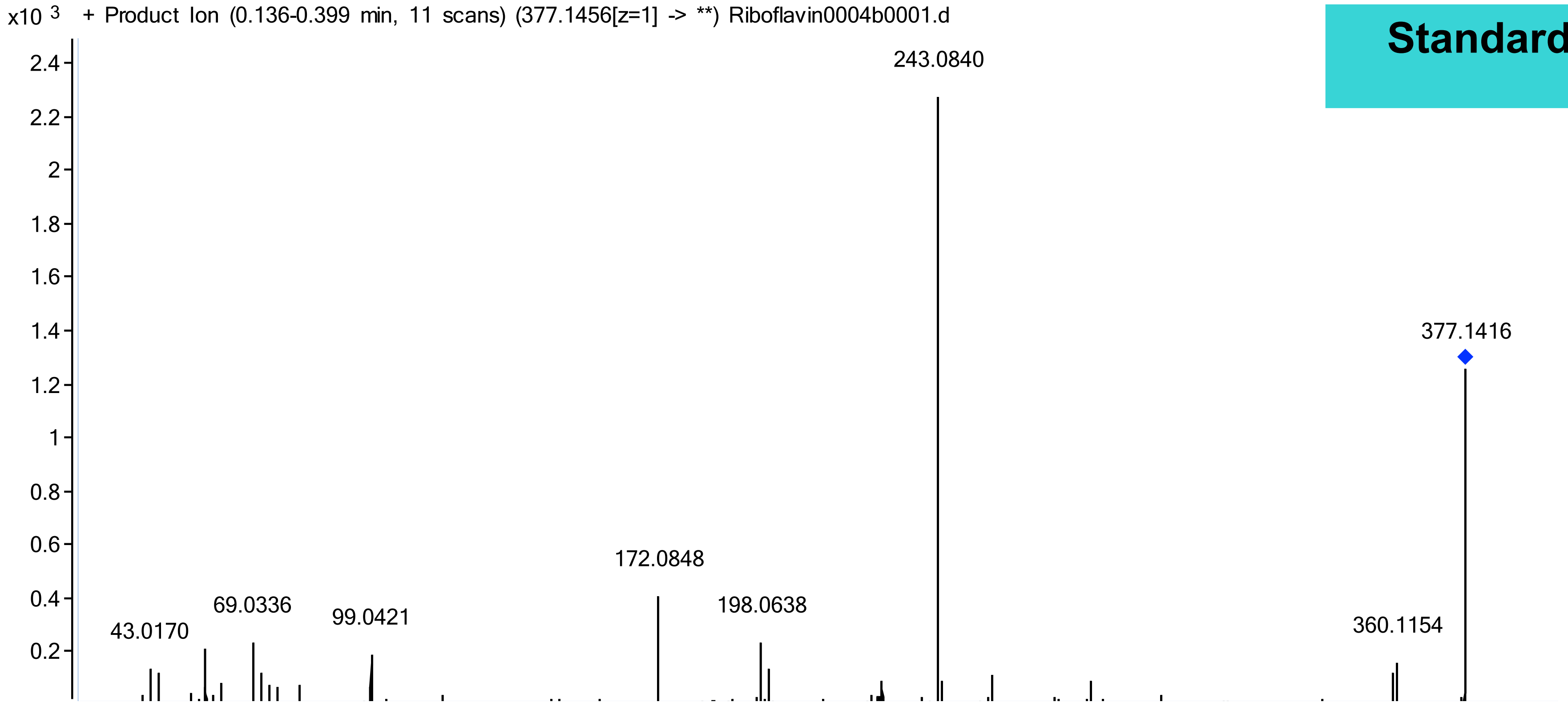

Standard

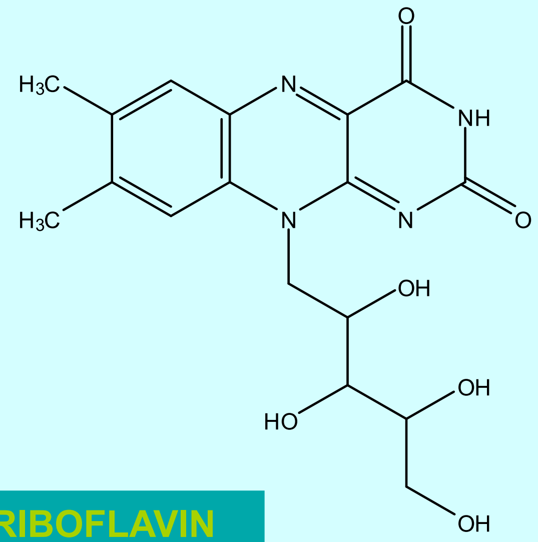

RIBOFLAVIN

Formula:	$C_{17}H_{20}N_4O_6$
MM;	376.1383
MH^+=	**377.1456**

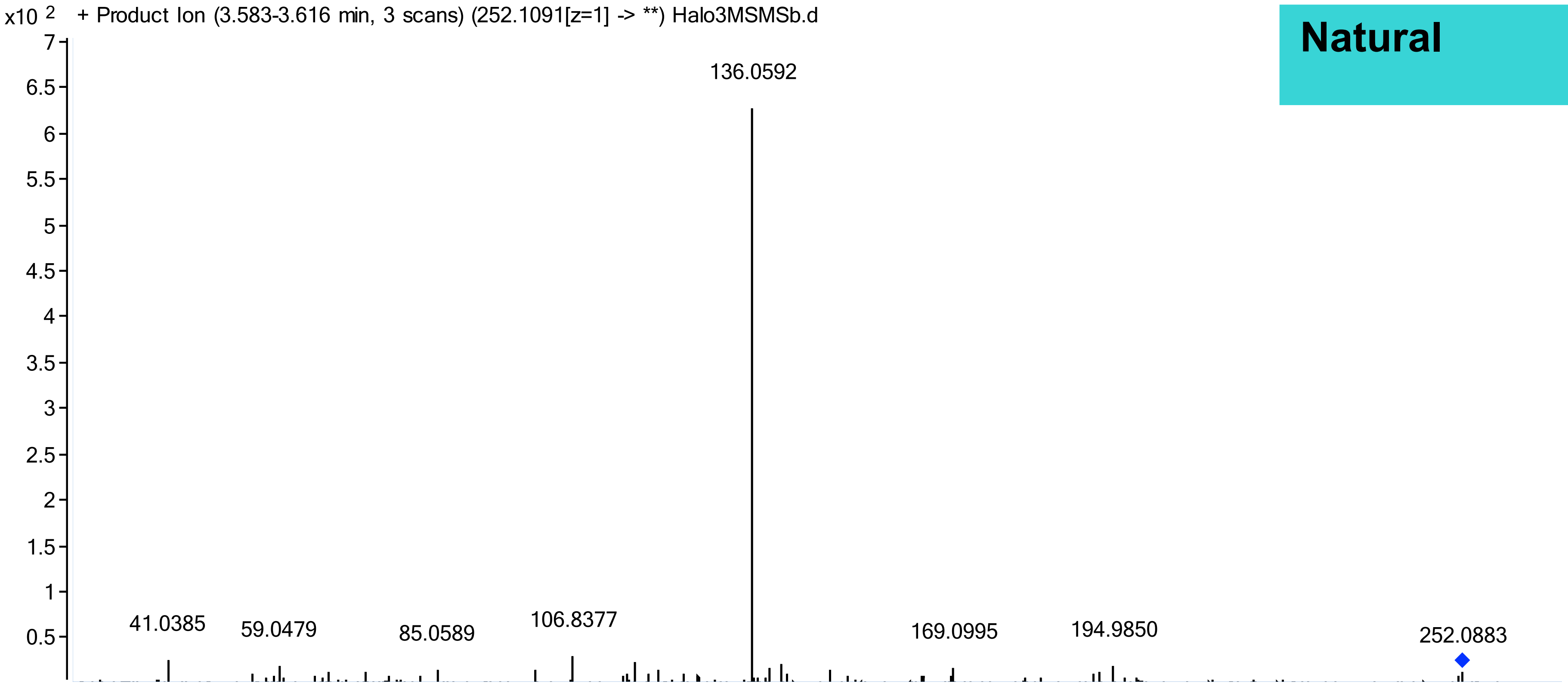

Natural

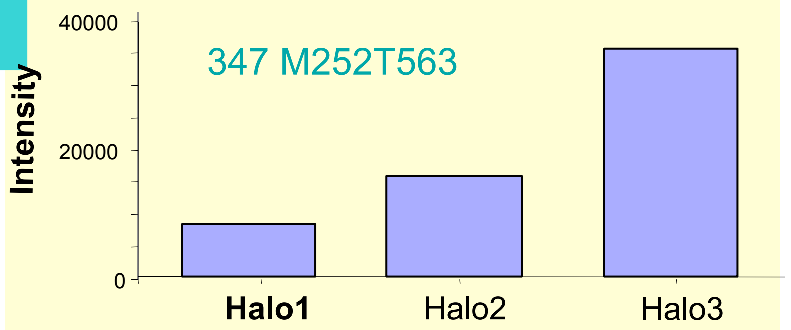

Retention time matches ~3.5 min

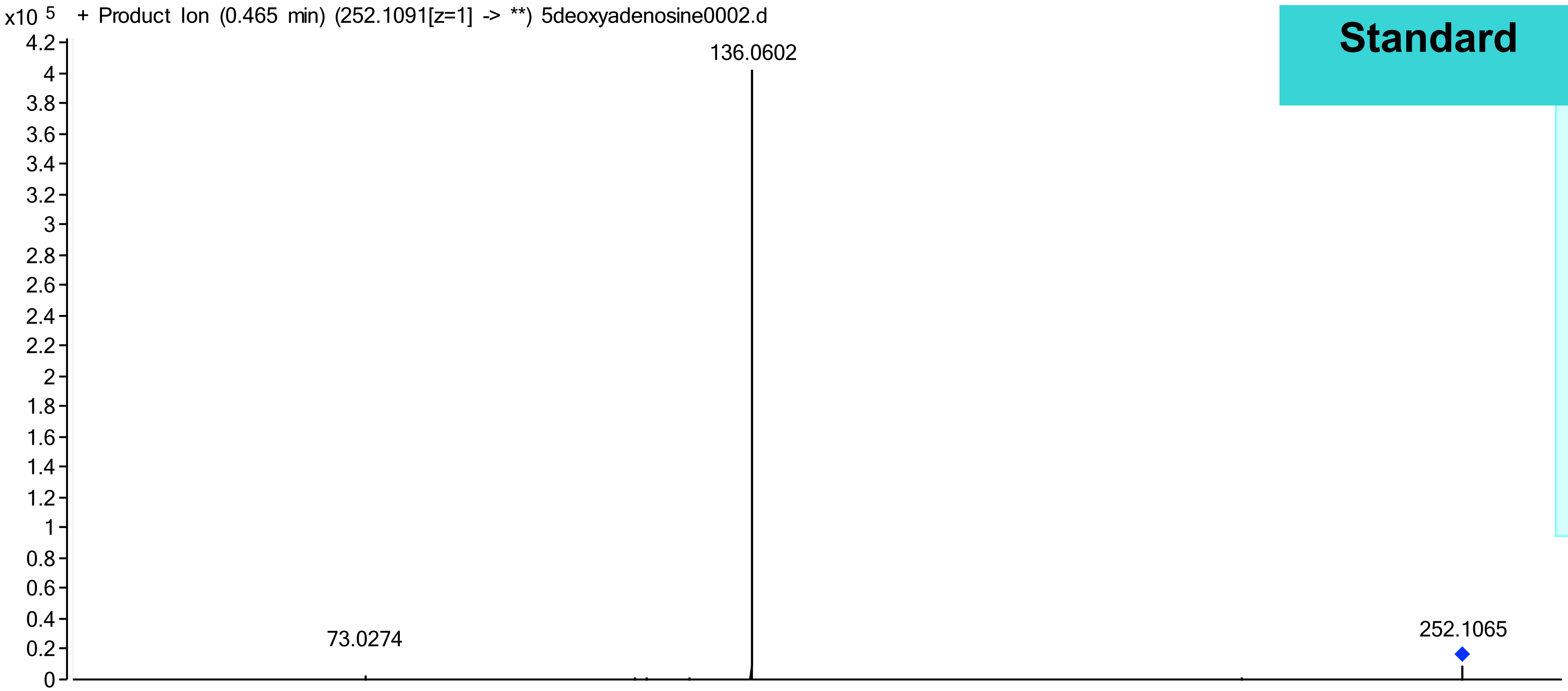

Standard

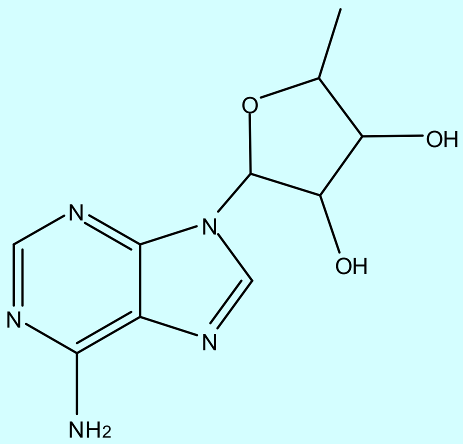

5-DEOXYADENOSINE

Formula: $C_{10}H_{13}N_5O_3$

MM= 251.1018

MH^+= **252.1091**
